# Evolutionary and expression analysis of *CAMTA* gene family in *Nicotiana tabacum* yielded insights into their origin, expansion and stress responses

**DOI:** 10.1038/s41598-018-28148-9

**Published:** 2018-07-09

**Authors:** Kaleem U. Kakar, Zarqa Nawaz, Zhouqi Cui, Peijian Cao, Jingjing Jin, Qingyao Shu, Xueliang Ren

**Affiliations:** 1 Molecular Genetics Key Laboratory of China Tobacco, Guizhou Academy of Tobacco Science, Guiyang, 550081 China; 2 State Key Laboratory of Rice Biology, Institution of Crop Science, Zhejiang University, Hangzhou, 310058 China; 3 Department of Microbiology, Faculty of Life Sciences & Informatics, Balochistan University of Information Technology, Engineering, and Management Sciences, Quetta, 87300 Pakistan; 4 Department of Plant Pathology and Ecology, The Connecticut Agricultural Experimental Station, New haven, CT 06511 USA; 5 Department of Biological Sciences, University of Wisconsin, Milwaukee, WI 53211 USA; 6 China Tobacco Gene Research Center, Zhengzhou Tobacco Research Institute of CNTC, Zhengzhou, 450001 China

**Keywords:** Comparative genomics, Gene ontology, Phylogenetics, Abiotic, Biotic

## Abstract

Calmodulin-binding transcription activators (*CAMTAs*) represent the novel gene family of transcriptional regulators, which play important biological functions. Though, the first ever plant *CAMTA* gene was evidenced in *Nicotiana tabacum* in 2002. But, the systematic identification, origin and function of this gene family has not been performed due to the lack of reference genome information until now. Here, we identified 29 *CAMTA* genes in four *Nicotiana* species, including thirteen *NtabCAMTAs*, six *NsylCAMTAs*, and five *NtomCAMTAs* and *NbenCAMTAs*. These CAMTA families were classified into five phylogenetic groups (I-V), among which, the group-IV CAMTAs probably emerged the earliest. The *NtabCAMTA* family genes have diverse structures, and are randomly localized on five chromosomes and scaffolds. *N*. *tabacum* acquired 11 copies of homolog *CAMATA* genes from the parental genomes of *N*. *tomentosiformis* and *N*. *sylvestris*, followed by expansion through polyploidization and duplication. The *NtabCAMTA* genes were differentially expressed in different plant parts, and showed sensitivity towards different abiotic and biotic stresses. Co-expression network analysis revealed that some NtabCAMTA subunits interact with each other, and co-expressed. The current study is the first report presenting a comprehensive overview of *Nicotiana CAMTA* families, and opens a new avenue for the improvement of the cultivated tobacco.

## Introduction

Calcium (Ca^2+^) ions act as ubiquitous secondary messengers for many cellular signaling pathways in eukaryotes^1^. Ca^2+^-mediated signal transduction is the key mechanism for transporting signals resulting from different stimuli, hence mediating growth, development and stress response in plants^2,3^. These nuclear and cytoplasmic Ca^2+^ signals are detected by different Ca^2+^-binding proteins such as Calmodulin (CaM), which upon binding to Ca^2+^, activates and alters the activity of CaM-binding proteins^4^. Transcription factors (TFs) regulated by Ca^2+^ or CaM are especially important in this phenomenon. So far, many TFs in plant are reported to interact with CaM^5^. Among the reported TFs, Calmodulin-binding transcription activators (*CAMTAs*) represent the latest and novel set of CaM-interacting proteins in plants. *NtER1*, the first plant and tobacco *CAMTA* gene was reported to be developmentally regulated and acts as a trigger for senescence and death^6^. Until now, the *CAMTAs* have been identified and reported in numerous plant species including *Arabidopsis thaliana*, rice, grapevine, cabbage and many more^7–10^. The plant *CAMTA*-encoded proteins comprise multiple functional domains, including CG-1, which is named after a partial cDNA clone isolated from parsley encoding a sequence-specific DNA-binding domain^11^, IPT/TIG (Ig-like, plexins, transcription factors or transcription factor immunoglobulin), ankyrin (ANK) repeats, and calmodulin-binding IQ motifs. These domains take part in protein–protein interactions, CaM binding, nonspecific DNA contacts in TFs and protein dimerization, respectively^12–14^. The *Arabidopsis CAMTA* family is comprised of six member genes, designated as *AtCAMTA1- AtCAMTA6*^15^. Latest studies have shown that these genes show quick and differential response to external stimuli, and are crucial for cross-talk between multiple signal transduction pathways involved in stress tolerance^6,8,16^.

*Nicotiana tabacum* (common tobacco) is chief commercial/cash crop, cultivated worldwide^17,18^. Enriched with alkaloid nicotine, tobacco leaves are largely used in cigarettes, cigars, chewing or smoking tobaccos and snuff. *N*. *tabacum* is used as a model plant organism and a key tool for plant molecular research, and a source of the BY-2 plant cell line to study primary biological processes^19,20^. It is also used as a model for plant disease susceptibility, which it shares with other *Solanaceae* plants including potato, tomato and pepper^18^. *N*. *tabacum* is an allotetraploid specie (2*n* = 4*x* = 48), most likely to be originated from a hybridization event (tetraploidization) between S (*N*. *sylvestris*) and T (*N*. *tomentosiformis*) genomes approximately 200,000 years ago^21^. *N*. *tabacum* therefore has a relatively large genome size (approximately 4500 Mb) compared with other cultivated *Solanaceae* crops^22^, and is 50% larger than the human genome.

So far, numerous varieties of tobacco have been domesticated and improved around the world including flue-cured, burley, oriental and cigar^23^. Similar to *N*. *tabacum*, *N*. *benthamiana* Domin (wild tobacco) is also an accepted model tobacco specie and has been widely used in experiments related to plant-virus response, protein localization, and plant-based systems for protein expression and purification^24,25^. Because of its complexity and larger size, the fully annotated reference genome sequence of *N*. *tabacum* was not available until now, which left behind large gaps in studying important biological pathways and gene families of tobacco including *CAMTA*. Taking advantage of the available genome data by Sierro *et al*.^18,26^. Edwards *et al*.^27^ and China tobacco (Ren *et al*. unpublished), we used comprehensive bioinformatics and experimental approaches to perform genome-wide identification and characterization of *CAMTA* gene family in *N*. *tabacum*, *N*. *sylvestris*, *N*. *tomentosiformis* and *N*. *benthaminana* species. To elucidate the evolutionary relationship between tobacco and other plants, we comprehensively analyzed the phylogeny between the orthologs of CAMTAs of four *Nicotiana* species and all plant lineages. Using the available RNA-seq data and Real-time quantitative PCR analysis, we quantified and analyzed the expression profiles of *NtabCAMTA* family genes during plant growth and development, and stress responses to different biotic and abiotic factors. This study will help to identify novel *CAMTA* genes for future breeding to improve plant production, quality and stress resistance, and open a new avenue for further elucidation for their roles underlying the signal transduction in tobacco.

## Results

### Genome-wide identification and domain analyses of *CAMTA* gene families in *Nicotiana*

To perform genome-wide identification and obtain the complete overview of *CAMTA* gene family in four *Nicotiana* species, a blast search in the tobacco genome sequences dataset was performed using *AtCAMTAs* as queries. Thirty-five candidate protein sequences were analyzed for the presence of CAMTA-specific conserved domains (CG-1: a sequence-specific DNA-binding domain; IPT/TIG: Ig-like, plexins, transcription factors or transcription factor immunoglobulin; ANK: ankyrin repeats; IQ: calmodulin-binding IQ motifs. As a result, six gene accessions having truncated amino acid sequences and/or lacking specific domains were discarded from analyses (Table S1). Finally, twenty-nine full length *CAMTA* genes having essential domains were identified from four *Nicotiana* species, including thirteen from *N*. *tabacum*, six from *N*. *sylvestris*, and five genes from *N*. *tomentosiformis* and *N*. *benthamiana*, respectively. These genes were named as *NtabCAMTAs*, *NsylCAMTAs*, *NtomCAMTAs* and *NbenCAMTAs*, based on their positions in phylogenies (Table 1 and Fig. S1).

**Table 1:** List and properties of 29 *Nicotiana CAMTA* genes identified in current study.

Species	Assigned ID	Accession	GeneBank ID	Location	Start	Stop	Strand	Transcript length	Protein length
*N*. *tabacum* (cultivated tobacco)	*NtabCAMTA1*	Ntab0183780	MF142771	Ntab_scaffold_1501	765092	775966	−	3186	1061
	*NtabCAMTA2*	Ntab0553680	MF142772	Chr06	12762522	12769907	−	3033	1010
	*NtabCAMTA3*	Ntab0473890	MF142773	Chr12	79813671	79824913	−	3312	1103
	*NtabCAMTA4*	Ntab0695330	MF142774	Ntab_scaffold_449	1491289	1503972	−	3324	1107
	*NtabCAMTA5*	Ntab0695280	MF142775	Ntab_scaffold_449	1022693	1035125	+	3285	1094
	*NtabCAMTA6*	Ntab0114010	MF142776	Chr02	137524070	137539662	−	3036	1011
	*NtabCAMTA7*	Ntab0794220	MF142777	Ntab_scaffold_62	2211509	2218668	+	2892	963
	*NtabCAMTA8*	Ntab0019010	MF142778	Ntab_scaffold_1041	47196	60650	−	3030	1009
	*NtabCAMTA9*	Ntab0852870	MF142779	Ntab_scaffold_725	1179782	1197497	+	3159	1052
	*NtabCAMTA10*	Ntab0354250	MF142780	Chr03	88953505	88964306	+	2955	984
	*NtabCAMTA11*	Ntab0045050	MF142781	Chr09	109877577	109892594	−	3051	1016
	*NtabCAMTA12*	Ntab0797190	MF142782	Chr02	122843834	122855979	−	2658	885
	*NtabCAMTA13*	Ntab0368180	MF142783	Chr02	113782109	113793963	−	2895	964
*N*. *tomentosiformis* (villous tobacco)	*NtomCAMTA1*	Ntom0062140	MH119951	Ntom_scaffold_159	726444	734619	−	3093	1030
	*NtomCAMTA2*	Ntom0118080	MH119952	Ntom_superscaffold_79	344968	357824	+	3453	1150
	*NtomCAMTA3*	Ntom0193400	MH119953	Ntom_superscaffold_10	1515969	1535693	+	2895	964
	*NtomCAMTA4*	Ntom0275970	MH119954	Ntom_scaffold_53	4882077	4902257	−	3150	1049
	*NtomCAMTA5*	Ntom0150930	MH119955	Ntom_scaffold_266	1027638	1042797	−	2907	968
*N*. *sylvestris* (woodland tobacco)	*NsylCAMTA1*	Nsyl0348080	MH119945	Nsyl_superscaffold_48	3136517	3145772	−	3132	1043
	*NsylCAMTA2*	Nsyl0152020	MH119946	Nsyl_superscaffold_480	854397	866667	+	3339	1112
	*NsylCAMTA3*	Nsyl0198310	MH119947	Nsyl_superscaffold_287	1976286	1996662	+	2988	995
	*NsylCAMTA4*	Nsyl0096530	MH119948	Nsyl_superscaffold_206	2459466	2470330	−	2895	964
	*NsylCAMTA5*	Nsyl0483140	MH119949	Nsyl_scaffold_996	2526	18288	+	3099	1032
	*NsylCAMTA6*	Nsyl0255340	MH119950	Nsyl_scaffold_2453	86407	96921	+	2892	963
*N*. *benthamiana* (wild tobacco)	*NbenCAMTA1*	Niben101Scf07773g00004		Niben101Scf07773	44708	60362	−	3689	1119
	*NbenCAMTA2*	Niben101Scf02268g06007		Niben101Scf02268	535556	549981	−	3872	1166
	*NbenCAMTA3*	Niben101Scf03911g05003		Niben101Scf03911	546981	561378	−	3679	964
	*NbenCAMTA4*	Niben101Scf01740g07004		Niben101Scf01740	771389	789257	+	3321	921
	*NbenCAMTA5*	Niben101Scf00380g00001		Niben101Scf00380	1432	20760	−	3278	757

**Figure 1:**
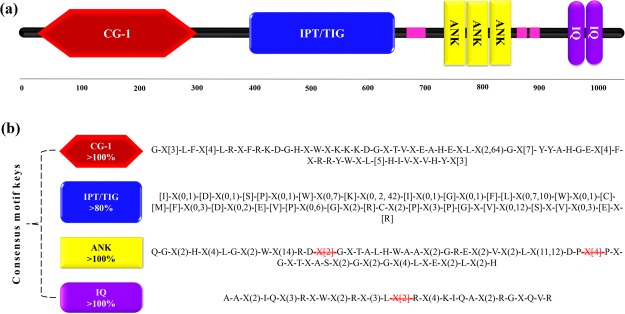
Graphical representation of primary domain architecture of *Nicotiana CAMTA*-encoded proteins **(a)** and their derived consensus motif keys **(b)**. The four CAMTA-specific domains are: CG-1 (a sequence-specific DNA-binding domain), IPT/TIG (Ig-like, plexins, transcription factors or transcription factor immunoglobulin), ANK (ankyrin) repeats, and calmodulin-binding IQ motifs. The square brackets “[]” indicate the amino acids allowed in this position of motif; “X” represents any amino acid, while round brackets “()” denote the number of amino acids. Letters highlighted as red strikethrough separates the two consecutive domains from each other.

The identified *Nicotiana* CAMTA proteins contained four conserved domains, namely CG-1, ANK repeats, IPT/TIG and IQ motifs, which are characteristic to plant CAMTAs (Fig. 1a). The occurrence of ANK domain varied from 1 to 3, and IQ from 2 to 3. Interestingly, two of *N*. *benthamiana* proteins (NbenCAMTA1 and NbenCAMTA2) contained an additional CG-1 domain, which requires further elucidation (Table S2). The multiple sequence alignment exhibited high sequence similarity between 29 CAMTAs, particularly in conserved domain regions. Among the four domains, TIG/IPT is the largest and less conserved (<80%) domain, whereas, CG-1 is interrupted by extra stretch of amino acids from NbenCAMTA1 and NbenCAMTA2 (Fig. S2). We derived stringent consensus motif keys showing the similarities and variances within the conserved domains of *Nicotiana* CAMTAs. Besides, these motifs can be used to identify and characterize the CAMTA proteins in *Nicotiana* species in future (Fig. 1b).

### Phylogenetic classification of *Nicotiana* CAMTAs

**Figure 2:**
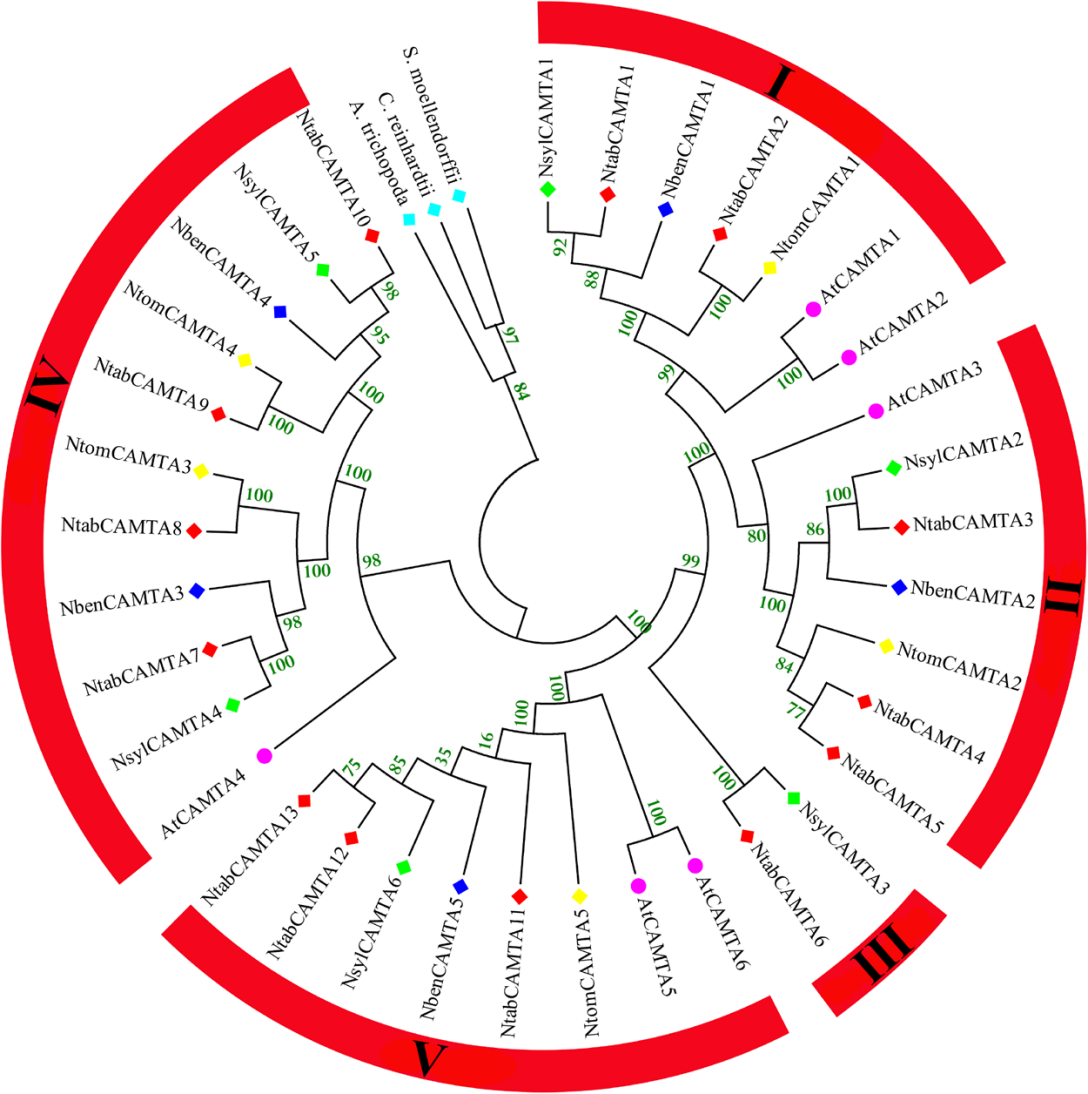
Phylogenetic analysis and classification of CAMTA family proteins identified in this study. The analysis involved 36 amino acid sequences, including 29 from 4 *Nicotiana* species identified in this study, 6 from *A*. *thaliana* and single from *A*. *trichocarpa* (AmTr_v1.0_scaffold00013.39) as outgroup. The tree with the highest log likelihood (−11525.8253) is shown. The bootstrap values from 1000 resampling are given at each node. Five groups were identified in tobacco CAMTA families, which were named as Group-I, II, III, IV and V. These groups were identified on the basis of *Arabidopsis* CAMTAs (marked with pink circles), while the members of each CAMTA family are shown with colored diamonds. Evolutionary analyses were conducted in MEGA 6.0.

To determine the homology between the *Nicotiana* CAMTA proteins, a rooted maximum likelihood (ML) phylogenetic tree of the 29 CAMTAs was constructed with 6 AtCAMTAs, using CAMTA proteins from *Amborella trichocarpa*, *Chlamydomonas reinhardtii* and *Selaginella moellendorffii* as outgroup. The inferred rooted tree produced well resolved phylogeny with high bootstrap or Bayesian support, showing that *Nicotiana* CAMTA family proteins can be classified into five major clusters/groups. Of these five groups, Group I, II, IV and V fall into the phylogenetic classification of *Arabidopsis* CAMTA family, hence named accordingly. However, two *Nicotiana* CAMTA proteins (i.e., NtabCAMTA6 and NsylCAMTA3) clustered separately, hence placed into a separate group III (Fig. 2). Group-IV comprised ten *CAMTA* genes, thus making the largest clade, while, group-III was the smallest clade with two genes only. The best ML scoring rooted tree topology indicated that during evolution Group-IV CAMTAs probably emerged the earliest among all in *Nicotiana*, while, Group I, II, III and V as the latest respectively (Fig. 2).

### Evolutionary relationship between *Nicotiana* and other plant CAMTAs

To determine the evolutionary relationship between tobacco and other plants, we comprehensively analyzed the phylogeny between single orthologs of CAMTAs of four *Nicotiana* species and all plant lineages. For the purpose, we identified 56 CAMTA sequences from different species including green algae, bryophytes, lycophytes, gymnosperms, monocots and dicots, whose protein sequences harbored typical domains and motifs of CAMTA proteins (Table S3).

At least one species from all plant lineages and main groups was selected. The resultant tree contained five major groups with significant bootstrap values, showing the expansion of *CAMTA* genes in Group I. According to the tree’s topology CAMTAs of all plants share a common ancestor, where the lower plants settled in the basal group. *NtabCAMTAs* found its place among other *Solanaceous* plants (*S*. *lycopersicum* and *S*. *melongena*) in Group I. These analyses also revealed that *CAMTA* gene family expansion occurred conspicuously greater in eudicots among all plant lineages (Fig. S3). In many previous studies expansion in gene families due to duplications has been discussed^28–33^.

### The *NtabCAMTA* gene family

There are thirteen genes in the genome of cultivated tobacco (*NtabCAMTA1-13*). The nucleotide lengths of these genes varies between 2658 bp (*NtabCAMTA12*) and 3324 bp (*NtabCAMTA*4), while, their encoded protein lengths ranges between 885 (NtabCAMTA12) and 1107 amino acids (NtabCAMTA4) (Table 1). The *NtabCAMTA* family genes are randomly scattered throughout the *N*. *tabacum* genome. As shown in Table 1, seven *NtabCAMTA* genes are localized on five chromosomes, while five genes are clustered on six scaffolds. Three *NtabCAMTA* genes are localized on chromosome 2, and chromosomes 3, 6, 9 and 12 has only one gene. Nine of the NtabCAMTA genes are positioned on reverse strands of the chromosomes/scaffolds, and four genes are oriented on forward strand. Alignment between *N*. *tabacum* and *Arabidopsis CAMTA-*encoded proteins showed that NtabCAMTAs have high similarity with AtCAMTAs, especially in CG-1, ANK and IQ regions. In each region, more than 50 residues stretches are conserved, where the differences show the divergence across the two species (Fig. S4). The phylogenetic analyses of NtabCAMTA with *Arabidopsis* CAMTAs resulted into similar clustering pattern of five groups, where, Group-I contained 2 *NtabCAMTA* genes, Group-II and V had 3 genes each, Group-III had one gene, while, Group-IV comprised 4 genes (Fig. S5).

The ProtParam tool showed that NtabCAMTA proteins greatly differ in molecular weights (ranging from 99.453 to 124.089.02 kDa), consistent with the number of atoms present. Nearly all of the NtabCAMTA proteins have relatively low isoelectric points (pI < 9), and are hydrophilic. Comparatively, NtabCAMTA4 is the most hydrophilic, while NtabCAMTA6 is the least hydrophilic protein. The aliphatic index showed that most NtabCAMTA proteins are thermostable as other globular proteins. According to the instability index (II), only two proteins (NtabCAMTA4 and NtabCAMTA12) could be classified as stable in the test tubes. In addition, NtabCAMTA proteins, except NtabCAMTA11 and NtabCAMTA12, have more negatively charged residues (aspartic acid/glutamic acid) as compared to positively charged residues (arginine/lysine) (Table 2).

**Table 2:** Physico-chemical properties of 13 *NtabCAMTA*-encoded proteins.

Proteins	Accession	Molecular weight (kDa)	pI	(Asp + Glu)	(Arg + Lys)	atoms	II	Aliphatic index	GRAVY
NtabCAMTA01	Ntab0183780	118.435	5.62	148	119	16456	44.68	79.83	−0.44
NtabCAMTA02	Ntab0553680	113.184	5.45	147	114	15706	44.27	77.88	−0.509
NtabCAMTA03	Ntab0473890	123.614	5.7	150	120	17131	42.89	72.32	−0.627
NtabCAMTA04	Ntab0695330	124.089	5.68	153	121	17180	42.4	71.89	−0.639
NtabCAMTA05	Ntab0695280	122.641	5.68	151	119	16980	42.16	71.85	−0.636
NtabCAMTA06	Ntab0114010	112.848	8.44	120	127	15855	38.01	87.96	−0.37
NtabCAMTA07	Ntab0794220	107.827	5.79	128	108	14978	44.62	76.19	−0.523
NtabCAMTA08	Ntab0019010	112.827	6.37	126	117	15661	47.74	73.2	−0.556
NtabCAMTA09	Ntab0852870	116.988	7.2	127	126	16285	44.07	74.53	−0.525
NtabCAMTA10	Ntab0354250	109.923	6.25	121	111	15311	46.16	78.4	−0.464
NtabCAMTA11	Ntab0045050	114.699	7.58	120	121	16004	40.55	78.98	−0.396
NtabCAMTA12	Ntab0797190	99.453	7.64	106	107	13844	38.51	73.61	−0.5
NtabCAMTA13	Ntab0368180	108.826	6.79	117	113	15151	41.15	75.87	−0.454

### Origin and expansion of *NtabCAMTA* family genes

The phylogeny between *N*. *tabacum* and its ancestors (*N*. *sylvestris* and *N*. *tomentosiformis*) provided clear picture of how the *NtabCAMTA* gene family originated and expanded, and to detect the retention and/or loss of *CAMTA* genes after genome duplication and polyploidization. Since T and S genomes contain 5 and 6 *CAMTA* genes respectively, which are likely to produce 10, 11 or 12 genes in *N*. *tabacum*, if the ancestral genes are to be counted 5 or 6. As of now, there are 13 *CAMTA* genes in *N*. *tabacum*. As shown in Fig. S6, 11 of these *NtabCAMTA* genes have clear single ancestor in phylogenetic tree, showing that each gene has been acquired from single parent and retained. Two gene pairs (i.e., *NtabCAMTA4/NtabCAMTA*5 and *NtabCAMTA12/NtabCAMTA13*), showing high resemblance at nucleotide level (92.12 and 95.24%) as well as at amino acid level (98.64 and 91.69%), seem to be originated by duplication event, which might have occurred after the divergence from parental species (Fig. S6). Two accessions (Ntab0503030and Ntab0966680), which were discarded during preliminary analyses, seem to be redundant copies that probably lost their functions during the long course of evolution. However, this presumption requires experimental validation. Together, these finding suggest that polyploidization, along with gene duplication played important role in expansion of *CAMTA* gene family in *N*. *tabacum*.

### Structures of *NtabCAMTA* family genes and conserved motifs in their*-*encoded proteins

**Figure 3:**
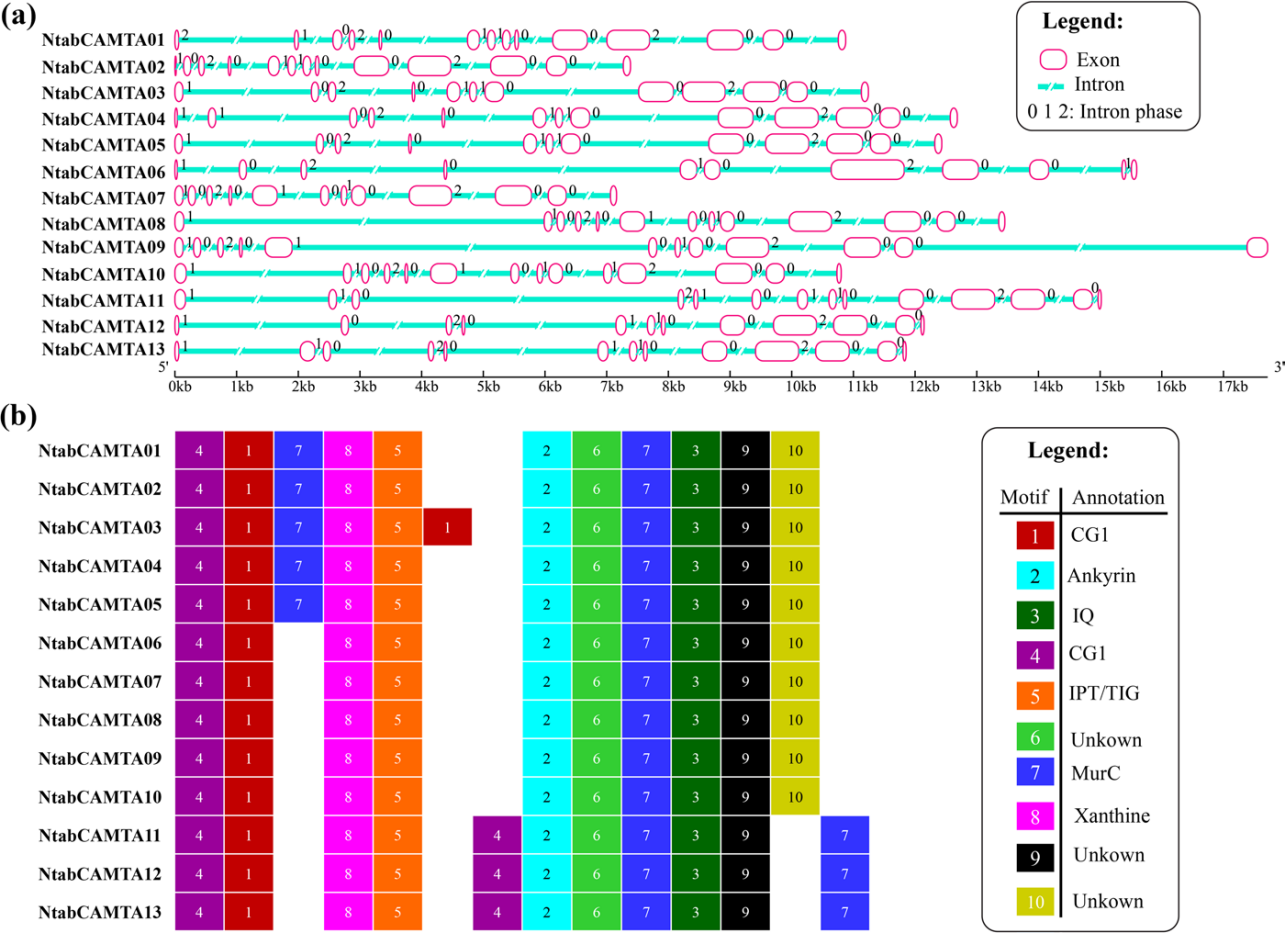
Schematic diagram representing the structures of *NtabCAMTA* genes and the distribution of conserved motifs in associated proteins. **(a)** Gene structures showing the organization of exon and intron structures, and associated intron phases [0, 1 and 2] of 13 *NtabCAMTA* genes. The NJ phylogenetic tree of CDs is shown on the left side of the figure. (b) The distribution of conserved motifs identified in *NtabCAMTA*-encoded proteins. Each motif is represented by a colored box, and their names given at the bottom of diagram. The logos and annotations of functionally defined motifs are given in Fig. S9 and Table S4, respectively. The order of motifs corresponds to their positions in protein sequence, however, the length of the boxes does not correspond to the lengths of motifs.

To characterize the structural diversity of the *NtabCAMTA* family, exon-intron organization analysis of the individual gene was performed (Fig. 3a). The majority of the *NtabCAMTA* genes contain twelve or thirteen exons, where *NtabCAMTA1*, *NtabCAMTA10 and NtabCAMTA*11 contain highest number of exons (14). Most of the introns in *NtabCAMTA* genes are in intron phase 0 (77), interrupted by exact triplet codons. Twenty-seven phase-II introns (separated by 2^nd^ and 3^rd^ codons) were observed in *NtabCAMTA* family genes, where each gene comprise two phase-2 introns, except for *NtabCAMTA*1, which contain three phase-2 introns. Forty-eight single phase-I introns were detected in *NtabCAMTA* genes, where each gene contain 3 or 4 single phase-I introns. Several changes in terms of loss/gain of exons, intron phases and their shuffling was observed between the structures of genes belonging to same phylogenetic group, thus adding diversity to both the structures and functions of *NtabCAMTA*s (Fig. S7). We observed that *NtabCAMTA*4 and *NtabCAMTA*12 of the duplicated gene pairs (*NtabCAMTA*4/5 and *NtabCAMTA*12/13) have lost their single phase-I intron and exon subsequently, unlike *NtabCAMTA*5 and *NtabCAMTA*13 that have similar exon numbers, intron phases and lengths. This suggest that *NtabCAMTA*4 and *NtabCAMTA*12 genes arose from *NtabCAMTA*5 and *NtabCAMTA*13, which evolved from parental genes. Besides, *AtCNGCs* comprise more phase-I introns than tobacco homologs, showing that intron loss during the long course of evolution resulted into reduced numbers of introns in *NtabCAMTA* family genes, particularly in members of Group I-V (Fig. S8).

When submitted to online MEME server (Multiple Expectation Maximization for Motif Elicitation), the NtabCAMTA proteins were found to contain at least ten conserved motifs. Among these, seven motifs (motif 1–5, 7 and 8) are part of the known domains, as shown by Pfam codes and WebLogos (Fig. 3b; Fig. S9 and Table S4). Motif 1 and 4 are associated with CG-1 domain; motif 2 is the longest motif of 59 residues that correlate with ankyrin repeat profile or ANK domain; motif 3 is 50 residues long motif associated with IQ domain; motif 5 represent IPT/TIG domain, and contain phosphorylation sites for casein kinase II (CK2), protein kinase C. On other hand, two motifs (i.e., motif 7 and motif 8) are not correlated with known domain in pfam, however, their secondary association can be linked with the MurC (UDP-N-acetylmuramate-alanine ligase [Cell envelope biogenesis, outer membrane]) and xanthine phosphoribosyltransferase, respectively (Table S4). The functionality of the remaining motifs (6, 9 and 10) is still unknown, and awaits further experimental proof.

### Potential microRNA target sites in *NtabCAMTA* transcripts

Identifying the target sites in gene transcripts provide valuable information regarding the role of miRNAs in plant growth, signal transduction pathways and stress responses. Analysis of 164 published tobacco microRNAs^34^ revealed that *NtabCAMTA4* and *NtabCAMTA5* contain the target sites for two miRNAs (i.e., nta-miR6164a and nta-miR6164b) (Table 3). Keeping cut-off threshold of 4.5 in the search parameter, which give higher prediction coverage compared to default threshold of 3.0, we identified 8 miRNAs (6 families such as nta-miR159, nta-miR394, nta-miR395, nta-miR477, nta-miR6163 and nta-miR6020b) containing target sites in 7 *NtabCAMTA* transcripts with the same expectation score (Table S5). The nucleotide lengths of these miRNAs was 21nt, with more stringent cut-off threshold (1.5) showing lower false positive prediction^35^. These miRNAs are located on the 3′ arm of the stem-loop hairpin structures. The UPE (target accessibility of target site), which is key feature in target identification and exhibit energy required to contact (and cleave) target mRNA, varied from 10.5 (nta-miR6164a) to 16.8 (nta-miR6164a), where lower energy reflects the higher possibility of contact between miRNA and target site. Two miRNAs were found to be involved in cleavage of the target transcript. The nta-miR6164 class of miRNAs has been reported to be involved in wounding and topping stress response in tobacco^34^.

**Table 3:** The potential miRNA targets in the set of 13 *NtabCAMTA* transcripts.

miRNA Acc.	Target Acc.	Expectation (E)	Target Accessibility (UPE)	Alignment	Inhibition	Multiplicity
nta-miR6164a	NtabCAMTA4	1.5	16.80		Cleavage	1
nta-miR6164a	NtabCAMTA5	1.5	12.67		Cleavage	1
nta-miR6164b	NtabCAMTA4	1.5	10.52		Cleavage	1
nta-miR6164b	NtabCAMTA5	1.5	13.4		Cleavage	1

### GO enrichment analysis

Using Blast2GO (v.3.3.5), we were able to assign total of 78 gene ontology (GO) classes to 13 *NtabCAMTA* genes with blast matches to known proteins in InterPro. Of these, majority were assigned to biological process (35), followed by molecular function (32) and cellular components (10). All genes were found to be integral components of membrane or localized in nucleus (Table S6). These proteins are involved in molecular processes associated with kinase activities, nucleotide, proteins, ions/receptors binding, and the regulation of transferase activities (Table S7). Notably, these *NtabCAMTA* genes were associated to with GO-terms for numerous biological processes including regulation of gene expression, transport, signal transduction, response to stimulus, anatomical structure development, cell differentiation and other developmental processes (Table S8).

### *In planta* expression of CAMTA genes in tobacco tissues

**Figure 4:**
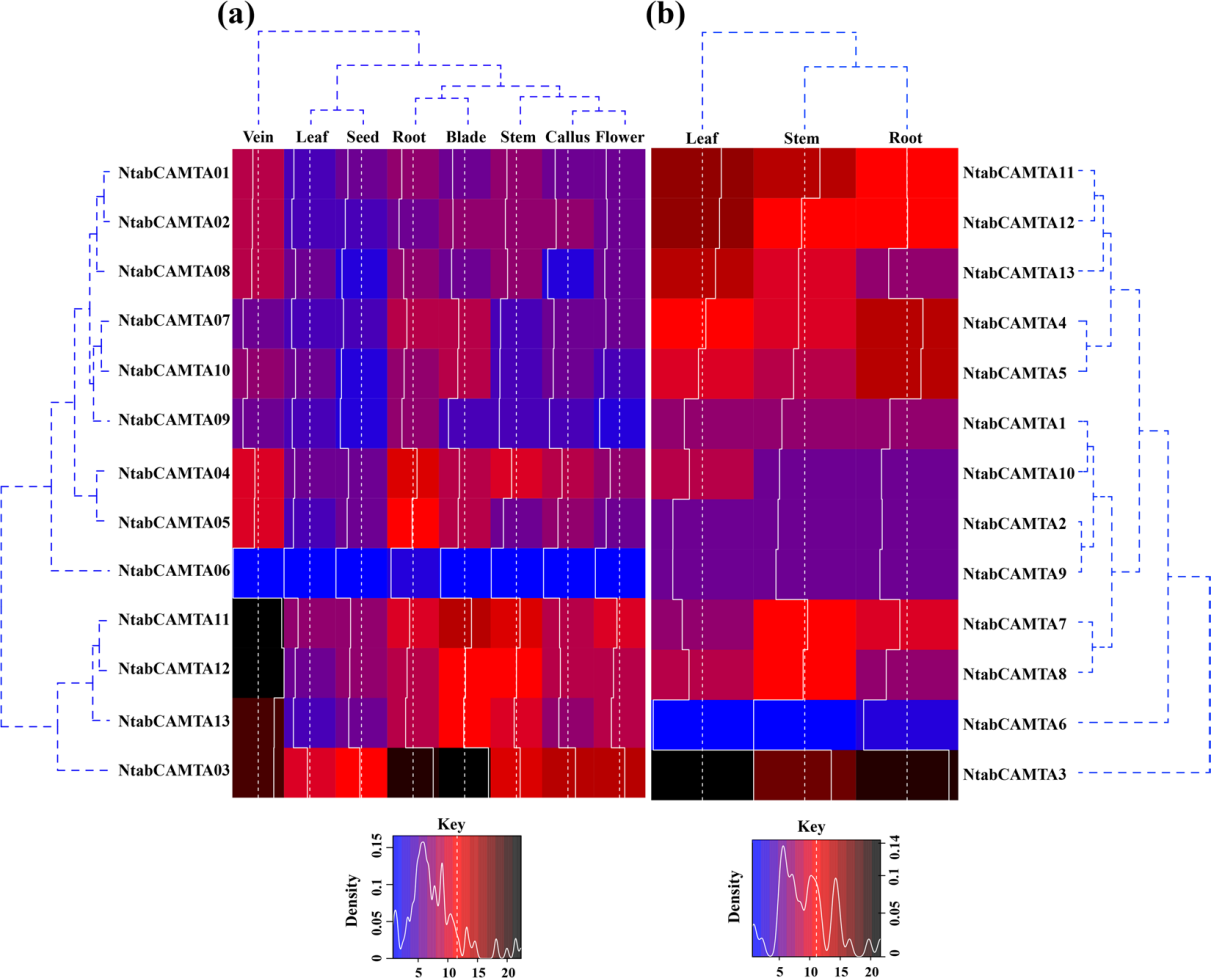
Expression profiles of *NtabCAMTA* genes in different plant parts of *N*. *tabacum* cultivar: TN90. (**a**) Normalized expression levels (FPKM log2) at early stage of tobacco growth. **(b)** Normalized expression levels (FPKM log2) in post-harvested tissues of mature tobacco. The gene names and cluster tree are indicated on the left. The intensity of transcript abundance is indicated with different colors (blue = lower accumulation, black = higher accumulation) and white histograms within the heatmap.

To get insight into the steady-state expression of *NtabCAMTA* genes, we utilized the transcriptomic RNA-seq data from Sequence Read Archive in GeneBank (SRP029183) that were reported previously^18^. The generated RNA-seq data included the expression profiles of seed, leaf (whole leaf, vein and blade), stem, callus, root and flower bud of *N*. *tabacum* TN90 at five leaf stages. The final expression data of 13 *NtabCAMTA* genes was log transformed and illustrated in heatmap (Fig. 4a). Among all, eight *NtabCAMTA* genes were significantly expressed at relatively higher levels in at least one tissue, including five in leaves, four in roots, leaf blade and stem tissue, two in flower, and single gene was expressed in seed and callus respectively. Five genes namely *NtabCAMTA1*, *NtabCAMTA2*, *NtabCAMTA6*, *NtabCAMTA9* and *NtabCAMTA10* did not expressed in any tissue. Further investigation revealed that expression of *NtabCAMTA3* was up-regulated in all studied plant parts, where it showed maximum level of expression in callus, seed, root, stem, leaf blade and flower bud, demonstrating its importance in tobacco plant growth and development. Among the other genes, *NtabCAMTA11* and *NtabCAMTA*12 showed highest levels of expression in leaf vein, followed by *NtabCAMTA*13 and *NtabCAMTA3* respectively. Post-topping RNA-seq data showed that the members of phylogenetic group-II and group-IV were particularly induced in root and leaf tissues (Fig. 4b). Five genes, including *NtabCAMTA3*, *NtabCAMTA7*, *NtabCAMTA8*, *NtabCAMTA10* and *NtabCAMTA11* were induced in stem tissues, showing their induction under wounding. Taken together, *NtabCAMTA* genes exhibited differential expression pattern in different tissues and several genes are induced by wounding in tobacco genome. Higher expression in same tissue indicated their functional conservation, but others point toward their functional diversities.

### Responses to biotic stress

**Figure 5:**
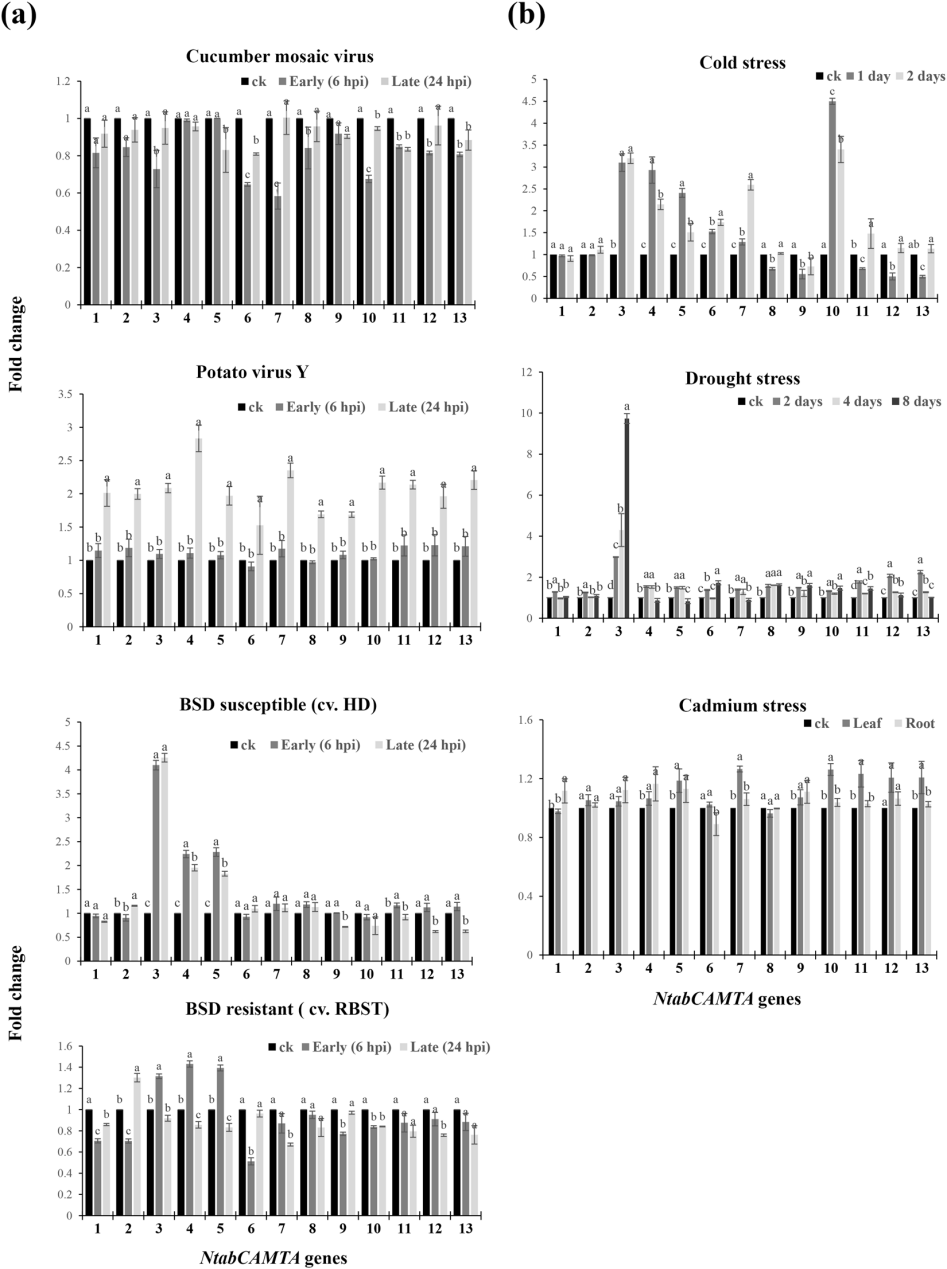
Results of RT-qPCR analysis showing the expression patterns of *CAMTA* genes in tobacco plant subjected to external stress at different time points. (**a**) Biotic stress response to CMV, PVY and BSD (left panel). (**b**) Abiotic stress response to cold, drought and cadmium (right panel). The names of *NtabCAMTA* genes are shown with the numbers in X-axis, while Y-axis show the relative expression levels or fold-changes of treatments versus control (ck). Bars with same letter means no significant difference based on LSD test (p ≤ 0.05). For experimental details see Materials and Methods.

To get insight into the role of *NtabCAMTAs* in disease resistance and host stress response, a RT-qPCR analysis was performed on tobacco seedlings (having four fully expanded leaves) exposed to different phytopathogens, using mock inoculum as control. The pathogens used in this experiment included viral pathogens *Cucumber mosaic virus-*M (M strain of CMV) and *Potato virus* Y (Mn strain of PVY), and a fungal pathogen black shank or *Phytophthora nicotianae*^36,37^. The results showed that the *NtabCAMTA* genes under study differentially responded to each pathogen understudy at certain time point (Fig. 5). In CMV infected tobacco seedlings, the expression levels of the *CAMTA* were slightly but significantly repressed at 6 hpi and 24 hpi showing early and late responses. After 6 hpi, the expression levels of eight *NtabCAMTA* genes were induced and down-regulated compared to control. Only five genes showed down-regulation after 24 hpi. Compared to control, maximum positive response to CMV (fold-change) was noted for group-IV *NtabCAMTA* genes (Fig. 5a). After inoculation with PVY, the expression of all *NtabCAMTA* genes was increased after 24 hpi. None of the *NtabCAMTA* genes showed early response at 6 hpi. Overall, maximum response to PVY-Mn was shown by *NtabCAMTA4* that was increased by >2.8-folds after 24 hpi, followed by *NtabCAMTA7*, which was >2.35-folds up-regulated after 24 hpi compared with control (Fig. 5a).

Black shank disease (BSD), caused by the oomycete pathogen *P*. *nicotianae*, is a devastating root rot disease of tobacco^38^, causing wilting and yellowing of leaves, along with dark brown to black spots at the base of the plant stem. We calculated the expression levels of *NtabCAMTA* genes in the leaves of susceptible tobacco variety (i.e., Hong hua Da jin yuan or HD) and resistant cultivar (i.e., Resistance to Black Shank Tobacco or RBST) inoculated with *P*. *nicotianae* for 6 or 24 h. The data depicted that most *NtabCAMTA* genes were early induced by *P*. *nicotianae* inoculation. The group-III genes (i.e., *NtabCAMTA3*, *4* and *5*) exhibited almost similar trend of up-regulation in both cultivars, where the intensity of gene expression was higher in *N*. *tabacum* cv. HD than the *N*. *tabacum* cv. RBST. Moreover, the expression of *NtabCAMTA2* gene was significantly induced after 24 hpi in both cultivars. Meanwhile, maximum response was noted for *NtabCAMTA3* that was >4-times up-regulated in *N*. *tabacum* cv. HD. On other hand, three genes were down-regulated by *P*. *nicotianae* inoculation in *N*. *tabacum* cv. HD, and nine genes were negatively induced in *N*. *tabacum* cv. RBST (Fig. 5a).

Over all the transcription data demonstrated that *NtabCAMTAs* are amongst the early genes to sense and respond to biotic stress, and their regulation play important roles in plant defense.

### Responses to abiotic stress

To assess the role of *NtabCAMTA* genes in response to abiotic stress, we investigated their expression patterns in the leaves of tobacco seedlings subjected to cold stress at 4 °C for 1–2 days, and drought stress for 2, 4 and 8 days, respectively. Additionally, we quantified expression profiles of *NtabCAMTA* genes in leaf and root samples of tobacco plants subjected to cadmium stress (Cd: 250 mM) for 1 day. Under cold stress, the expressions of seven genes (i.e., *NtabCAMTA3*-*7*, *10* and *11*) significantly increased at each time point, and those of five genes decreased after 2 days, compared to control. On other hand, most of the *NtabCAMTA* genes were up-regulated after 2 day of drought stress. Comparatively, the maximum up-regulation was noted for *NtabCAMTA3* that was >9-folds increased after 8 days of drought stress. Among others, group-V *NtabCAMTA* genes were differentially expressed, depending on the time scale (Fig. 5b).

Under Cd stress, four *NtabCAMTA* genes were significantly expressed in leaf tissues, while seven genes were expressed in roots. Only one gene (i.e., *NtabCAMTA*6) was significantly down-regulated in tobacco roots. The remaining five genes (i.e., *NtabCAMTA1-3*, *8* and *12*) did not exhibit significant expressional response to Cd stress in terms of fold-change compared to control. Overall, the expression response of *NtabCAMTA* genes considerably varied among individual groups, suggesting the importance of the *CAMTA* genes in the survival of tobacco plants under different abiotic stress conditions (Fig. 5b).

### Co-expression network analysis

**Figure 6:**
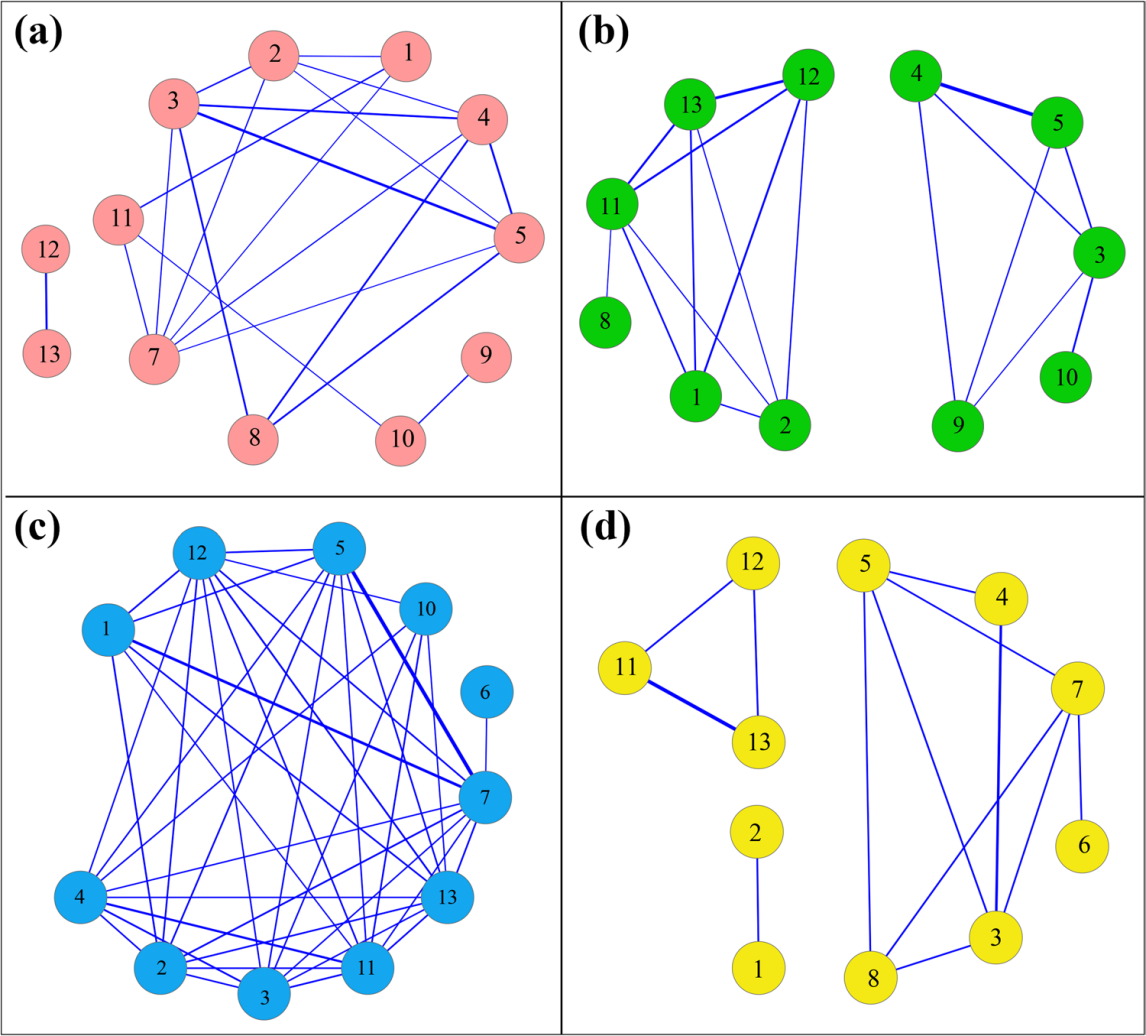
Co-expression networks of *NtabCAMTA* genes showing global view of dynamical and conservative expression profiling. (**a**) Overall co-expression network of *NtabCAMTA* genes using combined expression data. (**b**) Co-expression network using in-planta RNA-seq data. (**c**) Co-expression network under biotic stress. (**c**) Co-expression network under abiotic stress. The diagrams were prepared using cytoscape. The edge line width represents the Pearson correlation coefficient (r) with the value of “r” ranging between 0.7 to 1. *NtabCAMTA* genes showing dynamic expressions are not indicated in the diagram.

To enhance the presentation of the dynamical and conservative expression profiling of *NtabCAMTA* genes, we performed gene co-expression networks analyses for all members of this gene family by means of the RNA-Seq and RT-qPCR analysis data. A global view of co-expression network based on Pearson’s correlation coefficient threshold of 0.75, we found that the expression patterns of 12 *NtabCAMTA* genes closely correlated (Fig. 6a). Among these, *NtabCAMTA12* and *13* independently co-expressed with each other. The *NtabCAMTA6* gene, which belong to a separate clade of phylogenetic Group-III, did not show correlation with other *NtabCAMTA* genes. We further constructed three more networks with Cytoscape to display the relationships between *NtabCAMTA* genes, which differentially expressed under different conditions (Fig. 6b–d). The expression patterns of eleven *NtabCAMTA* genes were significantly correlated with each other in different plant tissues (Fig. 6b). Our analysis also indicated that some members of the phylogenetic Groups-IV (i.e., *NtabCAMTA7–10*) did not co-expressed in biotic and abiotic stress conditions (Fig. 6c,d). On other hand, *NtabCAMTA* genes belonging to Group-I and IV, co-expressed in group specific manners under abiotic stress (Fig. 6d). Overall, most of these genes displayed similar expression patterns and the results suggested the possibility that NtabCAMTA subunits interact with each other under different conditions.

## Discussion

Bioinformatics tools and publicly released genomics data have led to the identification of numerous plant gene families, especially in model plants such as *Arabidopsis*. Among novel families, the *CAMTA* gene family has been reported in many plants of agriculture importance^7–10^. However, genome-wide identification and annotation of *CAMTA* genes has not been reported in any of *Nicotiana* species. In this study, 13 *CAMTA* family genes were identified in *N*. *tabacum* genome, which are distributed onto on five chromosomes/scaffolds and clustered into four phylogenetic groups. In addition, we identified six CAMTA genes in *N*. *sylvestris*, and five genes in *N*. *tomentosiformis* and *N*. *benthaminana*, the wild tobacco. The number of genes in *NtabCAMTA* family are greater than *CAMTA* genes in most of the reported crops, such as *A*. *thaliana* (6), *Oryza sativa* (5), *Solanum lycopersicum* (7), *Brassica campestris* (8), *Vitis vinifera* (10), thus making it the largest *CAMTA* family in plants so far. This is probably due to the larger genome size of *N*. *tabacum* of about ~4500 Mb. The *NtabCAMTA*-encoded proteins are characterized by presence of CG-1, ANK, IQ and/or IPT/TIG domains, which are evolutionarily conserved and characteristic to plant CAMTAs. It is assumed that homologous genes within the same taxonomic/phylogenetic group exhibit similar structural, functional and evolutionary properties, which might help in understanding the role of *CAMTA* genes in *N*. *tabacum*. Here we found that *NtabCAMTA*-encoded proteins showed high similarity with corresponding NsylCAMTAs, NtomCAMTAs, NbenCAMTAs and AtCAMTAs in terms of domain architectures, amino acid composition and phylogenies. In addition, these proteins share homology with the corresponding orthologues identified in other plant species. As mentioned earlier, *N*. *tabacum* is an allotetraploid crossbreed originated from the presumptive parental diploids *N*. *sylvestris* and *N*. *tomentosiformis*^39^. We confirmed that most of the *NtabCAMTA* family genes were descended from parental species, and gene duplication significantly contributed to the expansion of this family^40^. Our results are corroborated by the findings of Xu *et al*.^41^, stating that gene duplication increases the genome content and expands gene function to guarantee optimum adaptability and evolution of plants. Meanwhile, two genes (Ntab0695330 and Ntab0966680) were found to lack functional domains in their sequences, which are probably lost during the long course of evolution. These results are supported by the findings of Liang *et al*.^42^, stating that functionally redundant gene copies are often lost during duplication, and only active copies of functional genes are retained.

As in other organisms, transcription factors in plant systems are regulated by different types of miRNAs^43^. We observed that two *NtabCAMTA* gene transcripts (i.e., *NtabCAMTA4* and *NtabCAMTA5*) comprised target sites for nta-miR6164a and nta-miR6164b. Former research has shown that these miRNAs are involved in regulation of pathways associated with morphological and metabolic adaptations^44^, hormone^45^ and symbiotic nitrogen fixation^46^. Besides, some of miRNAs, which were predicted with low expectancy score (nta-miR159, nta-miR394, nta-miR395 and miR477) are reported to play important roles in various abiotic and biotic stress responses including: Cd, salt, cold, heat, drought, Fe deficiency, UV-B radiation, hypoxia or oxidative stress, and resistance to powdery mildew infection and *tobacco mosaic virus*^47–57^.

The *CAMTA* transcription factor family play functional role in plant response to several abiotic and biotic stresses, including cold, wounding, drought and pathogens^58^. Infectious diseases such as PVY, CMV and BSD, along with drought cold and Cd stress have been reported to affect the tobacco yield and production each year^59^. The detailed analyses of gene expression data in different tissues and stress conditions further clarified the important role of the different *CAMTAs* in the growth, development and survival of *N*. *tabacum*. The expression patterns of *NtabCAMTAs* were different in different tissues, where most of the genes were expressed in leaf vein and blades. Among them, the transcription level of *NtabCAMTA3* was significantly higher than other genes, especially after topping, showing their importance in wound response. Under abiotic stress, *NtabCAMTA3* and *NtabCAMTA10* had increasing expressions under cold stress, while, others showing differential expression patterns. Our results are consistent with the findings of Doherty *et al*.^60^, who evidenced that CG-1 sequence is important for early cold response in AtCAMTA proteins. We noticed that two of *N*. *benthaminana CAMTA* genes (*NbenCAMTA1* and *NbenCAMTA2*) comprise two CG-1 domains, therefore, it would be interesting to note if extra domains are functional or contribute to the degree of cold stress tolerance in wild tobacco. Additionally, these *NtabCAMTA* family genes showed differential responses to viral pathogens CMY, PVY, and black shank pathogen infection, drought and cadmium stress. Finally, different co-expression networks were constructed, which revealed that some of NtabCAMTA subunits interact with each other under different conditions. Multiple studies have shown that certain members of *CAMTA* family modulate different biotic and abiotic stress responses^61,62^, which corroborate our findings. Further studies are required to clarify the role and mechanism of differentially expressed *NtabCAMTA* genes in plant growth and development, and the regulation of signal transduction and stress resistance related pathways.

## Conclusion

This work is the first inclusive report about genome-wide identification and systematic characterization of *CAMTA* gene family in four *Nicotiana* species with focus on common tobacco. These CAMTA families can be classified into five phylogenetic groups, and Group-IV CAMTAs probably emerged the earliest among all in *Nicotiana*. There are 13 genes in *N*. *tabacum CAMTA* family originating from ancestral genomes of *N*. *sylvestris* and *N*. *tomentosiformis*. Both polyploidization and duplication events played important role in the expansion of *NtabCAMTA* family. The available information from bioinformatics analysis can be used in futuristic studies to identify and characterize the CAMTA proteins in *Nicotiana* species. For example, using stringent consensus motif keys to identify new genes/families in Nicotiana/solanaceous species, construction of protein-protein interaction networks, experimentally validating gene structures and miRNA targets. Furthermore, the expression data of the differentially expressed *CAMTA* genes (such as *NtabCAMTA-3*, *6*, *7*, *10* and *13*) can lay the foundation for investigating their molecular regulatory mechanisms, and breeding new cultivars with improved yield, quality and tolerance to abiotic/biotic stress.

## Methods

### Identification of *CAMTA* gene family in four *Nicotiana* species

The reference genome and proteome sequences of tobacco (*N*. *tabacum*) variety “K326”, *N*. *sylvestris* and *N*. *tomentosiformis* available at http://www.tobaccodb.org/were used for annotation of the candidate *CAMTA* genes. In order to identify these gene families, the DNA and amino acid sequences of six *Arabidopsis CAMTA* family genes were downloaded from TAIR10 (https://www.arabidopsis.org/), and used as queries to perform homology based search in http://www.tobaccodb.org/, using BLASTN and BLASTP programs respectively with default parameters. Similarly, the 6 AtCAMTA protein sequences were used to search against *N*. *benthaminana* genome using TBLASTN at Sol Genomics Network (http://solgenomics.net/). All non-redundant protein sequences of the candidate *CAMTA* genes were retrieved and subjected to domain analysis by domain analysis programs: Simple Modular Architecture Research Tool (SMART) (http://smart.embl-heidelberg.de/) and the Conserved Domains Database (CDD) (http://www.ncbi.nlm.nih.gov/Structure/cdd/wrpsb.cgi), with the default cut off parameters. Sequences containing CG-1 (PF03859), TIG (PF01833), ANK (PF12796) and IQ (PF00612) domains were recognized as CAMTA proteins. The identified *NtabCAMTA*, *NtomCAMTA*, *NsylCAMTA* and *NbenCAMTA* genes were named according to their positions in phylogenetic tree. The analysis also included putative orthologous *CAMTA* genes from other plant species, which were BLASTP searched and downloaded from Phytozome (https://phytozome.jgi.doe.gov/), using *NtabCAMTA1-*encoded protein as query. Moreover, the sequence data of the identified *CAMTA* genes for *N*. *tabacum*, *N*. *sylvestris* and *N*. *tomentosiformis* was deposited at the GenBank (https://www.ncbi.nlm.nih.gov/genbank/), which can be accessed (accession numbers: MF142771-MF142783, and MH119945-MH119955) by the readers to retrieve, confirm and reproduce the analysis.

### Characterization and physicochemical properties

The information regarding the general characteristics of *CAMTA* genes and proteins was obtained from the “http://www.tobaccodb.org/”. Amino acid properties and other physicochemical traits such as charge, molecular weight (g mol^−1^), aliphatic and instability index (II), isoelectric points (pI), grand average of hydropathy (GRAVY) and other properties of a given NtabCAMTA proteins were calculated using the ProtParam tool in the ExPASy web server^5^. The post-translational modifications sites were predicted by using the ScanProsite tool^6^.

### Sequence alignments and phylogenetic analysis

The multiple sequence alignments for the predicted CAMTA proteins were performed using ClustalX 2.0 program with the default settings^63^, and viewed by GeneDoc program^64^. The identified conserved CAMTA-specific domains were manually checked, verified and shaded with DNAMAN software (version 6.0.3.40, Lynnon Corporation). MEGA 6.0 software was used to conduct the evolutionary analyses. Initial tree(s) for the heuristic search were obtained by applying the Neighbor-Joining method to a matrix of pairwise distances estimated using a Jones-Taylor-Thornton (JTT) matrix-based model. Maximum Likelihood method based on the JTT model with bootstrap of 1000 replicates was used to construct the final phylogenetic trees^65,66^. The trees were drawn to scale, with branch lengths measured in the number of substitutions per site.

### Prediction of gene structure, motifs and miRNA target sites

The structures of the *CAMTA* family genes showing exon-intron organization were determined based on alignments of their coding sequences with the corresponding genomic sequences, and a diagram was obtained using Gene Structure Display Server (GSDS 2.0, http://gsds.cbi.pku.edu.cn/). The conserved motifs in the NtabCAMTA proteins were identified in MEME web server^8^, keeping the optimal motif width between 6 and 200, and the maximum number of different motifs as 10^67^. The discovered motifs were annotated with Pfam program (http://pfam.xfam.org/).

For miRNA’s target sites prediction within the *NtabCAMTA* transcripts, the complete sequence information of all known and published miRNAs of the *N*. *tabacum* was obtained from miRBase (http://www.mirbase.org/). The obtained sequences of 164 tobacco miRNAs and *NtabCAMTA* transcripts were used as input to the psRNATarget server (http://plantgrn.noble.org/psRNATarget/) using default settings and threshold.

### Analysis of CAMTA gene expression in tobacco tissues

To explore the expression patterns of *NtabCAMTA* genes in different tobacco tissues, the Illumina RNA-sequencing data of *N*. *tabacum* cultivar: TN90 was downloaded from GenBank archives at https://www.ncbi.nlm.nih.gov/bioproject/PRJNA208209/SRP029183^18^. There were 36.8 M reads of each RNA sample, from which the low quality reads/adapters were removed, and mapped to the tobacco genes. The gene expression data was normalized by FPKM (fragments per kilobase per million)^68^. The resulting FPKM values of *NtabCAMTA* genes were log_2_ transformed, and the heat maps of the hierarchical clustering were generated and visualized using R language program^69^.

### Growth conditions, sample preparation and stress treatments

Tobacco plants were cultivated in Guiyang County, Guizhou Province, China, under normal growth conditions untill three to five fully expanded leaf stage was reached. For drought stress, *N*. *tabacum* cv. hongda seedlings were kept away from water for 2, 4 and 8 days at reduced relative humidity of ~35%. The whole leaf tissues were harvested from control and dehydrated plants after 2, 4 and 8 days respectively.

For cold and cadmium treatments, *N*. *tabacum* cv. hongda seedlings were grown on Murashige and Skoog medium^70^ for three weeks at 25 ± 1 °C with 16 h of light and 8 h of dark. In earlier case the seedlings were exposed to 4 °C of temperature and leaf samples were collected after 1 day, while in later case seedlings were grown on MS medium supplemented with 250 mM of Cd, and the leaf and root samples were harvested.

For viral inoculum preparation. ~0.5 g of systemically infected leaf tissue from CMV/PVY-infected tobacco seedlings were picked and instantly homogenized with kieselguhr in 1 ml water. The viral inoculums were subsequently rub-inoculated onto the top of two leaves of fresh plants having four fully expanded leaves. The mock inoculum was prepared from leaves of the healthy plants and applied in the same way as viral inoculum. Both viral and mock-inoculated plants were sampled after 6 hours and 1 day respectively. The cultivation of BSD pathogen *P*. *nicotianae*, and the infection of HD and RBST cultivars were performed using the method described by Scharte *et al*.^71^ and Essmann *et al*.^72^. After harvesting, each sample was cut into small pieces, followed by immediate storage in liquid nitrogen and subsequent storage at −80 °C until further processing.

### RNA extraction and RT-qPCR analysis

Total RNA extraction was performed using TRIzol^TM^ reagent (TransGen) following the manufacturer’s instructions. First-strand cDNA was reverse-transcribed from total RNA using M-MLV reverse transcriptase (Promega) with oligo (dT) as the primer. PCR was performed in a total volume of 10 μL containing 5 μL of 2× SYBR Premix Ex Taq (TaKaRa), 2 μmol L−1 of each gene-specific primer (Table S9), 0.5 μL of the cDNA sample, 0.2 μL of Rox Reference DyeII (TaKaRa) on an ABI StepOne Real-time PCR instrument (Applied Biosystems). The reactions were carried out using the following program: 95 °C for 1 min, 45 cycles of 95 °C for 15 sec, and 60 °C for 34 s. The tobacco actin gene (LOC107788267) was used as an internal reference. Each experiment was performed with three technical replicates. Finally, the 2^−ΔΔCt^ method^73^ was used to calculate the relative gene expression values, which were subsequently transformed to fold-change and plotted in figures. Student’s t-tests were used to determine significant differences.

### Identification of correlated genes and network construction

To determine the co-expression network for *NtabCAMTA* genes, the FPKM data from in planta, and different stress treatments were collected and used. First, we ranked the correlated genes with Pearson’s correlation coefficient threshold of higher than 0.75. Then, we recalculated the Pearson’s correlation coefficients of the genes and conditions with p-value ≤ 0.05, with the R project (version 3.2.3). Cytoscape (version 2.8.2) software was used to construct co-expression networks between *NtabCAMTA* and co-expression genes.

### Statistical analysis

All the data were subjected to analysis of variance (ANOVA) using computer statistical package (SAS software SAS Institute, Cary, NC). General linear model (GLM) procedure was used to check the significant differences among main treatments. Individual comparisons between mean values were performed by using the least significant differences (LSD) test (p ≤ 0.05). Correlation analysis was performed by CORR procedure and R project.

## Electronic supplementary material

Supplementary Information
