# Author Correction: Evolutionary and expression analysis of CAMTA gene family in *Nicotiana tabacum* yielded insights into their origin, expansion and stress responses

**DOI:** 10.1038/s41598-020-60470-z

**Published:** 2020-03-10

**Authors:** Kaleem U. Kakar, Zarqa Nawaz, Zhouqi Cui, Peijian Cao, Jingjing Jin, Qingyao Shu, Xueliang Ren

**Affiliations:** 1 Molecular Genetics Key Laboratory of China Tobacco, Guizhou Academy of Tobacco Science, Guiyang, 550081 China; 2 State Key Laboratory of Rice Biology, Institution of Crop Science, Zhejiang University, Hangzhou, 310058 China; 3 Department of Microbiology, Faculty of Life Sciences & Informatics, Balochistan University of Information Technology, Engineering, and Management Sciences, Quetta, 87300 Pakistan; 4 Department of Plant Pathology and Ecology, The Connecticut Agricultural Experimental Station, New haven, CT 06511 USA; 5 Department of Biological Sciences, University of Wisconsin, Milwaukee, WI 53211 USA; 6 China Tobacco Gene Research Center, Zhengzhou Tobacco Research Institute of CNTC, Zhengzhou, 450001 China

Correction to: *Scientific Reports* 10.1038/s41598-018-28148-9, published online 09 July 2018

In the original version of this Article, Kaleem U. Kakar was incorrectly affiliated with ‘Faculty of Life Sciences, University of Balochistan, Quetta, 87300, Pakistan’. The correct affiliations are listed below.

Molecular Genetics Key Laboratory of China Tobacco, Guizhou Academy of Tobacco Science, Guiyang, 550081, China

State Key Laboratory of Rice Biology, Institution of Crop Science, Zhejiang University, Hangzhou, 310058, China

Department of Microbiology, Faculty of Life Sciences & Informatics, Balochistan University of Information Technology, Engineering, and Management Sciences, Quetta, 87300, Pakistan

This error has now been corrected in the PDF and HTML versions of the Article.

