# Supplementary material for: Evolutionary and expression analysis of CAMTA gene family in Nicotiana tabacum yielded insights into their origin, expansion and stress responses
Source: Sci Rep. 2018 Jul 9;8:10322. doi: 10.1038/s41598-018-28148-9 (PMC6037683; doi:10.1038/s41598-018-28148-9)
Supplement: Supplementary file 1 — Supplementary Information [file 41598_2018_28148_MOESM1_ESM.pdf]

Supplementary Material

Evolutionary and expression analysis of *CAMTA* gene family in *Nicotiana tabacum* yielded insights into their origin, expansion and stress responses

Kaleem U. Kakar, Zarqa Nawaz, Zhouqi Cui, Peijian Cao, Jingjing Jin, Qing-Yao Shu and Xue-liang Ren*

*** Correspondence:** Corresponding Author: renxuel@126.com

Supplementary Figure S1. Phylogenies of each CAMTA protein family identified from four *Nicotiana* species. Each maximum likelihood (ML) tree was created with MEGA 6.0, under the Jones-Taylor-Thornton (JTT) model. The bootstrap values from 1000 resampling are given at each node.

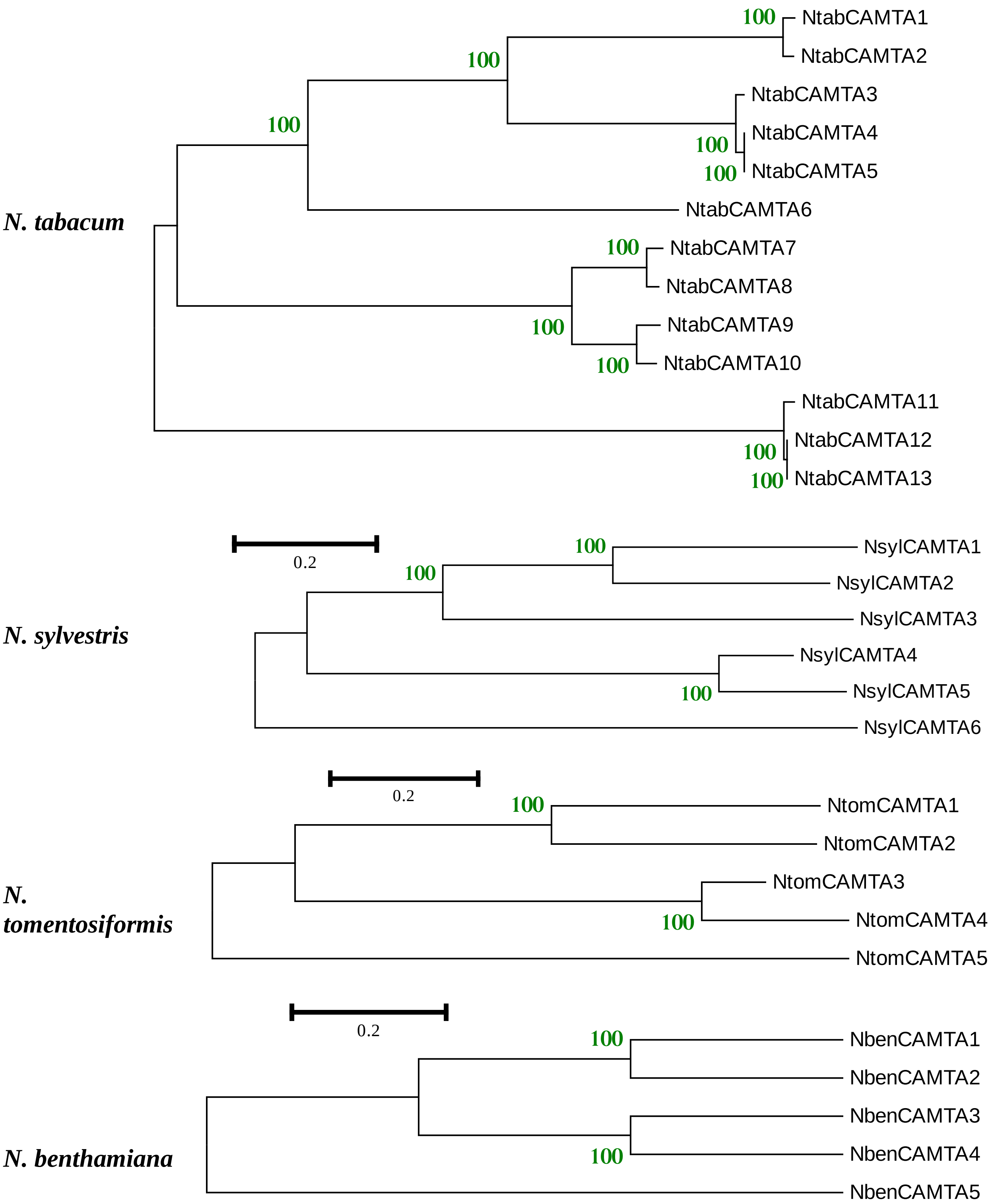

Supplementary Figure S2. Multiple sequence alignment of 29 CAMTA proteins identified from four *Nicotiana* species. The names of the identified CAMTA genes are indicated to the left of the alignments. The length of the alignment is shown on the top, while the length of each protein is given on the right. Conserved consensus residues are shown in the bottom. Invariable residues conserved at 100% are highlighted in red, >80% are highlighted in blue and residues conserved at >60% are highlighted in green. The sequences were aligned by MEGA6.0 and viewed by GENEDOC program.

```
                             *        20         *        40
NsylCAMTA1 : ----------------------------------------------- :    -
NsylCAMTA2 : -----------------------MADSRRYGLNAQ------------ :   12
NsylCAMTA3 : ----------------------------------------------- :    -
NsylCAMTA4 : ------------------------------MAESG------------ :    5
NsylCAMTA5 : -----------MTELYNKQPTIAPVSSPNTKTVK------------- :   23
NsylCAMTA6 : -------------MESSRAGQLAGSDIHGFHTLQD------------ :   22
NtabCAMTA1 : ------------------------------MANGGACE--------- :    8
NtabCAMTA2 : ----------------------------------------------- :    -
NtabCAMTA3 : -------------------------------LNA------------- :    3
NtabCAMTA4 : -----------------------MADSRRYGLNAQ------------ :   12
NtabCAMTA5 : ----------------------------------------------- :    -
NtabCAMTA6 : ----------------------------------------------- :    -
NtabCAMTA7 : --------------------------------ISG------------ :    3
NtabCAMTA8 : MQKRASPSIFSPISFLRRARRNSSPSSGNNGSVQGLWQHLPRHPGST :   47
NtabCAMTA9 : --------------------------------VSG------------ :    3
NtabCAMTA1 : -----------MTELYNKQPTIAPVSSPNTKTVKYSRFQVGSGNRIL :   36
NtabCAMTA1 : -------------MESSRAGQLAGSDIHGFRTLQGIISVTLILLVYA :   34
NtabCAMTA1 : -------------MESSRAGQLAGSDIHGFHTLQG------------ :   22
NtabCAMTA1 : -------------MESSRAGQLAGSDIHGFHTLQD------------ :   22
NtomCAMTA1 : -------------------------------MVNK------------ :    4
NtomCAMTA2 : ------------MKFLSCDRALVHKNPTGITLSEKA----------- :   24
NtomCAMTA3 : ------------------------------MAESG------------ :    5
NtomCAMTA4 : ------------------------------MSLVH------------ :    5
NtomCAMTA5 : -------------MESSRAGQLAGSDIHGFRTLQGIISVTLILLVYA :   34
NbenCAMTA1 : -------------------MADCGSDPPGFR---------------- :   12
NbenCAMTA2 : -----------------------MADSRRYGLNAQ------------ :   12
NbenCAMTA3 : ------------------------------MAESG------------ :    5
NbenCAMTA4 : ------------------------------MAESG------------ :    5
NbenCAMTA5 : -------------MESSRAGQLSGSDIHGFHTLQD------------ :   22

                 *        60         *        80         *
NsylCAMTA1 : ----------------------------------DITQILSEVQHRW :   13
NsylCAMTA2 : ---------------------------------LDIDQILLEAQHRW :   26
NsylCAMTA3 : ---------------------------------------MGQFSRKQY :    9
NsylCAMTA4 : ---------------------------------YDINDLVREAQIRW :   19
NsylCAMTA5 : ---------------------------------YSRFQVGSGNRILN :   37
NsylCAMTA6 : ---------------------------------LDIPSIMEEAKMRW :   36
NtabCAMTA1 : ---------------------------------CRLEAVVDGSGSGH :   22
NtabCAMTA2 : ---------------------------------MTVDD--------W :    6
NtabCAMTA3 : ----------------------------------DIDQILLEAQHRW :   16
NtabCAMTA4 : ---------------------------------LDIDQILLEAQHRW :   26
NtabCAMTA5 : ----------------------------------DIDQILLEAQHRW :   13
NtabCAMTA6 : ----------------------------------------MADTRRY :    7
NtabCAMTA7 : ---------------------------------YDINDLVREAQIRW :   17
NtabCAMTA8 : PR-------------------------------YDINDLVREAQIRW :   63
NtabCAMTA9 : ---------------------------------YNINDLVREGHFRW :   17
NtabCAMTA1 : NERGRERAILHHREILLLRISMAESG-------YNINNLVREGRFRW :   76
NtabCAMTA1 : EMLGCVLVTKEQFHCNLGVNSESD---------LDIPSIMEEAKMRW :   72
NtabCAMTA1 : ----------------------------------------------- :    -
NtabCAMTA1 : ---------------------------------LDIPSIMEEAKMRW :   36
NtomCAMTA1 : ---------------------------------VDITQILSEVQHRW :   18
NtomCAMTA2 : ---------------------------------LDIDQILLEAQHRW :   38
NtomCAMTA3 : ---------------------------------YDINDLVREAQIRW :   19
NtomCAMTA4 : ---------------------------------HKKQDL-SLSLSKW :   18
NtomCAMTA5 : EMLGCVLVTKEQFHCNLGVNSESGTKKTKVGSYLDIPSIMEEAKMRW :   81
NbenCAMTA1 : ---------------------------------LDITQILSEVQHRW :   26
NbenCAMTA2 : ---------------------------------LDIDQILLEAQHRW :   26
NbenCAMTA3 : ---------------------------------YDINDLVREAQIRW :   19
NbenCAMTA4 : ---------------------------------YNINNLVREGRFRW :   19
NbenCAMTA5 : ---------------------------------LDIPSIMEEAKMRW :   36
                                                            rw
```

```
                    100         *       120         *       140
NsylCAMTA1 : LRPAEICEILRNYRKFHITPEAPH----RP----------------- :  39
NsylCAMTA2 : LRPAEICEILKNYQKFRIAPEPPN----RP----------------- :  52
NsylCAMTA3 : --------------------GEAE----QN----------------- :  15
NsylCAMTA4 : LKPAEVLFILRNHEHHQLSNEPAQ----KP----------------- :  45
NsylCAMTA5 : ERGRE-RAILHHREILLLRISMAE----------------------- :  60
NsylCAMTA6 : LRPNEIHAILCNYKYFNIFVKPVN----LPMSNTTLSDYLLWSLSCD :  79
NtabCAMTA1 : FYKRKLSGSLVLMGILKVF---------------------------- :  41
NtabCAMTA2 : ----------------------------------------------- :   -
NtabCAMTA3 : LRPAEICEILKNYQKFRIAPEPPN----RP----------------- :  42
NtabCAMTA4 : LRPAEICEILKNYQKFRIAPEPPN----RP----------------- :  52
NtabCAMTA5 : LRPAEICEILKNYQKFRIAPEPPN----RP----------------- :  39
NtabCAMTA6 : LSN-------------------------QP----------------- :  12
NtabCAMTA7 : LKPAEVLFILRNHEYHQLSNEPAQ----KP----------------- :  43
NtabCAMTA8 : LKPAEVLFILRNHENHQLSNEAAQ----KP----------------- :  89
NtabCAMTA9 : LRPAEVVFILQNHEDQQLANQPPQ----KP----------------- :  43
NtabCAMTA1 : LRPAEVLFILQNHEDQQLANQPPQ----KP----------------- : 102
NtabCAMTA1 : LRPNEIHAILCNYKYFNIFVKPVN----LP----------------- :  98
NtabCAMTA1 : ----------------------------------------------- :   -
NtabCAMTA1 : LRPNEIHAILCNYKYFNIFVKPVN----LPMSNTTLSDYLLWSLSCD :  79
NtomCAMTA1 : LRPAEICEILRNYKKFHITPEAPH----RP----------------- :  44
NtomCAMTA2 : LRPAEICEILKNYQKFRIAPEPPN----RP----------------- :  64
NtomCAMTA3 : LKPAEVLFILRNHENHQLSNEAAQ----KP----------------- :  45
NtomCAMTA4 : SINDGAIF-------QALFIIPLLCLNFQQ----------------- :  41
NtomCAMTA5 : LRPNEIHAILCNYKYFNIFVKPVN----LP----------------- : 107
NbenCAMTA1 : LRPAEICEILRNYRKFHITPEAPH----RP----------------- :  52
NbenCAMTA2 : LRPAEICEILKNYQKFRIAPEPPN----RP----------------- :  52
NbenCAMTA3 : LKPAEVLFILRNHEHHQLSNEPAQ----KP----------------- :  45
NbenCAMTA4 : LRPAEVLFILQNHEDQQLANQPPQ----KP----------------- :  45
NbenCAMTA5 : LRPNEIHAILCNYKYFNIFVKPVN----LP----------------- :  62
             l   e   il                   p

                     *       160        *       180
NsylCAMTA1 : --------------------VSGSVFLFDRKVLRYFRKDGHNWRKKK :  66
NsylCAMTA2 : --------------------PSGSLFLFDRKVLRYFRKDGHSWRKKK :  79
NsylCAMTA3 : --------------------YGGSLFLFDRKVLRYFRKDGHQWRKKK :  42
NsylCAMTA4 : --------------------PSGSLFLFNKRVLRFFRKDGHSWRKKK :  72
NsylCAMTA5 : --------------------SGGSMFLFNKRVLRYFRKDGHSWRKKK :  87
NsylCAMTA6 : DFFLVLCSVILNEMEHDFSKQSGTIVLFDRKMLRNFRKDGHNWKKKK : 126
NtabCAMTA1 : --------------------ACGSVFLFDRKVLRYFRKDGHNWRKKK :  68
NtabCAMTA2 : --------------------SSGSVFLFDRKVLRYFRKDGHNWRKKK :  33
NtabCAMTA3 : --------------------PSGSLFLFDRKVLRYFRKDGHSWRKKK :  69
NtabCAMTA4 : --------------------PSGSLFLFDRKVLRYFRKDGHSWRKKK :  79
NtabCAMTA5 : --------------------PSGSLFLFDRKVLRYFRKDGHSWRKKK :  66
NtabCAMTA6 : --------------------LGGSLFLFDRKVLRYFRKDGHQWRKKK :  39
NtabCAMTA7 : --------------------PSGSLFLFNKRVLRFFRKDGHSWRKKK :  70
NtabCAMTA8 : --------------------PSGSLFLFNKRVLRFFRKDGHSWRKKK : 116
NtabCAMTA9 : --------------------ASGSMFLFNKRVLRYFRKDGHSWRKKK :  70
NtabCAMTA1 : --------------------ASGSMFLFNKRVLRYFRKDGHSWRKKK : 129
NtabCAMTA1 : --------------------TSGTIVLFDRKMLRNFRKDGHNWKKKK : 125
NtabCAMTA1 : ----------------------GTIVLFDRKMLRNFRKDGHNWKKKK :  47
NtabCAMTA1 : DFFLVLCSVILNEMEHDFSKQSGTIVLFDRKMLRNFRKDGHNWKKKK : 126
NtomCAMTA1 : --------------------VSGSVFLFDRKVLRYFRKDGHNWRKKK :  71
NtomCAMTA2 : --------------------PSGSLFLFDRKVLRYFRKDGHSWRKKK :  91
NtomCAMTA3 : --------------------PSGSLFLFNKRVLRFFRKDGHSWRKKK :  72
NtomCAMTA4 : --------------------KSGSMFLFNKRVLRYFRKDGHSWRKKK :  68
NtomCAMTA5 : --------------------TSGTIVLFDRKMLRNFRKDGHNWKKKK : 134
NbenCAMTA1 : --------------------VSGSVFLFDRKVLRYFRKDGHNWRKKK :  79
NbenCAMTA2 : --------------------PSGSLFLFDRKVLRYFRKDGHSWRKKK :  79
NbenCAMTA3 : --------------------PSGSLFLFNKRVLRFFRKDGHSWRKKK :  72
NbenCAMTA4 : --------------------ASGSMFLFNKRVLRYFRKDGHSWRKKK :  72
NbenCAMTA5 : --------------------TSGTIVLFDRKMLRNFRKDGHNWKKKK :  89
                                 sGs fLF   vLR FRKDGH WrKKK
```

```
               *       200         *       220         *
NsylCAMTA1 : DGKTVKEAHEKL----------------------------------- :  78
NsylCAMTA2 : DGKTVKEAHERLKEADQKLMFL------------------------- : 101
NsylCAMTA3 : DGKTVKEAHEKL----------------------------------- :  54
NsylCAMTA4 : DGRTVGEAHERL----------------------------------- :  84
NsylCAMTA5 : DGRTVGEAHERL----------------------------------- :  99
NsylCAMTA6 : DGKTVKEAHEHL----------------------------------- : 138
NtabCAMTA1 : DGKTVKEAHEKLKVSVDSLGIS------------------------- :  90
NtabCAMTA2 : DGKTVKEAHEKL----------------------------------- :  45
NtabCAMTA3 : DGKTVKEAHERLKEADQKLMFL------------------------- :  91
NtabCAMTA4 : DGKTVKEAHERL----------------------------------- :  91
NtabCAMTA5 : DGKTVKEAHERL----------------------------------- :  78
NtabCAMTA6 : DGKTVKEAHEKL----------------------------------- :  51
NtabCAMTA7 : DGRTVGEAHERL----------------------------------- :  82
NtabCAMTA8 : DGRTVGEAHERL----------------------------------- : 128
NtabCAMTA9 : DGRTVGEAHERL----------------------------------- :  82
NtabCAMTA1 : DGRTVGEAHERL----------------------------------- : 141
NtabCAMTA1 : DGKTVKEAHEHL----------------------------------- : 137
NtabCAMTA1 : DGKTVKEAHEHL----------------------------------- :  59
NtabCAMTA1 : DGKTVKEAHEHL----------------------------------- : 138
NtomCAMTA1 : DGKTVKEAHEKL----------------------------------- :  83
NtomCAMTA2 : DGKTVKEAHERL----------------------------------- : 103
NtomCAMTA3 : DGRTVGEAHERL----------------------------------- :  84
NtomCAMTA4 : DGRTVGEAHERL----------------------------------- :  80
NtomCAMTA5 : DGKTVKEAHEHL----------------------------------- : 146
NbenCAMTA1 : DGKTVKEAHEKLKVGSIDVLHCYYAHGEEDDNFQRRSYWMLEQDLMH : 126
NbenCAMTA2 : DGKTVKEAHERLKAGSIDVLHCYYAHGEENEHFQRRSYWMLEEWFTV : 126
NbenCAMTA3 : DGRTVGEAHERL----------------------------------- :  84
NbenCAMTA4 : DGRTVGEAHERL----------------------------------- :  84
NbenCAMTA5 : DGKTVKEAHEHL----------------------------------- : 101
             DG TV EAHE L

                240         *       260         *       280
NsylCAMTA1 : ---------------------------KVGSIDVLHCYYAHGEEDDN :  98
NsylCAMTA2 : ---------------------------QAGSIDVLHCYYAHGEENEN : 121
NsylCAMTA3 : ---------------------------KAGSVDVLHCYYAHGENNEN :  74
NsylCAMTA4 : ---------------------------KVGNAEALNCYYAHGEQNPN : 104
NsylCAMTA5 : ---------------------------KVGNAETLNCYYAHGEKNPN : 119
NsylCAMTA6 : ---------------------------KVGNEERIHVYYAHGEDHPT : 158
NtabCAMTA1 : ---------------------------TVGSIDVLHCYYAHGEEDDN : 110
NtabCAMTA2 : ---------------------------KVGSIDVLHCYYAHGEEDDN :  65
NtabCAMTA3 : ---------------------------QAGSIDVLHCYYAHGEENEN : 111
NtabCAMTA4 : ---------------------------KAGSIDVLHCYYAHGEENEN : 111
NtabCAMTA5 : ---------------------------KAGSIDVLHCYYAHGEENEN :  98
NtabCAMTA6 : ---------------------------KAGSVDVLHCYYAHGENNEN :  71
NtabCAMTA7 : ---------------------------KVGNAEALNCYYAHGEQNPN : 102
NtabCAMTA8 : ---------------------------KVGNAEALNCYYAHGEQNPT : 148
NtabCAMTA9 : ---------------------------KVGNAEALNCYYAHGEKNPN : 102
NtabCAMTA1 : ---------------------------KVGNAETLNCYYAHGEKNPN : 161
NtabCAMTA1 : ---------------------------KVGNEERIHVYYAHGEDHPT : 157
NtabCAMTA1 : ---------------------------KVGNEERIHVYYAHGEDHPT :  79
NtabCAMTA1 : ---------------------------KVGNEERIHVYYAHGEDHPT : 158
NtomCAMTA1 : ---------------------------KVGSIDVLHCYYAHGEEDDN : 103
NtomCAMTA2 : ---------------------------KAGSIDVLHCYYAHGEENEN : 123
NtomCAMTA3 : ---------------------------KVGNAEALNCYYAHGEQNPT : 104
NtomCAMTA4 : ---------------------------KVGNAEALNCYYAHGEKNPN : 100
NtomCAMTA5 : ---------------------------KVGNEERIHVYYAHGEDHPT : 166
NbenCAMTA1 : IVFVH------------------YLEVKVGSIDVLHCYYAHGEEDDN : 155
NbenCAMTA2 : LRYFRKDGHSWRKKKDGKTVKEAHERLKAGSIDVLHCYYAHGEENEH : 173
NbenCAMTA3 : ---------------------------KVGNAEALNCYYAHGEQNPN : 104
NbenCAMTA4 : ---------------------------KVGNAEALNCYYAHGEKNPN : 104
NbenCAMTA5 : ---------------------------KVGNEERIHVYYAHGEDHPT : 121
                                        k G    l cYYAHGE
```

```
                            *      300        *      320
NsylCAMTA1 : FQRRSYWMLEQDLMHIVFVHYLEVKGNKANVGCVRSIKSA------- :  138
NsylCAMTA2 : FQRRSYWMLEEEMSHIVLVHYREVKGNRTNFSRTREPQEATPRFQET :  168
NsylCAMTA3 : FQRRSYWMLEEKLEHIVLVHYREVIESYRVGASRLQP---------- :  111
NsylCAMTA4 : FQRRSYWMLDPVYEHIVLVHYRDITEGRQNPAFM------------- :  138
NsylCAMTA5 : FQRRSYWMLDPAYEHIVLVHYRDITEGMQIAAFM------------- :  153
NsylCAMTA6 : FVRRCYWLLDKSLEHIVLVHYRETQE--------------------- :  184
NtabCAMTA1 : FQRRSYWMLEQDLMHIVFVHYLEVKGNKANVGCVRSIKSA------- :  150
NtabCAMTA2 : FQRRSYWMLEQDLMHIVFVHYLEVKGNKANMGCVRSIKSA------- :  105
NtabCAMTA3 : FQRRSYWMLEEEMSHIVLVHYREVKGNRTNFSRTREPQEATPRFQET :  158
NtabCAMTA4 : FQRRSYWMLEEEMSHIVLVHYREVKGNRTNFSRTREPQEAAPRFQET :  158
NtabCAMTA5 : FQRRSYWMLEEEMSHIVLVHYREVKGNRTNFSRTREPQEAAPRFQET :  145
NtabCAMTA6 : FQRRSYWMLEEKLEHIVLVHYREVIESYRVGASRLQP---------- :  108
NtabCAMTA7 : FQRRSYWMLDPVYEHIVLVHYRDITEGRQNPAFM------------- :  136
NtabCAMTA8 : FQRRSYWMLDPAYEHIVLVHYRDITEGRQNPAFM------------- :  182
NtabCAMTA9 : FQRRSYWMLDPAYEHIVLVHYRDITEGMQIAAFM------------- :  136
NtabCAMTA1 : FQRRSYWMLDPAYEHIVLVHYRDITEGMQIAAFM------------- :  195
NtabCAMTA1 : FVRRCYWLLDKSLEHIVLVHYRETQEVCFLYLARGEILRAGNLRRKR :  204
NtabCAMTA1 : FVRRCYWLLDKSLEHIVLVHYRETQE--------------------- :  105
NtabCAMTA1 : FVRRCYWLLDKSLEHIVLVHYRETQE--------------------- :  184
NtomCAMTA1 : FQRRSYWMLEQDLMHIVFVHYLEVKGNKANMGCVRSIKSA------- :  143
NtomCAMTA2 : FQRRSYWMLEEEMSHIVLVHYREVKGNRTNFSRTREPQEAAPRFQET :  170
NtomCAMTA3 : FQRRSYWMLDPAYEHIVLVHYRDITEGRQNPAFM------------- :  138
NtomCAMTA4 : FQRRSYWMLDPAYEHIVLVHYRDITEGMQIAAFM------------- :  134
NtomCAMTA5 : FVRRCYWLLDKSLEHIVLVHYRETQE--------------------- :  192
NbenCAMTA1 : FQRRSYWMLEQDLMHIVFVHYLEVKGNKANVGCVRSIKSA------- :  195
NbenCAMTA2 : FQRRSYWMLEEEMSHIVLVHYREVKGTRTNFSRTREPQEATPRSQET :  220
NbenCAMTA3 : FQRRSYWMLDPVYEHIVLVHYRDITEGRQNPAFM------------- :  138
NbenCAMTA4 : FQRRSYWMLDPAYEHIVLVHYRDITE---IAAFM------------- :  135
NbenCAMTA5 : FVRRCYWLLDKSLEHIVLVHYRETQE--------------------- :  147
             FqRRsYWmL     HIVlVHYr

                *      340        *      360        *
NsylCAMTA1 : ----HSNY------------------------LNDCSLSDSFPRSLK :  157
NsylCAMTA2 : DEDVHSS-------------------------EVDSSASTKFYPNGY :  190
NsylCAMTA3 : --------------------------------IHPGQLLENPSSSPC :  126
NsylCAMTA4 : --------------------------------SESSPISSTFSPSPS :  153
NsylCAMTA5 : --------------------------------SQSSPISSIFSLSPS :  168
NsylCAMTA6 : --------------------------------AQGSPATSVAKGSP- :  198
NtabCAMTA1 : ----HSNY------------------------LNDCSLSDSFPRSLK :  169
NtabCAMTA2 : ----HSNY------------------------LNDCSLSDSFPRGHK :  124
NtabCAMTA3 : DEDVHSS-------------------------EVDSSASTKFYPNGY :  180
NtabCAMTA4 : DEDVHSS-------------------------EVDSSASTKFYPNDY :  180
NtabCAMTA5 : DEDVHSS-------------------------EVDSSASTKFYPNDY :  167
NtabCAMTA6 : --------------------------------IHPGQLLENPSSSPC :  123
NtabCAMTA7 : --------------------------------SESSPISSTFSPSPS :  151
NtabCAMTA8 : --------------------------------SESSPISSTFSPSPS :  197
NtabCAMTA9 : --------------------------------SQSSPISSTFSLSPS :  151
NtabCAMTA1 : --------------------------------SQSSPISSTFSLSPS :  210
NtabCAMTA1 : ITYVSCCFMCKCFDEDVDHLLLHFQVVITLLVTQGSPVTSVAKGSP- :  250
NtabCAMTA1 : --------------------------------AQGSPATSVAKGSP- :  119
NtabCAMTA1 : --------------------------------AQGSPATSVAKGSP- :  198
NtomCAMTA1 : ----HSNY------------------------LNDCSLSDSFPRGHK :  162
NtomCAMTA2 : DEDVHSS-------------------------EVDSSASTKFYPNDY :  192
NtomCAMTA3 : --------------------------------SESSPISSTFSPSPS :  153
NtomCAMTA4 : --------------------------------SQSSPISSTFSLSPS :  149
NtomCAMTA5 : --------------------------------TQGSPVTSVAKGSP- :  206
NbenCAMTA1 : ----HSNY------------------------LNDCSLSDSFPRSHK :  214
NbenCAMTA2 : DEDVHSS-------------------------EVDSSASTKFYPNDY :  242
NbenCAMTA3 : --------------------------------SESSPISSTFSPSPS :  153
NbenCAMTA4 : --------------------------------SQSSPISSTFSLSPS :  150
NbenCAMTA5 : --------------------------------AQGSPATSVAKGSP- :  161
```

```
                     380         *        400         *        420
NsylCAMTA1 : KLASVNADSTSVASTLTSAHEEAES--------------------ED :  184
NsylCAMTA2 : QVNSQVTDATSLSSAQASEYEDAES--------------------AY :  217
NsylCAMTA3 : FVSGLIVQESHTSSPSSVDWKEQAL----------------------  :  151
NsylCAMTA4 : SYSTQQTGSAVIAGESYEQYQNQFS--------------------PG :  180
NsylCAMTA5 : LYSTQHPGFTVVGSESYQQYQNESS--------------------PG :  195
NsylCAMTA6 : --ATPVNSNSSSDPSDPSGWVLSEE----------------------  :  221
NtabCAMTA1 : KLASVNADSTSVASTLTSAHEEAES---------------GNVVCED :  201
NtabCAMTA2 : KLASANADSTSVASTLTSAHEEAES--------------------ED :  151
NtabCAMTA3 : QVNSQVTDATSLSSAQASEYEDAES--------------------AY :  207
NtabCAMTA4 : QVNSQVTDTTSLSSVQASEYEDAES--------------------AY :  207
NtabCAMTA5 : QVNSQVTDTTSLSSVQASEYEDAES--------------------AY :  194
NtabCAMTA6 : FVSGLIVQESHTSSPSSVDWKEQAL----------------------  :  148
NtabCAMTA7 : SYSTQQTGSAVIAGESYEQYQNQFS--------------------PG :  178
NtabCAMTA8 : SYSTQQTGSTLIAGESYEQYQNQSS--------------------PG :  224
NtabCAMTA9 : LYSTQHPGFTVFGSESYQQYPNESS--------------------PG :  178
NtabCAMTA1 : LYSTQHPGFTVVGSESYQQYQNESS--------------------PG :  237
NtabCAMTA1 : --ATPVNSNSSSDPSDPSGWVLSEE----------------------  :  273
NtabCAMTA1 : --ATPVNSNSSSDPSDPSGWVLSEK----------------------  :  142
NtabCAMTA1 : --ATPVNSNSSSDPSDPSGWVLSEK----------------------  :  221
NtomCAMTA1 : KLASANADSTSVASTLTSAHEEAESVCKCRALNLLQKVSYWKFITED :  209
NtomCAMTA2 : QVNSQVTDTTSLSSVQASEYEDAES--------------------AY :  219
NtomCAMTA3 : SYSTQQTGSTLIAGESYEQYQNQSS--------------------PG :  180
NtomCAMTA4 : LYSTQHPGFTVFGSESYQQYPNESS--------------------PG :  176
NtomCAMTA5 : --ATPVNSNS---SSDPSGWVLSEE----------------------  :  226
NbenCAMTA1 : KLASVNADSTSVASTLTSAHEEAEW--------------------ED :  241
NbenCAMTA2 : QVSSQVTDATSLSSAQASGYEDAES--------------------AY :  269
NbenCAMTA3 : SYSTQQTGSTVIAGESYEQYQNQSS--------------------PG :  180
NbenCAMTA4 : LYSTQHPGFTVIGSESYQQYQNESS--------------------PG :  177
NbenCAMTA5 : --ATPVTSNSSSDPSDPSGWVLSEE----------------------  :  184
```

```
                    *        440         *        460         *
NsylCAMTA1 : SHQACSKFQSYPERASGMDRHLVENRDAIYS--SYGSPQSSVEYTSL :  229
NsylCAMTA2 : NQHPTSGFHSFLDAQPSMMQKAGESLPVPYHPIPFS-NDHQVQFAGS :  263
NsylCAMTA3 : SSELYTGDSKGNEVNPLLVPASGHFLPITSS----------------  :  182
NsylCAMTA4 : --EICSD-AVINNNRTS--DITGRTNEVMSS----------------  :  206
NsylCAMTA5 : SGEICSG-AGINSNGMNISDITGRTEGVSSS----------------  :  225
NsylCAMTA6 : ----CNS-VDERTYGSSQHAHLEPNRDVTAK----------------  :  247
NtabCAMTA1 : SHQACSRFQSYPERASGMDRHLVENRDAIYS--SYGSPQSSVEYTSL :  246
NtabCAMTA2 : SHQACSRFQSYPERASGMDRNLVENRDTIYS--SYGSPQSSVEYTSL :  196
NtabCAMTA3 : NQHPTSGFHSFLDAQPSMMQKAGESLPVPYHPIPFS-NDHQVQFAGS :  253
NtabCAMTA4 : NQHPTSGFHSFLDAQPSMTQKAGEGLAVPYHPIPFS-NDHQVQFAGS :  253
NtabCAMTA5 : NQHPTSGFHSFLDAQPSMTQKAGEGLAVPYHPIPFS-NDHQVQFAGS :  240
NtabCAMTA6 : SSELYTGDSKGNEVNPLLVPASGHFLPITSS----------------  :  179
NtabCAMTA7 : --EICSD-AVINNNRTS--DITGRTNEVMSS----------------  :  204
NtabCAMTA8 : --EICSD-AVINNNGMS--DIIGRTKEVMSS----------------  :  250
NtabCAMTA9 : SGEVCSD-AGINGKGMNISDITGRTEGVSSS----------------  :  208
NtabCAMTA1 : SGEICSG-AGINSNGMNISDITGRTEGVSSS----------------  :  267
NtabCAMTA1 : ----CNS-VDERAYGSSQHAHLEPNRDMTAK----------------  :  299
NtabCAMTA1 : ----CNS-VDERTYGSSQHAHLEPNRDVTAK----------------  :  168
NtabCAMTA1 : ----CNS-VDERTYGSSQHAHLEPNRDVTAK----------------  :  247
NtomCAMTA1 : SHQACSRFQSYPERASGMDRNLVENRDTIYS--SYGSPQSSVEYTSL :  254
NtomCAMTA2 : NQHPTSGFHSFLDAQPSMTQKAGEGLAVPYHPIPFSRDDHQVQFAGS :  266
NtomCAMTA3 : --EICSD-AVINNNGMS--DIIGRTKEVMSS----------------  :  206
NtomCAMTA4 : SGEVCSD-AGINGKGMNISDITGRTEGVSSS----------------  :  206
NtomCAMTA5 : ----CNS-VDERAYGSSQHAHLEPNRDMTAK----------------  :  252
NbenCAMTA1 : SHQACSRFQSYPERASGMDRHLVENRDTIYS--SYGSPQSSVEYTSL :  286
NbenCAMTA2 : NQHPTSGFHSFLDAQPSMMQKAGESLPVPYHPIPFS-NDHQVQFAGS :  315
NbenCAMTA3 : --EICSD-AVINNNRTS--DITGRTNEVMSS----------------  :  206
NbenCAMTA4 : SAEVCSG-AGINSNGMNISDITRRTEGVNIS----------------  :  207
NbenCAMTA5 : ----CNS-VDERTYGSSQHAHLEPNRDVTAK----------------  :  210
```

```
                  520         *        540         *        560
NsylCAMTA1 : --PVSQHCSN-----------------------GEMVCQDDF---- :  274
NsylCAMTA2 : FQPSSQSGANNMTHEQGSTTM-------------GQVF-LNDFKKQG :  343
NsylCAMTA3 : --AQSSD---------------------------------------- :  217
NsylCAMTA4 : --PLYAD---------------------------------------- :  238
NsylCAMTA5 : --PLYSE---------------------------------------- :  252
NsylCAMTA6 : --PDNPN---------------------------------------- :  271
NtabCAMTA1 : --PVSQHCSN-----------------------GEMVCQDDF---- :  291
NtabCAMTA2 : --PVSQHCSN-----------------------GEIVCQDDF---- :  241
NtabCAMTA3 : FQPSSQSGANNMTHEQGSTTM-------------GQVF-LNDFKKQG :  333
NtabCAMTA4 : FQPSSQSGANNMTHEQGNTKT-------------GQVF-LNDFKRQE :  333
NtabCAMTA5 : FQPSSQSGANNMTHEQGNTKT-------------GQVF-LNDFKRQE :  320
NtabCAMTA6 : --AQSSD---------------------------------------- :  214
NtabCAMTA7 : --PLYAD---------------------------------------- :  236
NtabCAMTA8 : --PLYAD---------------------------------------- :  282
NtabCAMTA9 : --PLYSE---------------------------------------- :  240
NtabCAMTA1 : --PLYSE---------------------------------------- :  294
NtabCAMTA1 : --PENPN---------------------------------------- :  323
NtabCAMTA1 : --PDNPN---------------------------------------- :  192
NtabCAMTA1 : --PDNPN---------------------------------------- :  271
NtomCAMTA1 : --PVSQHCSN-----------------------GEIVCQDDF---- :  299
NtomCAMTA2 : FQPSSQSGANNMTHEQGNTKT-------------GQVF-LNDFKRQE :  346
NtomCAMTA3 : --PLYAD---------------------------------------- :  238
NtomCAMTA4 : --PLYSE---------------------------------------- :  238
NtomCAMTA5 : --PDNPN---------------------------------------- :  276
NbenCAMTA1 : --PVSQHCSNGTKSLQSLSCIIFVMNWGLTKYFVGEMVCQDDF---- :  355
NbenCAMTA2 : FQPSSQSGANNMTHEQGSTTT-------------GQVF-LNDFKKQG :  395
NbenCAMTA3 : --PLYAD---------------------------------------- :  238
NbenCAMTA4 : --PLYSE---------------------------------------- :  234
NbenCAMTA5 : --PDNPN---------------------------------------- :  234
                p
```

```
                    *        580         *        600         *
NsylCAMTA1 : -KNNLSVQRNWQYSFGDSA--SQFH-GQIVNQDLIGDSSYDLVNSFH :  317
NsylCAMTA2 : -QNRIDSLGDWQTSEGDAAFISKWSMDQKLNPNLASDHTIRSSAAY- :  388
NsylCAMTA3 : --RNLNVTLQKKFYSGYLNVADLLS-SKLTYARLDGGRAVKDVANSR :  261
NsylCAMTA4 : -------------AISDDS--SLVEMQGSSNSLLLQHHS-AESSESH :  269
NsylCAMTA5 : ----IENSDDVENFGHDNS--SLVQIQHKSNNLLLQPYS-GESSESQ :  292
NsylCAMTA6 : ------KLIATQEAGGRAS-------VGQQNQIEVNGYSLNDGSLSV :  305
NtabCAMTA1 : -KNNLSVQRNWQYSFGDSA--SQFH-GQIVNQDLIGDSSHDLVNSFH :  334
NtabCAMTA2 : -KNNLSVKGNWQYSFGDSA--SQFH-GQIVNQDLIADSSYDLVNSFH :  284
NtabCAMTA3 : -QNRIDSLGDWQTSEGDAAFISKWSMDQKLNPNLASDHTIRSSAAY- :  378
NtabCAMTA4 : RQNRIDGLGDWQTSEGDAAFISKWSMDQKLHPDLASDHTIRSSAAY- :  379
NtabCAMTA5 : RQNRIDGLGDWQTSEGDAAFISKWSMDQKLHPDLASDHTIRSSAAY- :  366
NtabCAMTA6 : --RNLNVTLQKKFYSGYLNVADLLS-SKLTYARLDGGRAVKDVANSR :  258
NtabCAMTA7 : -------------AISDDS--SLVEMQGSSNSLLLQHHS-AESSESH :  267
NtabCAMTA8 : -------------AISDDS--SLVEMQGNSNSLLLQHHS-AESSESH :  313
NtabCAMTA9 : ----IENSDDVENFVHDNN--SLVQIQHKSNNLLLQPHS-GESSESQ :  280
NtabCAMTA1 : ----IENSDDVENFGHDNS--SLVQIQHKSNNLLLQPYS-GESSESQ :  334
NtabCAMTA1 : ------KLNATQEAGGRAS-------AGQQNQFEVNGYSLNDGSLSV :  357
NtabCAMTA1 : ------KLIATQEAGGRAS-------VGQQNQIEVNGYSLNDGSLSV :  226
NtabCAMTA1 : ------KLIATQEAGGRAS-------VGQQNQIEVNGYSLNDGSLSV :  305
NtomCAMTA1 : -KNNLSVKGNWQYSFGDSA--SQFH-GQIVNQDLIADSSYDLVNSFH :  342
NtomCAMTA2 : RQNRIDGLGDWQTSEGDAAFISKWSMDQKLHPDLASDHTIRSSAAF- :  392
NtomCAMTA3 : -------------AISDDS--SLVEMQGNSNSLLLQHHS-AESSESH :  269
NtomCAMTA4 : ----IENSDDVENFVHDNN--SLVQIQHKSNNLLLQPHS-GESSESQ :  278
NtomCAMTA5 : ------KLNATQEAGGRAS-------AGQQNQFEVNGYSLNDGSLSV :  310
NbenCAMTA1 : -KNNLSVQRNWQYSFGDSA--SQFH-GQIVNQDLIGDSSYDLV---- :  394
NbenCAMTA2 : -QNRIDGLGDWQTSEGDAAFISKWSMDQKLNLNLASDHTIRSSAAY- :  440
NbenCAMTA3 : -------------AISDDS--SLVEMQGNSNSLLLQHHS-AESSESH :  269
NbenCAMTA4 : ----VENSDDVENFGHDNS--SLVQIQHKSNNLLLQPHS-GESSESQ :  274
NbenCAMTA5 : ------KLIATQEAGGRAS-------VGQQNQFEVNGYSLNS----- :  263
                                           n  l
```

```
                       620         *        640         *       6
NsylCAMTA1 : NKNLSSDLYTGRGQSYLYPDEQEEQLTQLNIQYLNSLVE---VQGDF :  361
NsylCAMTA2 : ----NVELHNSLEASHILPSHQDKHPMQNELPSQLSDAN---VGGSL :  428
NsylCAMTA3 : NRLTITSGEVLEENIHLAPAQIQNISSSQTVVTPDAAVQNSSLEGRL :  308
NsylCAMTA4 : HQHLTQDGHIWKDMLDHYGVSTADESLNKSLPKLDENGMLQISSERG :  316
NsylCAMTA5 : DQLLNLDGDIWKEMLDHCRSFPAAESQDKCFEKLDENGTLQTLSGMG :  339
NsylCAMTA6 : SRVPVASLESF--VCQVAGSDTVNFNPSNDMPFRSGDGQ---MTSNF :  347
NtabCAMTA1 : NKNLSSDLYTGRGQSYLYPDEQEEQLTQLNIQYLNSLVE---VQGDF :  378
NtabCAMTA2 : NKNLSSDLYTGRGQSYLYPDEQEEQLTQLNIQYLNSLVE---VQGDF :  328
NtabCAMTA3 : ----NVELHNSLEASHILPSHQDKHPMQNELPSQLSDAN---VGGSL :  418
NtabCAMTA4 : ----NVELHNSLEASHILPSHQDKHPMQNELPSQLSDPN---VGGSL :  419
NtabCAMTA5 : ----NVELHNSLEASHILPSHQDKHPMQNELPSQLSDPN---VGGSL :  406
NtabCAMTA6 : NRLTITSGEVLEENIHLAPAQIQNISSSQTVVTPDAAVQNSSLEGRL :  305
NtabCAMTA7 : HQHLTQDGHIWKDMLDHYGVSTADESLNKSLPKLDENGMLQISSERG :  314
NtabCAMTA8 : HQHLTQDGHVWKDMLDHYGVSTAAESLTKSLPKLDENGMLQISSERG :  360
NtabCAMTA9 : HQLLNLDGNIWKEMLDHCRSFPAAESPAKCFEKLDENGTLQTSSGVG :  327
NtabCAMTA1 : HQLLNLDGDIWKEMLDHCRSFPAAESQDKCFEKLDENGTLQTLSGMG :  381
NtabCAMTA1 : SRVPVASLESF--VCQVAGSDTVNFNPSNDTSFRSGDGQ---MTSNF :  399
NtabCAMTA1 : SRVPVASLESF--VCQVAGSDTVNFNPSNDMPFHSGDGQ---MTSNF :  268
NtabCAMTA1 : SRVPVASLESF--VCQVAGSDTVNFNPSNDMPFHSGDGQ---MTSNF :  347
NtomCAMTA1 : NKNLSSDLYTGRGQSYLYPDEQEEQLTQLNIQYLNSLVE---VQGDF :  386
NtomCAMTA2 : ----NVELHNSLEASHILPSHQDKHPMQNELPSQLSDPN---VGGSL :  432
NtomCAMTA3 : HQHLTQDGHVWKDMLDHYGVSTAAESLTKSLPKLDENGMLQISSERG :  316
NtomCAMTA4 : HQLLNLDGNIWKEMLDHCRSFPAAESPAKCFEKLDENGTLQTSSGVG :  325
NtomCAMTA5 : SRVPVASLESF--VCQVAGSDTVNFNPSNDTSFRSGDGQ---MTSNF :  352
NbenCAMTA1 : NKNLSSDLYTGRGQSYLYPDAQEEQLTQLNIQYLNSLVE---VQGDF :  438
NbenCAMTA2 : ----NVELHNSLEASHILPSHQEKHPMQNELPSQHSDAN---VGGSL :  480
NbenCAMTA3 : HQHLAQDGHIWKDMLDHYGVSTAAESLNKYLPKLDENGMLQISSERG :  316
NbenCAMTA4 : HQLLNLDGDIWKEMLDHSRSFLAAESQDKCFEKLDEN---------- :  311
NbenCAMTA5 : ----------------------------------------------- :    -

             60         *         680         *       700
NsylCAMTA1 : NQENSMDM-LGL-GDYYTIKQPHLNSVKMEEGLKKVDSFSRWV-VKE :  405
NsylCAMTA2 : NAELDHNLSIGVRTDHSSLKQPLLDGV-LREGLKKLDSFDRWM-SKE :  473
NsylCAMTA3 : NSDEAGS-------------------------LKKLDSFGRWMDREI :  330
NsylCAMTA4 : AIEAYQSYKWPNFSEKEAQKAPI-------PAFKQLENFKYPAYSPG :  356
NsylCAMTA5 : PIEVTESDRWLKFGGKEALKSSL-------TNFKQVEDFKYPA-CAR :  378
NsylCAMTA6 : RKNEPGVTTVGAGDSFDSLNK---------DGLQTQDSFGRWI---- :  381
NtabCAMTA1 : NQENSMDM-LGL-GDYYTIKQPHLNSVKMEEGLKKVDSFSRWV-VKE :  422
NtabCAMTA2 : NQENSMDM-LGL-GDYSTIKHPHLNSVKMEEGLKKVDSFSRWV-VKE :  372
NtabCAMTA3 : NAELDHNLSIGVRTDHSSLKQPLLDGV-LREGLKKLDSFDRWM-SKE :  463
NtabCAMTA4 : NADLDHNLSIGVRTDHSSLKQPLLDGV-LREGLKKLDSFDRWM-SKE :  464
NtabCAMTA5 : NADLDHNLSIGVRTDHSSLKQPLLDGV-LREGLKKLDSFDRWM-SKE :  451
NtabCAMTA6 : NSDEAGS-------------------------LKKLDSFGRWMDREI :  327
NtabCAMTA7 : AIEAYQSYKWPNFSEKEAQKAPI-------PAFKQLENFKYPAYSPG :  354
NtabCAMTA8 : AIEAYQSYKWPNFSEKEAQKAPI-------PAFKQLENFKYPAYSPG :  400
NtabCAMTA9 : PIEATESDRWLKFGGK-ALKSSL-------TNFKQVEDFKYPA-CAR :  365
NtabCAMTA1 : PIEVTESDRWLKFGGKEALKSSL-------TNFKQVEDFKYPA-CAR :  420
NtabCAMTA1 : QKNESGVTTVGAGDSFDSLNK---------DGLQTQDSFGRWI---- :  433
NtabCAMTA1 : RKNEPGVTTVGAGDSFDSLNK---------DGLQTQDSFGRWI---- :  302
NtabCAMTA1 : RKNEPGVTTVGAGDSFDSLNK---------DGLQTQDSFGRWI---- :  381
NtomCAMTA1 : NQENSMDM-LGL-GDYSTIKHPHLNSVKMEEGLKKVDSFSRWV-VKE :  430
NtomCAMTA2 : NADLDHNLSIGVRTDHSSLKQPLLDGV-LREGLKKLDSFDRWM-SKE :  477
NtomCAMTA3 : AIEAYQSYKWPNFSEKEAQKAPI-------PAFKQLENFKYPAYSPG :  356
NtomCAMTA4 : PIEATESDRWLKFGGK-ALKSSL-------TNFKQVEDFKYPA-CAR :  363
NtomCAMTA5 : QKNESGVTTVGAGDSFDSLNK---------DGLQTQDSFGRWI---- :  386
NbenCAMTA1 : NQENSMDM-LGL-GDYSTIKQPHLNSVKMEEGLKKVDSFSRWV-VKE :  482
NbenCAMTA2 : NAELDHNLSIGVRTDHSSLKQPLLDGV-LREGLKKLDSFDRWM-SKE :  525
NbenCAMTA3 : AIEAYQSYKWPNFSETEAQKATI-------PAFKPLEDFKYSSYCPG :  356
NbenCAMTA4 : --------------------------------QVEDFKYPA-CAR :  323
NbenCAMTA5 : ----------------------------------------------- :    -
                                               f
```

```
                    *        720          *        740          *
NsylCAMTA1 : LEDVEELHMQPTNRISWNVIDTLD--DGSCLPTQLHVDSDSLNPSLS : 450
NsylCAMTA2 : LEDVSEPHMQSNSSSYWDNVGDDDGVDNSTIASQVQLDTYMLSPSLS : 520
NsylCAMTA3 : AVDGNESLLASDSGNYWNTLDNGDK-EVARLSCHMQLDTNSLGPFLS : 376
NsylCAMTA4 : V-----TAFGSNSDQCTTIFDQDQ--IGTSLEDEMSL-------TIS : 389
NsylCAMTA5 : I-----NTYGSYSDQYTTIFDQDL--IGTSFEDDMSL-------TIA : 411
NsylCAMTA6 : ------NYFISDSPGSADEMMTPE--SSVTIDQSYVM---------- : 410
NtabCAMTA1 : LEDVEELHMQPTNRISWNVIDTLD--DGSCLPTQLHVDSDSLNPSLS : 467
NtabCAMTA2 : LEDVEELHMQRTNRISWNVIDTED--DGSCLPTQLHVDSDSLNPSLS : 417
NtabCAMTA3 : LEDVSEPHMQSNSSSYWDNVGDDDGVDNSTIASQVQLDTYMLSPSLS : 510
NtabCAMTA4 : LEDVSEPHMQSNSSSYWDNVGDDDGVDNSTIASQVQLDTYMLSPSLS : 511
NtabCAMTA5 : LEDVSEPHMQSNSSSYWDNVGDDDGVDNSTIASQVQLDTYMLSPSLS : 498
NtabCAMTA6 : AVDGNESLLASDSGNYWNTLDNGDK-EVARLSCHMQLDTNSLGPFLS : 373
NtabCAMTA7 : V-----TAFGSNSDQCTTIFDQDQ--IGTSLEDEMSL-------TIS : 387
NtabCAMTA8 : V-----TAFGSNSDQCTTIFDQDQ--IGTSFEDEMSL-------TIS : 433
NtabCAMTA9 : I-----NTYGSYSDQYTTIFDQDQ--IGTSFEDDMSL-------TIA : 398
NtabCAMTA1 : I-----NTYGSYSDQYTTIFDQDL--IGTSFEDDMSL-------TIA : 453
NtabCAMTA1 : ------NYFISDSPGSADEMMTPE--SSVTIDQSYVM---------- : 462
NtabCAMTA1 : ------NYFISDSPGSADEMMTPE--SSVTIDQSYVM---------- : 331
NtabCAMTA1 : ------NYFISDSPGSADEMMTPE--SSVTIDQSYVM---------- : 410
NtomCAMTA1 : LEDVEELHMQPTNRISWNVIDTED--DGSCLPTQLHVDSDSLNPSLS : 475
NtomCAMTA2 : LEDVSEPHMQSNSSSYWDNVGDDDGVDNSTIASQVQLDTYMLSPSLS : 524
NtomCAMTA3 : V-----TAFGSNSDQCTTIFDQDQ--IGTSFEDEMSL-------TIS : 389
NtomCAMTA4 : I-----NTYGSYSDQYTTIFDQDQ--IGTSFEDDMSL-------TIA : 396
NtomCAMTA5 : ------NYFISDSPGSADEMMTPE--SSVTIDQSYVM---------- : 415
NbenCAMTA1 : LENVEELHMQPTNRISWNVIDTEN--DGSCLPTQLHVDSDSLNPSLS : 527
NbenCAMTA2 : LEDVSEPHMQSNSSSYWDNVGDDEGVDNSTIASQVQLDTYMLSPSLS : 572
NbenCAMTA3 : V-----TAFGSNSDQCTTIFDQDQ--IGTSLEDEMSL-------TIS : 389
NbenCAMTA4 : I-----NTYGSYSDQYTTIFDQDQ--IGTSFEDDMRL-------IIA : 356
NbenCAMTA5 : ----------------------------------------------- :   -
                       s s
```

```
                  760         *          780          *         80
NsylCAMTA1 : QEQVFSIIDFSPNWAYSNLETK------------------------- : 472
NsylCAMTA2 : QDQFFSIIDFSPSWAFAGSEIK------------------------- : 542
NsylCAMTA3 : QEQLFSISDFAPDWAYSGVETKVNLPDLIEWSVLIRNFENPRRHIVL : 423
NsylCAMTA4 : QKQKFTIRDISPDWGYSSEATK------------------------- : 411
NsylCAMTA5 : QKQKFTFHDISPDWGYSSEATK------------------------- : 433
NsylCAMTA6 : -QQIFNITEISPTWALSSEETK------------------------- : 431
NtabCAMTA1 : QEQVFSIIDFSPNWAYSNLETK------------------------- : 489
NtabCAMTA2 : QEQVFSIIDFSPNWAYSNLETK------------------------- : 439
NtabCAMTA3 : QDQFFSIIDFSPSWAFAGSEIK------------------------- : 532
NtabCAMTA4 : QDQFFSIIDFSPSWAFAGSEIK------------------------- : 533
NtabCAMTA5 : QDQFFSIIDFSPSWAFAGSEIK------------------------- : 520
NtabCAMTA6 : QEQLFSISDFAPDWAYSGVETKVNLPDLIEWSVLIRNFENPRRHIVL : 420
NtabCAMTA7 : QKQKFTIRDISPDWGYSSEATK------------------------- : 409
NtabCAMTA8 : QKQKFTFRDISPDWGYSSEATK------------------------- : 455
NtabCAMTA9 : QKQKFTIHDISPDWGYSSEATK------------------------- : 420
NtabCAMTA1 : QKQKFTFHDISPDWGYSSEATK------------------------- : 475
NtabCAMTA1 : -QQIFNITEISPTWALSSEETK------------------------- : 483
NtabCAMTA1 : -QQIFNITEISPTWALSSEETK------------------------- : 352
NtabCAMTA1 : -QQIFNITEISPTWALSSEETK------------------------- : 431
NtomCAMTA1 : QEQVFSIIDFSPNWAYSNLETK------------------------- : 497
NtomCAMTA2 : QDQFFSIIDFSPSWAFAGSEIK------------------------- : 546
NtomCAMTA3 : QKQKFTFRDISPDWGYSSEATK------------------------- : 411
NtomCAMTA4 : QKQKFTIHDISPDWGYSSEATK------------------------- : 418
NtomCAMTA5 : -QQIFNITEISPTWALSSEETK------------------------- : 436
NbenCAMTA1 : QEQVFSIIDFSPNWAYSNLETK------------------------- : 549
NbenCAMTA2 : QDQFFSIIDFSPSWAFAGSEIK------------------------- : 594
NbenCAMTA3 : QKQKFTIRDISPDWGYSSEATK------------------------- : 411
NbenCAMTA4 : QKQKFTFHDISPDWGYSSEATK------------------------- : 378
NbenCAMTA5 : ----------------------------------------------- :   -
             q q f i d sp w        k
```

```
                   0          *        820          *        840
NsylCAMTA1 : ---------------VLITGRFLKSEGELIECKWSCMFGEIEVPAEV :  504
NsylCAMTA2 : ---------------VLITGKFLKSQPEVEK--WACMFGELEVPAEV :  572
NsylCAMTA3 : VIDVKIQINGYISLQVLIIGTFLGHGKHPTSQKWSCMFGEVEVSAEL :  470
NsylCAMTA4 : ---------------VVIIGSFL---CNPSECMWTCMFGDTEVPIQI :  440
NsylCAMTA5 : ---------------VMIVGSFL---CNPSEYTWTCMFGDIEVPVQI :  462
NsylCAMTA6 : ---------------ILVIGHFPGAQSQLAKSNLFCVCADVCFPAEF :  463
NtabCAMTA1 : ---------------VLITGRFLKSEGELIECKWSCMFGEIEVPAEV :  521
NtabCAMTA2 : ---------------VLITGRFLKSEGELIECKWSCMFGEVEVPAEV :  471
NtabCAMTA3 : ---------------VLITGKFLKSQPEVEK--WACMFGELEVPAEV :  562
NtabCAMTA4 : ---------------VLITGKFLKSQPEVEKCSWACMFGELEVPAEV :  565
NtabCAMTA5 : ---------------VLITGKFLKSQPEVEKCSWACMFGELEVPAEV :  552
NtabCAMTA6 : VIDVKIQINGYISLQVLIIGTFLGHGKHPTSQKWSCMFGEVEVSAEL :  467
NtabCAMTA7 : ---------------VVIIGSFL---CNPSECMWTCMFGDTEVPIQI :  438
NtabCAMTA8 : ---------------VVIIGSFL---CNPSECMWTCMFGDSEVPVQI :  484
NtabCAMTA9 : ---------------IVIVGSFL---CNPSEYTWTCMFDDIEVPVQI :  449
NtabCAMTA1 : ---------------VMIVGSFL---CNPSEYTWTCMFGDIEVPVQI :  504
NtabCAMTA1 : ---------------ILVIGHFPGGQSQLAKSNLFCVCADVCFPAEF :  515
NtabCAMTA1 : ---------------ILVIGHFPGAQSQLAKSNLFCVCADVCFPAEF :  384
NtabCAMTA1 : ---------------ILVIGHFPGAQSQLAKSNLFCVCADVCFPAEF :  463
NtomCAMTA1 : ---------------VLITGRFLKSEGELIECKWSCMFGEVEVPAEV :  529
NtomCAMTA2 : ---------------VLITGKFLKSQPEVEKCSWACMFGELEVPAEV :  578
NtomCAMTA3 : ---------------VVIIGSFL---CNPSECMWTCMFGDTEVPVQI :  440
NtomCAMTA4 : ---------------IVIVGSFL---CNPSEYTWTCMFDDIEVPVQI :  447
NtomCAMTA5 : ---------------ILVIGHFPGGQSQLAKSNLFCVCADVCFPAEF :  468
NbenCAMTA1 : ---------------VLITGRFLKSEGELIECKWSCMFGEIEVPAEV :  581
NbenCAMTA2 : ---------------VLITGKFLKSQPEVEK--WACMFGELEVPAEV :  624
NbenCAMTA3 : ---------------VVIIGSFL---CNPSECMWTCMFGDTEVPIQI :  440
NbenCAMTA4 : ---------------VIIVGSFL---CNPSEYTWTCMFGDIEVPVQI :  407
NbenCAMTA5 : ----------------------------------------------- :    -
                                  i g fl          w cmf    evp

               *         860         *         880         *
NsylCAMTA1 : LADGVLRCHAPPHKPGVLPFYVTCSNRLACSEVREFEYRL---GAYQ :  548
NsylCAMTA2 : IADGVLRCHTPNQKVGRVPFYITCSNRLACSEVREFEFRV---SESQ :  616
NsylCAMTA3 : LTQSIIRCEVPSHSPGRVPFYVTCSNRLACSEVREFEYR----EKSS :  513
NsylCAMTA4 : IQEGVICCQAPPHLPGKVTLCVTSGNRESCSEVKEFEYRVKPDDCAR :  487
NsylCAMTA5 : IKEGAIRCQAPPHLPGKVTLCVTTGNRVSCSEVREFEYRVKFDDHGQ :  509
NsylCAMTA6 : VQSGVYRCVISPQPPGLVSLYLSFDGNTPISQVMTYEFRA---PSAC :  507
NtabCAMTA1 : LADGVLRCHAPPHKPGVLPFYVTCSNRLACSEVREFEYRL---GAYQ :  565
NtabCAMTA2 : LADGVLRCHAPPHKPGVLPFYVTCSNRLACSEVREFEYRL---GAYQ :  515
NtabCAMTA3 : IADGVLRCHTPNQKVGRVPFYITCSNRLACSEVREFEFRV---SESQ :  606
NtabCAMTA4 : IADGVLRCHTPIQKAGRVPFYITCCNRLACSEVREFEFRV---TEGQ :  609
NtabCAMTA5 : IADGVLRCHTPIQKAGRVPFYITCCNRLACSEVREFEFRV---TEGQ :  596
NtabCAMTA6 : LTQSIIRCEVPSHSPGRVPFYVTCSNRLACSEVREFEYR----EKSS :  510
NtabCAMTA7 : IQEGVICCQAPPHLPGKVTLCVTSGNRESCSEVKEFEYRVKPDDCAR :  485
NtabCAMTA8 : IQEGVICCQAPPHLPGKVTLCVTSGNRESCSEVKEFEYRDKPDDCAR :  531
NtabCAMTA9 : INEGAIRCQAPPHLPCKVTLCVTTGNRVSCSEVWEFEYRVKFDDHGQ :  496
NtabCAMTA1 : IKEGAIRCQAPPHLPAEV----------------------------- :  522
NtabCAMTA1 : VQSGVYRCVISPQPPGLVNLYLSFDGNTPISQVMTYEFRA---PSAR :  559
NtabCAMTA1 : VQSGVYRCVISPQPPGLVSLYLSFDGNTPISQVMTYEFRA---PSAC :  428
NtabCAMTA1 : VQSGVYRCVISPQPPGLVSLYLSFDGNTPISQVMTYEFRA---PSAC :  507
NtomCAMTA1 : LADGVLRCHAPPHKPGVLPFYVTCSNRLACSEVREFEYRL---GAYQ :  573
NtomCAMTA2 : IADGVLRCHTPIQKAGRVPFYITCCNRLACSEVREFEFRV---TEGQ :  622
NtomCAMTA3 : IQEGVICCQAPPHLPGKVTLCVTSGNRESCSEVKEFEYRDKPDDCAR :  487
NtomCAMTA4 : INEGAIRCQAPPHLPCKVTLCVTTGNRVSCSEVWEFEYRVKFDDHGQ :  494
NtomCAMTA5 : VQSGVYRCVISPQPPGLVNLYLSFDGNTPISQVMTYEFRA---PSAR :  512
NbenCAMTA1 : LADGVLRCHAPPHKPGVLPFYVTCSNRLACSEVREFEYRL---GAYQ :  625
NbenCAMTA2 : IADGVLRCHTPNQKAGRVPFYITCSNRLACSEVREFEFRV---SESQ :  668
NbenCAMTA3 : IQEGVICCQAPPRLPGKVTLCVTSGNRESCSEVKEFEYRVKPDDCAR :  487
NbenCAMTA4 : IKEGAICCQAPPHLPGKVTLCVTTGNRLSCSEVREFEYRVKFDGHGQ :  454
NbenCAMTA5 : ---GVYRCVISPQPPGLVSLYLSFDGNTPISQVMTYEFRA---PSAR :  304
                 g  rC  p   pg v             s v   e r
```

```
                         900         *         920         *         940
NsylCAMTA1 : EFG---AANVSATEMHLLE-RIESLLSLEPLSSCHSSDSMEAAKEK- :  590
NsylCAMTA2 : DVDV--ANSCSSSESLLHM-RFGKLLSLE---STVSLSSPPRSEDDV :  657
NsylCAMTA3 : ELA---LALRPSDEVRLQV-RLAKLLYSGLNKKFLDCSSTECERGKL :  556
NsylCAMTA4 : NNRSDIEGAYKSTEELLLLVRFVQMLLLD----------LSVHKEDS :  524
NsylCAMTA5 : NNLAEVGGACKSSEELLHLVRFVQMLLSD----------SSVQKGDG :  546
NsylCAMTA6 : KWTAPLEEQSSWDEFRVQM-RLAHLLFST--SKSLSIFSSKVHQDS- :  550
NtabCAMTA1 : EFG---AANVSATEMHLLE-RIESLLSLEPLSSCHSSDSMEAAKEK- :  607
NtabCAMTA2 : EIG---AANVSATEMHLLE-RIESLMSLGPVSSCHSSDSMEAAKEK- :  557
NtabCAMTA3 : DVDV--ANSCSSSESLLHM-RFGKLLSLE---STVSLSSPPRSEDDV :  647
NtabCAMTA4 : DADVANANSCSSSESLLHM-RFGKLLSLE---STVSLSSPPRSEDDV :  652
NtabCAMTA5 : DADVANANSCSSSESLLHM-RFGKLLSLE---STVSLSSPPRSEDDV :  639
NtabCAMTA6 : ELA---LALRPSDEVRLQV-RLAKLLYSGLNKKFLDCSSTDCERGKL :  553
NtabCAMTA7 : NNRSDIEGAYKSTEELLLLVRFVQMLLLD----------LSVHKEDS :  522
NtabCAMTA8 : NNRSDVEGAYKSTEELLLLVRFVQLLLLD----------LSAQKEDS :  568
NtabCAMTA9 : KNLAEVGGACKSSEELLLLVRFVQMLLSD----------SSVQKGDG :  533
NtabCAMTA1 : -------GGACKSSEELLHLVRFVQMLLSD----------SSVQKGDG :  553
NtabCAMTA1 : KWTAPLEEQSSWDEFRVQM-RLAHLLFST--SKSLSIFSSKVHQDS- :  602
NtabCAMTA1 : KWTAPLEEQSSWDEFRVQM-RLAHLLFST--SKSLSIFSSKVHQDS- :  471
NtabCAMTA1 : KWTAPLEEQSSWDEFRVQM-RLAHLLFST--SKSLSIFSSKVHQDS- :  550
NtomCAMTA1 : EIG---AANVSATEMHLLE-RIESLMSLGPVSSCHSSDSMEAAKEK- :  615
NtomCAMTA2 : DADVANANSCSSSESLLHM-RFGKLLSLE---STVSLSSPPRSEDDV :  665
NtomCAMTA3 : NNRSDVEGAYKSTEELLLLVRFVQLLLLD----------LSAQKEDS :  524
NtomCAMTA4 : KNLAEVGGACKSSEELLLLVRFVQMLLSD----------SSVQKGDG :  531
NtomCAMTA5 : KWTAPLEEQSSWDEFRVQM-RLAHLLFST--SKSLSIFSSKVHQDS- :  555
NbenCAMTA1 : EFG---AANVSASEMHLLE-RIESLLSLEPVSSCHSSDSMEAAKEK- :  667
NbenCAMTA2 : DVDV--ANSCSSSESLLHM-RFGKLLSLE---STVSPSSPPRSEDDV :  709
NbenCAMTA3 : NNRSAVEGAYKSTEELLLLVRFVQMLLLD----------LSVHKEDS :  524
NbenCAMTA4 : NNLAEVGGACKSSEELLHLVRFVQMLLSD----------SSVQKGDG :  491
NbenCAMTA5 : KWTAPLEEQSSWDEFRVQM-RLAHLLFST--SKSLSIFSSRVHQDS- :  347
               [illegible]

                    *         960         *         980
NsylCAMTA1 : QSTVNRII--CMMEEENQQMIERASDHDTSQCGVKEDLFLERKQKQN :  635
NsylCAMTA2 : SNVCSKINSLLKEDDNEWEEMLNLTYENNFMAEKVKDQLLQKLLKEK :  704
NsylCAMTA3 : KTLLCSLKCNIGNASESLEDLLAIIEGNHI---NFRDTLIQSFMKDK :  600
NsylCAMTA4 : SELSNDFLEKSKANEDSWSQVIESLLFGTSTSTITIDWLLQELLKDK :  571
NsylCAMTA5 : SGSSNDILENSKASEDSWSQVIESLLFGTSTSMVTVDWLLQELLKDK :  593
NsylCAMTA6 : LKEAKRFVRKCSHITDNWAYLIKSIEDRKLPVPHAKDCLFELSLQTK :  597
NtabCAMTA1 : QSTVNRII--CMMEEENQQMIERASDHDTSQCGVKEDLFLERKLKQN :  652
NtabCAMTA2 : HSTVNKII--CMMEEENQQMIERASDYDTSQCGVKEDLFLERKLKQN :  602
NtabCAMTA3 : SNVCSKINSLLKEDDNEWEEMLNLTYENNFMAEKVKDQLLQKLLKEK :  694
NtabCAMTA4 : SHVCSKINSLLNEDDNEWEEMLNLTYENNFMAEKVKDQLLQKLLKEK :  699
NtabCAMTA5 : SHVCSKINSLLNEDDNEWEEMLNLTYENNFMAEKVKDQLLQKLLKEK :  686
NtabCAMTA6 : KTLLCSLKCNIGNASESLEDLLAIIEGNHI---NFRDTLIQSFMKDK :  597
NtabCAMTA7 : SELSNDFLEKSKANEDSWSQVIESLLFGTSTSTITIDWLLQELLKDK :  569
NtabCAMTA8 : SMLSNDFLEKCKANEDSWSQVIESLLFGTSTSTITIDWLLQELLKDK :  615
NtabCAMTA9 : SGSSNDILENSKASEDSWSQVIESLLFGTSTSMVTVDWLLQELLKDR :  580
NtabCAMTA1 : SGSSNDILENSKASEDSWSQVIESLLFGTSTSMVTVDWLLQELLKDK :  600
NtabCAMTA1 : LKEAKRFVRKCSHITDNWAYLIKSIEDRKLPVPHAKDCLFELSLQTK :  649
NtabCAMTA1 : LKEAKRFVRKCSHITDNWAYLIKSIEDRKLPVPHAKDCLFELSLQTK :  518
NtabCAMTA1 : LKEAKRFVRKCSHITDNWAYLIKSIEDRKLPVPHAKDCLFELSLQTK :  597
NtomCAMTA1 : HSTVNKII--CMMEEENQQMIERASDYDTSQCGVKEDLFLERKLKQN :  660
NtomCAMTA2 : SHVCSKINSLLKEDDNEWEEMLNLTYENNFMAEKVKDQLLQKLLKEK :  712
NtomCAMTA3 : SMLSNDFLEKCKANEDSWSQVIESLLFGTSTSTITIDWLLQELLKDK :  571
NtomCAMTA4 : SGSSNDILENSKASEDSWSQVIESLLFGTSTSMVTVDWLLQELLKDR :  578
NtomCAMTA5 : LKEAKRFVRKCSHITDNWAYLIKSIEDRKLPVSHAKDCLFELSLQTK :  602
NbenCAMTA1 : QNTVNRII--CMMEEENQQMIERVSDHDTSQCGVKEDLFLERKLKQN :  712
NbenCAMTA2 : SHVCSKINSLLKEDDNEWEEMLNLTYENNFMAEKVKDQLLQKLLKEK :  756
NbenCAMTA3 : SELSNDLLEKSKANEDSWSQVIESLLFGTSTSTITIDWLLEELLKDK :  571
NbenCAMTA4 : SGSSNDILENSKASQDSWSQVIESLLFGTSTSMVTVDWLLQELLKDK :  538
NbenCAMTA5 : LKEAKRFVRKCSHITDNWAYLIKSIEDRKLPVPHAKDCLFELSLQTK :  394
                                                    D l      lk
```

```
                    *       1000         *       1020          *
NsylCAMTA1 : FYAWLVRQVT-DDGRGRTAIDDEGQGVLHLAAALGYDWALKPILASG : 681
NsylCAMTA2 : LRVWLLQKVA-EGGKGPNVLDEGGQGVLHFAAALGYDWAIPPTIAAG : 750
NsylCAMTA3 : FYEWLVSRAH-EEDKGPNILDDEGQGVIHLVASLGYEWGLVLLTAAG : 646
NsylCAMTA4 : FQQWLSYKLQRKDNQMGCSLSKKEQGIIHMVSGLGFEWALHPILNAG : 618
NsylCAMTA5 : LQQWLSSKLQVQNNQMGYSFSRKEQGIIHMVAVLGFEWALQPILDAG : 640
NsylCAMTA6 : FHEWLLERVI--GGCKTSEWDEQGQGVIHLCAILGYTWAVYPFSWSG : 642
NtabCAMTA1 : FYAWLVRQVT-DDGRGRTAIDDEGQGVLHLAAALGYDWALKPILASG : 698
NtabCAMTA2 : FYAWLVRQVT-DDGRGRTAIDDEGQGVLHLAAALGYDWALKPILASG : 648
NtabCAMTA3 : LRVWLLQKVA-EGGKGPNVLDEGGQGVLHFAAALGYDWAIPPTIAAG : 740
NtabCAMTA4 : LRVWLLQKVA-EGGKGPNVLDEGGQGVLHFAAALGYDWAIPPTIAAG : 745
NtabCAMTA5 : LRVWLLQKVA-EGGKGPNVLDEGGQGVLHFAAALGYDWAIPPTIAAG : 732
NtabCAMTA6 : FYEWLVSRAH-EEDKGPNILDDEGQGVIHLVASLGYEWGLVLLTAAG : 643
NtabCAMTA7 : FQQWLSYKLQRKDNQMGCSLSKKEQGIIHMVSGLGFEWALHPILNAG : 616
NtabCAMTA8 : FQQWLSYKLQRKDNQMGCSLSKKEQGIIHMVSGLGFEWALHPILNAA : 662
NtabCAMTA9 : LKQWLSSKLQVKNNQMGYSFSRKEQGIIHMVAGLGFEWALHPILDAG : 627
NtabCAMTA1 : LQQWLSSKLQVQNNQMGYSFSRKEQGIIHMVAVLGFEWALQPILDAG : 647
NtabCAMTA1 : FHEWLLERVI--GGCKTSEWDEQGQGVIHLCAILGYTWAVYPFSWSG : 694
NtabCAMTA1 : FHEWLLERVI--GGCKTSEWDEQGQGVIHLCAILGYTWAVYPFSWSG : 563
NtabCAMTA1 : FHEWLLERVI--GGCKTSEWDEQGQGVIHLCAILGYTWAVYPFSWSG : 642
NtomCAMTA1 : FYAWLVRQVT-DDGRGRTAIDDEGQGVLHLAAALGYDWALKPILASG : 706
NtomCAMTA2 : LRVWLLQKVA-EGGKGPNVLDEGGQGVLHFAAALGYDWAIPPTIAAG : 758
NtomCAMTA3 : FQQWLSYKLQRKDNQMGCSLSKKEQGIIHMVSGLGFEWALHPILNAA : 618
NtomCAMTA4 : LKQWLSSKLQVKNNQMGYSFSRKEQGIIHMVAGLGFEWALHPILDAG : 625
NtomCAMTA5 : FHEWLLERVI--GGCKTSEWDEQGQGVIHLCAILGYTWAVYPFSWSG : 647
NbenCAMTA1 : FYAWLVRQVT-DDGRGRTAIDDEGQGVLHLAAALGYDWALKSILASG : 758
NbenCAMTA2 : LRVWLLQKVA-EGGKGPNVLDEGGQGVLHFAAALGYDWAIPPTIATG : 802
NbenCAMTA3 : FQQLLSYKLQRKDNQMGCSLSKKEQGIIHMVSGLGFEWALHPILNAG : 618
NbenCAMTA4 : LQQWLSSKLHVKNNQMGYSFSRKEQGIIHMVAVLGFEWALQPILDAG : 585
NbenCAMTA5 : FHEWLLERVI--GGCKASEWDEQGQGVIHLCAILGYTWAVYPFSWSG : 439
                 wL                      QG   H  a LG  Wa   p    g

                 1040         *       1060          *       1080
NsylCAMTA1 : VSVDFRDMNGWTALHWAAFYGREKTVVGL-VSLGASPGALTDPSAEF : 727
NsylCAMTA2 : VSVNFRDVNGWTALHWAASYGRERTVGFLIISLGAAPGALTDPTPKH : 797
NsylCAMTA3 : INPNLRDARGRTALHWAAHYGREDMVIAL-VKLGVAVCAVDDPTAAF : 692
NsylCAMTA4 : VSVDFRDINGWTALHWAARFGREQMVASL-IASGASAGAVTDPSPRD : 664
NsylCAMTA5 : VSVNFRDINGWTALHWAARFGREKMVASL-VASGAFAGAVTDPSSQD : 686
NsylCAMTA6 : LSLDYRDKYGWTALHWAAHYGREKMVATL-LSAGAKPNLVTDPTSEN : 688
NtabCAMTA1 : VSVDFRDMNGWTALHWAAFYGREKTVVGL-VSLGASPGALTDPSAEF : 744
NtabCAMTA2 : VSVDFRDMNGWTALHWAAFYGREKTVVGL-VSLGASPGALTDPSAEF : 694
NtabCAMTA3 : VSVNFRDVNGWTALHWAASYGRERTVGFLIISLGAAPGALTDPTPKH : 787
NtabCAMTA4 : VSVNFRDVNGWTALHWAASYGRERTVGFL-ISLGAAPGALTDPTPKH : 791
NtabCAMTA5 : VSVNFRDVNGWTALHWAASYGRERTVGFL-ISLGAAPGALTDPTPKH : 778
NtabCAMTA6 : INPNLRDARGRTALHWAAHYGREDMVIAL-VKLGVAVCAVDDPTAAF : 689
NtabCAMTA7 : VSVDFRDINGWTALHWAARFGREQMVASL-IASGASAGAVTDPSPRD : 662
NtabCAMTA8 : VSVNFRDINGWTALHWAARFGREKMVASL-IASGASAGAVTDPSSRD : 708
NtabCAMTA9 : VGVNFRDINGWTALHWAARFGREKMVASL-VASSAFAGAVTDPSSQD : 673
NtabCAMTA1 : VSVNFRDINGWTALHWAARFGREKMVASL-VASGAFAGAVTDPSSQD : 693
NtabCAMTA1 : LSLDYRDKYGWTALHWAAHYGREKMVATL-LSASAKPNLVTDPTSEN : 740
NtabCAMTA1 : LSLDYRDKYGWTALHWAAHYGREKMVATL-LSAGAKPNLVTDPTSEN : 609
NtabCAMTA1 : LSLDYRDKYGWTALHWAAHYGREKMVATL-LSAGAKPNLVTDPTSEN : 688
NtomCAMTA1 : VSVDFRDMNGWTALHWAAFYGREKTVVGL-VSLGASPGALTDPSAEF : 752
NtomCAMTA2 : VSVNFRDVNGWTALHWAASYGRERTVGFL-ISLGAAPGALTDPTPKH : 804
NtomCAMTA3 : VSVNFRDINGWTALHWAARFGREKMVASL-IASGASAGAVTDPSSRD : 664
NtomCAMTA4 : VGVNFRDINGWTALHWAARFGREKMVASL-VASSAFAGAVTDPSSQD : 671
NtomCAMTA5 : LSLDYRDKYGWTALHWAAHYGREKMVATL-LSAGAKPNLVTDPTSEN : 693
NbenCAMTA1 : VSVDFRDMNGWTALHWAAFYGREKTVVGL-VSLGASPGALTDPSAEF : 804
NbenCAMTA2 : VSVNFRDVNGWTALHWAASYGRERTVGFL-ISLGAAPGALTDPTPKH : 848
NbenCAMTA3 : VSVDFRDINGWTALHWAARFGREQMVASL-IASGASAGAVTDPSPRD : 664
NbenCAMTA4 : VSVNFRDINGWTALHWAARFGREKMVASL-VASGAFAGAVTDPSSQD : 631
NbenCAMTA5 : LSLDYRDKYGWTALHWAAHYGREKMVATL-LSAGAKPNLVTDPTSEN : 485
               s   RD  GwTALHWAA  GRE  V  L    ga   a tDP
```

```
                      *        1100          *        1120
NsylCAMTA1 : PLGRTPADLASANGHKGISGFLAESSLTTHISKLTV-TDAKEELASE :  773
NsylCAMTA2 : PSGRTPADLASSNGHKGIAGYLAESSLSSHISSLEL--KEMKQGETV :  842
NsylCAMTA3 : PGGQTAADLASSGGHKGVAGYLAESELTAHIQSLAINNNALDSICAG :  739
NsylCAMTA4 : PVGKTAASIASSCGHKGLAGYLSEVALTSHISSLTLVESELSKGTAD :  711
NsylCAMTA5 : PFGKTAASIASSCGHKGVAGYLSEVALTSHISSLTLEESELSKGAAD :  733
NsylCAMTA6 : PGGSTAADLASKNGFEGLGAYLAEKALVAHFKDMTL----------- :  724
NtabCAMTA1 : PLGRTPADLASANGHKGISGFLAESSLTTHISKLTV-TDAKEELASE :  790
NtabCAMTA2 : PLGRTPADLASANGHKGISGFLAESSLTTHISKLTV-TDATEELASE :  740
NtabCAMTA3 : PSGRTPADLASSNGHKGIAGYLAESSLSSHISSLEL--KEMKQGETV :  832
NtabCAMTA4 : PSGRTPADLASSNGHKGIAGYLAESSLSFHISSLEL--KEMKQGENV :  836
NtabCAMTA5 : PSGRTPADLASSNGHKGIAGYLAESSLSFHISSLEL--KEMKQGENV :  823
NtabCAMTA6 : PGGQTAADLASSGGHKGVAGYLAESELTAHIQSLAINNNALDSICAG :  736
NtabCAMTA7 : PVGKTAASIASSCGHKGLAGYLSEVALTSHISSLTLVESELSKGTAD :  709
NtabCAMTA8 : PVGKTAASIASSCGHKGLAGYLSEVALTSHISSLTLEESELSKGTAD :  755
NtabCAMTA9 : PFGRTAASIASSCGHKGVAGYLSEVALTSHISSLTLEENELSKGTAD :  720
NtabCAMTA1 : PFGKTAASIASSCGHKGVAGYLSEVALTSHISSLTLEESELSKGAAD :  740
NtabCAMTA1 : PGGSTAADLASKNGFEGLGAYLAEKALVAHFKDMTL----------- :  776
NtabCAMTA1 : PGGSTAADLASKNGFEGLGAYLAEKALVAHFKDMTL----------- :  645
NtabCAMTA1 : PGGSTAADLASKNGFEGLGAYLAEKALVAHFKDMTL----------- :  724
NtomCAMTA1 : PLGRTPADLASANGHKGISGFLAESSLTTHISKLTV-TDATEELASE :  798
NtomCAMTA2 : PSGRTPADLASSNGHKGIAGYLAESSLSFHISSLEL--KEMKQGENV :  849
NtomCAMTA3 : PVGKTAASIASSCGHKGLAGYLSEVALTSHISSLTLEESELSKGTAD :  711
NtomCAMTA4 : PFGRTAASIASSCGHKGVAGYLSEVALTSHISSLTLEENELSKGTAD :  718
NtomCAMTA5 : PGGSTAADLASKNGFEGLGAYLAEKALVAHFKDMTL----------- :  729
NbenCAMTA1 : PLGRTPADLASANGHKGISGFLAESSLTTHISKLTV-TDAKEELASE :  850
NbenCAMTA2 : PSGRTPADLASSNGHKGIAGYLAESSLSSHISSLEL--KEMKQGENV :  893
NbenCAMTA3 : PVGKTAASIASSCGHKGLAGYLSEVALTSHISSLTLVESELSKGTAD :  711
NbenCAMTA4 : PFGKTAASIASSCGHKGVAGFLSEVALTSHISSLTLEESELSKGTAD :  678
NbenCAMTA5 : PGGSTVADLASKNGFEGLGAYLAEKALVAHFKDMSL----------- :  521
             P G T A  AS  GhkG  gyL E  L  Hl  l

             *        1140         *        1160          *
NsylCAMTA1 : VSGAKVGETVTERVAVTTTGDDMPDVLSLKDSLAAIRNATQAAARIH :  820
NsylCAMTA2 : QPFGEAVQTVSERSATPAWDGDWPHGVSLKDSLAAVRNATQAAARIH :  889
NsylCAMTA3 : LEAEKAFESAAQEVVPLN--GTIHDDISLKGSLASVRKSAHAAALIQ :  784
NsylCAMTA4 : VEAEKTISSISNTSATTN-----EDQRSLKDSLAAVRNAAQAAARIQ :  753
NsylCAMTA5 : VEAERTISSISTTNAATH-----EDQLSLKDTLAAVRNAAQAAARIQ :  775
NsylCAMTA6 : --AGNVSGSLQTTTEHINPGNFTEEELYLKDTLAAYRTAADAAARIQ :  769
NtabCAMTA1 : VSGAKVGETVTERVAVTTTGDDMPDVLSLKDSLAAIRNATQAAARIH :  837
NtabCAMTA2 : VSGAKVGETVTERVAVTTTGDDVPDVLSLKDSLAAIRNATQAAARIH :  787
NtabCAMTA3 : QPFGEAVQTVSERSATPAWDGDWPHGVSLKDSLAAVRNATQAAARIH :  879
NtabCAMTA4 : QPFGEAVQTVSERSATPAWDGDWPHGVSLKDSLAAVRNATQAAARIH :  883
NtabCAMTA5 : QPFGEAVQTVSERSATPAWDGDWPHGVSLKDSLAAVRNATQAAARIH :  870
NtabCAMTA6 : LEAEKAFESAAQEVVPLN--GTIHDDISLKGSLASVRKSAHAAALIQ :  781
NtabCAMTA7 : VEAEKTISSISNTSATTN-----EDQRSLKDSLAAVRNAAQAAARIQ :  751
NtabCAMTA8 : VEAEKTISSISNTSATTN-----EDQRSLKDSLAAVRNAAQAAARIQ :  797
NtabCAMTA9 : VEAERTISSISTTSAATH-----EDQLSLKDTLAAVRNAAQAAARIQ :  762
NtabCAMTA1 : VEAERTISSISTTNAATH-----EDQLSLKDTLAAVRNAAQAAARIQ :  782
NtabCAMTA1 : --AGNVSGSLQTTTEHINPGNFTEEELYLKDTLAAYRTAADAAARIQ :  821
NtabCAMTA1 : --AGNVSGSLQTTTEHINPGNFTEEELYLKDTLAAYRTAADAAARIQ :  690
NtabCAMTA1 : --AGNVSGSLQTTTEHINPGNFTEEELYLKDTLAAYRTAADAAARIQ :  769
NtomCAMTA1 : VSGAKVGETVTERVAVTTTGDDVPDVLSLKDSLAAIRNATQAAARIH :  845
NtomCAMTA2 : QPFGEAVQTVSERSATPAWDGDWPHGVSLKDSLAAVRNATQAAARIH :  896
NtomCAMTA3 : VEAEKTISSISNTSATTN-----EDQRSLKDSLAAVRNAAQAAARIQ :  753
NtomCAMTA4 : VEAERTISSISTTSAATH-----EDQLSLKDTLAAVRNAAQAAARIQ :  760
NtomCAMTA5 : --AGNVSGSLQTTTEHINSGNFTEEELYLKDTLAAYRTAADAAARIQ :  774
NbenCAMTA1 : VSGAKVGETITERVAVTTTGDEMPDVLSLKDSLAAIRNATQAAARIH :  897
NbenCAMTA2 : QPFGEAVQTVSERSATPAWDGDWPHGVSLKDSLAAVRNATQAAARIH :  940
NbenCAMTA3 : VEAEKTISSISNTSSTTN-----EDQRSLKDSLAAVRNAAQAAARIQ :  753
NbenCAMTA4 : VEAERTISSISTKNVATH-----EDQLSLKDTLAAVRNAAQAAARIQ :  720
NbenCAMTA5 : --AGNVSGSLQTTTEHINPGNFTEEELYLRDTLAAYRTAADAAARIQ :  566
                                        sLkd LAa R a  AAArI
```

```
                   1180          *        1200           *        1220
NsylCAMTA1 : QIFRVQSFQ-RKQIIEC------SDNELS-SDENALSIVASRA-CKL :  858
NsylCAMTA2 : QVFRVQSFQ-RKQLKEH------GGSEFGLSDEHALSLLALKT-NKA :  928
NsylCAMTA3 : AAFRARSFH-QRQLRE-------SRNDVSEASVDLVALGSLNKVQKV :  823
NsylCAMTA4 : SAFRAHSFR-KRQQRESAIATTASGDEYGILSNDILGLSAASK-WAF :  798
NsylCAMTA5 : SAFRAHSFR-KRRQREAARAATTSGDEYCVLSNDVLGLSAASK-LAF :  820
NsylCAMTA6 : AAFREHSFKVQTKAVES------SNPEM--EARNIVAAMKI-Q-HAF :  806
NtabCAMTA1 : QIFRVQSFQ-RKQIIEC------SDNELS-SDENALSIVASRA-CKL :  875
NtabCAMTA2 : QIFRVQSFQ-RKQIIER------SDNELS-SDENALSIVASRA-CKL :  825
NtabCAMTA3 : QVFRVQSFQ-RKQLKEH------GGSEFGLSDEHALSLLALKT-NKA :  918
NtabCAMTA4 : QVFRVQSFQ-RKQLKEH------GGSEFGLSDEHALSLLALKT-NKA :  922
NtabCAMTA5 : QVFRVQSFQ-RKQLKEH------GGSEFGLSDEHALSLLALKT-NKA :  909
NtabCAMTA6 : AAFRARSFH-QRQLRE-------SRNDVSEASVDLVALGSLNKVQKV :  820
NtabCAMTA7 : SAFRAHSFR-KRQQRESAIATTASGDEYGILSNDILGLSAASK-WAF :  796
NtabCAMTA8 : SAFRAHSFR-KRQQRESAVTATASGDEYGILSNNIHGLSAASK-WAF :  842
NtabCAMTA9 : SAFRAHSFR-KRRQREAARAATTSGDEYCVLSNDVLGLSAASK-LAF :  807
NtabCAMTA1 : SAFRAHSFR-KRRQREAARAATTSGDEYCVLSNDVLGLSAASK-LAF :  827
NtabCAMTA1 : AAFREHSFKVQTKAVES------SNPEI--EARNIVAAMKI-Q-HAF :  858
NtabCAMTA1 : AAFREHSFKVQTKAVES------SNPEM--EARNIVAAMKI-Q-HAF :  727
NtabCAMTA1 : AAFREHSFKVQTKAVES------SNPEM--EARNIVAAMKI-Q-HAF :  806
NtomCAMTA1 : QIFRVQSFQ-RKQIIER------SDNELS-SDENALSIVASRA-CKL :  883
NtomCAMTA2 : QVFRVQSFQ-RKQLKEH------GGSEFGLSDEHALSLLALKT-NKA :  935
NtomCAMTA3 : SAFRAHSFR-KRQQRESAVTATASGDEYGILSNDIHGLSAASK-WAF :  798
NtomCAMTA4 : SAFRAHSFR-KRRQREAARAATTSGDEYCVLSNDVLGLSAASK-LAF :  805
NtomCAMTA5 : AAFREHSFKVQTKAVES------SNPEI--EARNIVAAMKI-Q-HAF :  811
NbenCAMTA1 : QIFRVQSFQ-RKQIIEC------SDNELS-FDENALSIVASRA-CKL :  935
NbenCAMTA2 : QVFRVQSFQ-RKQLKEH------GGSEFGLSDEHALSLLALKT-NKA :  979
NbenCAMTA3 : SAFRAHSFR-KRQQRESAVATTACGDEYGFLSNDIHGLSAASK-WAF :  798
NbenCAMTA4 : SAFRAHSFR-KRRQREAARAATTSGDEYCVLSNDVLGLSAASK-LAF :  765
NbenCAMTA5 : AAFREHSFKVQTKAVES------SNPEM--EARNIVAAMKI-Q-HAF :  603
               FR  SF       E          e

                     *        1240          *         1260
NsylCAMTA1 : GQNNGIAH-AAATQIQKKFRGWNKRKEFLLIRQKIVKIQAHVRGHQV :  904
NsylCAMTA2 : GQHDEPVH-TAAVRIQNKFRSWKGRRDYLLIRQRIIKIQAHVRGHQV :  974
NsylCAMTA3 : NHFEDYLH-PAAIKIQQKYRGWKGRREFLKIRNRIVKIQAHVRGHQV :  869
NsylCAMTA4 : RNTRDY-N-SAALAIQKKYRGWKGRKDFLAFRQKVVKIQAHVRGYQV :  843
NsylCAMTA5 : RNMRDY-N-SAALAIQKKYRGWKCRKGFLAFRQKVVKIQAHVRGYQV :  865
NsylCAMTA6 : RNYESRKKLAAAARIQYRFRSWKMRKDFLNMRRHAIKIQAVFRGFQV :  853
NtabCAMTA1 : GQNNGIAH-AAATQIQKKFRGWNKRKEFLLIRQKIVKIQAHVRGHQV :  921
NtabCAMTA2 : GQNNGIAH-AAATQIQKKFRGWNKRKEFLLIRQKIVKIQAHVRGHQV :  871
NtabCAMTA3 : GQHDEPVH-TAAVRIQNKFRSWKGRRDYLLIRQRIIKIQAHVRGHQV :  964
NtabCAMTA4 : GQHDEPVH-TAAVRIQNKFRSWKGRRDYLLIRQRIIKIQAHVRGHQV :  968
NtabCAMTA5 : GQHDEPVH-TAAVRIQNKFRSWKGRRDYLLIRQRIIKIQAHVRGHQV :  955
NtabCAMTA6 : NHFEDYLH-PAAIKIQQKYRGWKGRREFLKIRNRIVKIQAHVRGHQV :  866
NtabCAMTA7 : RNTRDY-N-SAALAIQKKYRGWKGRKDFLAFRQKVVKIQAHVRGYQV :  841
NtabCAMTA8 : RNTRDY-N-SAALAIQKKYRGWKGRKDFLAFRQKVVKIQAHVRGYQV :  887
NtabCAMTA9 : RNMRDY-N-SAALAIQKKYRGWKCRKDFLAFRQKVVKIQAHVRGYQV :  852
NtabCAMTA1 : RNMRDY-N-SAALAIQKKYRGWKCRKDFLAFRQKVVKIQAHVRGYQV :  872
NtabCAMTA1 : RNYESRKKLAAAARIQYRFRSWKMRKDFLNMRRHAIKIQAVFRGFQV :  905
NtabCAMTA1 : RNYESRKKLAAAARIQYRFRSWKMRKDFLNMRRHAIKIQAVFRGFQV :  774
NtabCAMTA1 : RNYESRKKLAAAARIQYRFRSWKMRKDFLNMRRHAIKIQAVFRGFQV :  853
NtomCAMTA1 : GQNNGIAH-AAATQIQKKFRGWNKRKEFLLIRQKIVKIQAHVRGHQV :  929
NtomCAMTA2 : GQHDEPVH-TAAVRIQNKFRSWKGRRDYLLIRQRIIKIQAHVRGHQV :  981
NtomCAMTA3 : RNTRDY-N-SAALAIQKKYRGWKGRKDFLAFRQKVVKIQAHVRGYQV :  843
NtomCAMTA4 : RNMRDY-N-SAALAIQKKYRGWKCRKDFLAFRQKVVKIQAHVRGYQV :  850
NtomCAMTA5 : RNYESRKKLAAAARIQYRFRSWKMRKDFLNMRRHAIKIQAVFRGFQV :  858
NbenCAMTA1 : GQNNGLAH-AAATQIQKKFRGWNKRKEFLLIRQKIVKIQAHVRGHQV :  981
NbenCAMTA2 : GQHDEPVH-TAAVRIQNKFRSWKGRRDYLLIRQRIIKIQAHVRGHQV : 1025
NbenCAMTA3 : RNTRDY-N-TAALAIQKKYRGWKGRKDFLAFRQKVVKIQAHVRGYQV :  843
NbenCAMTA4 : RNMRDY-N-SAALAIQKKYRGWKCRKDFLAFRHKVVKIQAHVRGYQV :  810
NbenCAMTA5 : RNYESRKKLAAAARIQYRFRSWKMRKDFLNMRRHAITIQAVFRGFQV :  650
                       AA  IQ k R Wk R  fL  R    kIQAhvRG QV
```

```
                  *         1280          *         1300          *
NsylCAMTA1  : RKKYKPIIWSVGILEKVILRWRRKRSGLRGFRSEVVMNK-PIIQDDS :  950
NsylCAMTA2  : RNKYKNIIWSVGILEKVILRWRRKGSGLRGFKPEATLTEGSNMQDRP : 1021
NsylCAMTA3  : RKQYKKFVWSVSIVEKAILRWRRKKPGLRGFQPEKT----SQKELPE :  912
NsylCAMTA4  : RKQYK-VCWAVGILEKVVLRWRRRGVGLRGFRHD----------AES :  879
NsylCAMTA5  : RKEYK-VCWAVGILEKVVLRWRRRGVGLRGFRLE----------EEP :  901
NsylCAMTA6  : RKQYRKIVWSVGVLEKAVLRWRLKRKGFRGLQVQSSQAV-DIKPDGD :  899
NtabCAMTA1  : RKKYKPIIWSVGILEKVILRWRRKRSGLRGFRSEVVMNK-PIIQDDS :  967
NtabCAMTA2  : RKKYKPIIWSVGILEKVILRWRRKRSGLRGFRSEVVINK-PSIQDDS :  917
NtabCAMTA3  : RNKYKNIIWSVGILEKVILRWRRKGSGLRGFKPEATLTEGSNMQDRP : 1011
NtabCAMTA4  : RNKYKNIIWSVGILEKVILRWRRKGSGLRGFKPEATLTEGSDTQDRP : 1015
NtabCAMTA5  : RNKYKNIIWSVGILEKVILRWRRKGSGLRGFKPEATLTEGSNMQDRP : 1002
NtabCAMTA6  : RKQYKKFVWSVSIVEKAILRWRRKKPGLRGFQPEKT----SQKELPE :  909
NtabCAMTA7  : RKQYK-VCWAVGILEKVVLRWRRRGVGLRGFRHD----------AES :  877
NtabCAMTA8  : RKQYK-VCWAVGILEKVVLRWRRRGVGLRGFRHD----------TES :  923
NtabCAMTA9  : RKEYK-VCWAVGILEKVVLRWRRRGVGLRGFRLE----------EEP :  888
NtabCAMTA1  : RKEYK-VCWAVGILEKVVLRWRRRGVGLRGFRLE----------EEP :  908
NtabCAMTA1  : RKQYRKIVWSVGVLEKAVLRWRLKRKGFRGLQVQSSQAV-DIKPDGD :  951
NtabCAMTA1  : RKQYRKIVWSVGVLEKAVLRWRLKRKGFRGLQVQSSQAV-DIKPDGD :  820
NtabCAMTA1  : RKQYRKIVWSVGVLEKAVLRWRLKRKGFRGLQVQSSQAV-DIKPDGD :  899
NtomCAMTA1  : RKKYKPIIWSVGILEKVILRWRRKRSGLRGFRSEVVINK-PSIQDDS :  975
NtomCAMTA2  : RNKYKNIIWSVGILEKVILRWRRKGSGLRGFKPEATLTEGSNTQDRP : 1028
NtomCAMTA3  : RKQYK-VCWAVGILEKVVLRWRRRGVGLRGFRHD----------TES :  879
NtomCAMTA4  : RKEYK-VCWAVGILEKVVLRWRRRGVGLRGFRLE----------EEP :  886
NtomCAMTA5  : RKQYRKIVWSVGVLEKAVLRWRLKRKGFRGLQVQSSQAV-DIKPDGD :  904
NbenCAMTA1  : RKKYKPIIWSVGILEKVILRWRRKRSGLRGFRSEVVMNK-PSIQDDS : 1027
NbenCAMTA2  : RNKYKNIIWSVGILEKVILRWRRKGSGLRGFKPEATLTEGSNTQDRP : 1072
NbenCAMTA3  : RKQYK-VCWAVGILEKVVLRWRRRGVGLRGFRHD----------AES :  879
NbenCAMTA4  : RKEYK-VCWAVGILEKVVLRWRRRGVGLRGFRLE----------EES :  846
NbenCAMTA5  : RKQYRKIVWSVGVLEKAVLRWRLKRKGFRGLQVQSSQAV-DIKPDGD :  696
              Rk Yk    W VgilEK  LRWRr   GlRGf

               1320          *        1340          *        1360
NsylCAMTA1  : LPE-DDYDFLKEGRKQT-EVRMQKALARVKSMTQYPEGRAQYRRLLT :  995
NsylCAMTA2  : VQE-DDYDFLKEGRKQT-EQRLQKALARVKSMVQYPEARDQYRRLLN : 1066
NsylCAMTA3  : FEKNDEYEYLSIGRKQK-FAGVQKALARVQSMVRHPEARDQYMRLVA :  958
NsylCAMTA4  : IDESEDEDILKVFRKQKVDAALDEAVSRVLSMVESPGARQQYHRILE :  926
NsylCAMTA5  : IEESEDEDILKLFRKQKVDAAINEAVSRVLSMVDSPEARQQYHRILE :  948
NsylCAMTA6  : VEE----DFFRASRKQA-EERVERSVVRVQAMFRSKRAQEEYRRMKL :  941
NtabCAMTA1  : LPE-DDYDFLKEGRKHT-EVRMQKALARVKSMTQYPEGRAQYRRLLT : 1012
NtabCAMTA2  : LPE-DDYDFLKEGRKQT-EVRMQKALARVKSMTQYPEGRAQYRRLLT :  962
NtabCAMTA3  : VQE-DDYDFLKEGRKQT-EQRLQKALARVKSMVQYPEARDQYRRLLN : 1056
NtabCAMTA4  : VQE-DDYDFLKEGRKQT-EQRLQKALARVKSMVQYPEARDQYRRLLN : 1060
NtabCAMTA5  : VQE-DDYDFLKEGRKQT-EQRLQKALARVKSMVQYPEARDQYRRLLN : 1047
NtabCAMTA6  : FEKNDEYEYLSIGRKQK-FAGVQKALARVQSMVRHPEARDQYMRLVA :  955
NtabCAMTA7  : IDESEDEDILKVFRKQKVDAALDEAVSRVLSMVESPGARQQYHRILE :  924
NtabCAMTA8  : IDESEDEDILKVFRKQKVDAALDEAVSRVLSMVESPGARQQYHRILE :  970
NtabCAMTA9  : IEESEDEDILKLFRKQKVDAAINEAVSRVLSMVDSPEARQQYHRILE :  935
NtabCAMTA1  : IEESEDEDILKLFRKQKVDAAINEAVSRVLSMVDSPEARQQYHRILE :  955
NtabCAMTA1  : VEE----DFFRASRKQA-EERVERSVVRVQAMFRSKRAQEEYRRMKL :  993
NtabCAMTA1  : VEE----DFFRASRKQA-EERVERSVVRVQAMFRSKRAQEEYRRMKL :  862
NtabCAMTA1  : VEE----DFFRASRKQA-EERVERSVVRVQAMFRSKRAQEEYRRMKL :  941
NtomCAMTA1  : LPE-DDYDFLKEGRKQT-EVRMQKALARVKSMTQYPEGRAQYRRLLT : 1020
NtomCAMTA2  : VQE-DDYDFLKEGRKQT-EQRLQKALARVKSMVQYPEARDQYRRLLN : 1073
NtomCAMTA3  : IDESEDEDILKVFRKQKVDAALDEAVSRVLSMVESPGARQQYHRILE :  926
NtomCAMTA4  : IEESEDEDILKLFRKQKVDAAINEAVSRVLSMVDSPEARQQYHRILE :  933
NtomCAMTA5  : VEE----DFFRASRKQA-EERVERSVVRVQAMFRSKRAQEEYRRMKL :  946
NbenCAMTA1  : LPE-DDYDFLKEGRKQT-EVRMQKALSRVKSMTQYPEGRAQYRRLLT : 1072
NbenCAMTA2  : VQE-DDYDFLKEGRKQT-EQRLQKALARVKSMVQYPEARDQYRRLLN : 1117
NbenCAMTA3  : VDESEDEDILKVFRKQKVDAALDEAVSRVLSMVESPGARRQYHRILE :  926
NbenCAMTA4  : IEESEDEDILKLFRKQKVDAAINEAVSRVLSMVDSPEARQQYHRILE :  893
NbenCAMTA5  : VEE----DFFRASRKQA-EERVERSVVRVQAMFRSKRAQEEYRRMKL :  738
                e     d l   RKq         a   RV sM    p ar qY R
```

```
                           *       1380           *       1400          *
NsylCAMTA1 : AAEGLR----------------------------------------- : 1001
NsylCAMTA2 : VVSDMK---------------DTT----------------------- : 1075
NsylCAMTA3 : KFDSFK----------------------------------------- :  964
NsylCAMTA4 : KYRQAK----------------------------------------- :  932
NsylCAMTA5 : KYRQAK---------------------------------AKSRRMVD :  962
NsylCAMTA6 : EHDNAT----------------------------------------- :  947
NtabCAMTA1 : AAEGLR----------------------------------------- : 1018
NtabCAMTA2 : AAEGLR----------------------------------------- :  968
NtabCAMTA3 : VVSDMK---------------DTT----------------------- : 1065
NtabCAMTA4 : VVSDMK---------------DTT----------------------- : 1069
NtabCAMTA5 : VVSDMK---------------DTT----------------------- : 1056
NtabCAMTA6 : KFDSFK---------------ELM----------------------- :  964
NtabCAMTA7 : KYRQAK----------------------------------------- :  930
NtabCAMTA8 : KYRQAK----------------------------------------- :  976
NtabCAMTA9 : KYRQAKGKPTDGTTGRSNGRSSSTTKHAKPGRNARGRGGGGHHAIVG :  982
NtabCAMTA1 : KYRQAK----------------------------------------- :  961
NtabCAMTA1 : EHDNAT----------------------------------------- :  999
NtabCAMTA1 : EHDNAT----------------------------------------- :  868
NtabCAMTA1 : EHDNAT----------------------------------------- :  947
NtomCAMTA1 : AAEGLR----------------------------------------- : 1026
NtomCAMTA2 : VVSDMK---------------DTTLDFRLAFFNVNGVFMESKTQNLT : 1105
NtomCAMTA3 : KYRQAK----------------------------------------- :  932
NtomCAMTA4 : KYRQAKGKPTDGTTGRPNGRSSSTTKHAKPGRNARGRGGGGHHAIVG :  980
NtomCAMTA5 : EHDNAT----------------------------------------- :  952
NbenCAMTA1 : AAEGLR----------------------------------------- : 1078
NbenCAMTA2 : VVSDMK---------------DTT----------------------- : 1126
NbenCAMTA3 : KYRQAK----------------------------------------- :  932
NbenCAMTA4 : KYRQAK----------------------------------------- :  899
NbenCAMTA5 : EHDSAT----------------------------------------- :  744


                     1420         *       1440          *       1
NsylCAMTA1 : --------EVKPDGPTCILESPED-------------TSYPEEELFD : 1027
NsylCAMTA2 : --------TTSDGAPSNSGEAADF-----------------GDDLID : 1097
NsylCAMTA3 : ----ELMAKAKCGKLYSYCAGSHM-----------------EGIEII :  990
NsylCAMTA4 : --------AELEGGESETASTAHG----------------------D :  949
NsylCAMTA5 : HAKWQARRKALEEVPAQGFDVVAELEIDKSEETKTWILAYPEKDSKS : 1009
NsylCAMTA6 : -------------------------------------------LEYE :  951
NtabCAMTA1 : --------EVKPDGPTCILESPED-------------TSYPEEELFD : 1044
NtabCAMTA2 : ---------EVKDGSTCIQESSED-------------TSYPEEELFD :  993
NtabCAMTA3 : --------TTSDGAPSNSGEAADF-----------------GDDLID : 1087
NtabCAMTA4 : --------TTSDGAPSNSVEAADF-----------------GDDLID : 1091
NtabCAMTA5 : --------TTSDGAPSNSVEAADF-----------------GDDLID : 1078
NtabCAMTA6 : -------AKAKCGKLYSYCAGSHM----------------EGCELLH :  988
NtabCAMTA7 : --------AELEGAESETASTAHG----------------------D :  947
NtabCAMTA8 : --------AELEGAESESASTAHG----------------------D :  993
NtabCAMTA9 : GTISGIF-KPKRGKNTNIQGEARQKIYLQLSSDTLEVLTGHKTSTCP : 1028
NtabCAMTA1 : ---------AELGVNSDTVSTAHG----------------------- :  976
NtabCAMTA1 : -------------------------------------------LEYE : 1003
NtabCAMTA1 : -------------------------------------------LEYE :  872
NtabCAMTA1 : -------------------------------------------LEYE :  951
NtomCAMTA1 : ----------------------------------------------- :    -
NtomCAMTA2 : WSSLSVMQTTSDGAPSNSVEAADF-----------------GDDLID : 1135
NtomCAMTA3 : --------AELEGAESESASTAHG----------------------D :  949
NtomCAMTA4 : GTISGIF-KPKRGKNTNIQGEARQKIYLQLSSDTLEVLTGHKTSTCP : 1026
NtomCAMTA5 : -------------------------------------------LEYE :  956
NbenCAMTA1 : ---------EVKDVPTCILESPED-------------TSYPEEELFD : 1103
NbenCAMTA2 : --------TSSDGAPSNSGEAADF-----------------GDDLID : 1148
NbenCAMTA3 : --------AELEGAESETASTAHG----------------------D :  949
NbenCAMTA4 : ---------AELGVNSDTVSTAHG----------------------- :  914
NbenCAMTA5 : -------------------------------------------LEYE :  748
```

```
               460         *      1480
NsylCAMTA1 : VENLL------DDDTFMSIAFE------- : 1043
NsylCAMTA2 : LDDLL------DDDTFMSTAP-------- : 1112
NsylCAMTA3 : LQAAV------------------------ :  995
NsylCAMTA4 : MSNME------NDDIYQFPSY-------- :  964
NsylCAMTA5 : LSGFEDWEDLENGDVSSDEDQAT------ : 1032
NsylCAMTA6 : RASLL------NPDIQIG----------- :  963
NtabCAMTA1 : VENLL------DDDTFMSIAFE------- : 1060
NtabCAMTA2 : VENLL------DDDTFMSIAFE------- : 1009
NtabCAMTA3 : LDDLL------DDDTFMSTAP-------- : 1102
NtabCAMTA4 : LDDLL------DDDTFMSTAP-------- : 1106
NtabCAMTA5 : LDDLL------DDDTFMSTAP-------- : 1093
NtabCAMTA6 : LQGRVVGGGISKEHGPISCSKD------- : 1010
NtabCAMTA7 : MSNME------NDDIYQFPSY-------- :  962
NtabCAMTA8 : MSNME------NDDIYQFSSY-------- : 1008
NtabCAMTA9 : YSGEE------CGDIGFIPKEKPQQQPKQ : 1051
NtabCAMTA1 : --DIS------NSDI-------------- :  983
NtabCAMTA1 : RASVL------NPDIQIG----------- : 1015
NtabCAMTA1 : RASLL------NPDIQIG----------- :  884
NtabCAMTA1 : RASLL------NPDIQIG----------- :  963
NtomCAMTA1 : -----------EVKV-------------- : 1030
NtomCAMTA2 : LDDLL------DDDTFMSTAP-------- : 1150
NtomCAMTA3 : MSNME------NDDIYQFPSY-------- :  964
NtomCAMTA4 : YSGEE------CGDIGFIPKEKPQQQPKQ : 1049
NtomCAMTA5 : RASVL------DPDIQIG----------- :  968
NbenCAMTA1 : VENLL------DDDAFMSIAFE------- : 1119
NbenCAMTA2 : LDDLL------DDDTFILSSSLST----- : 1166
NbenCAMTA3 : MSNME------NDDIYQFPSY-------- :  964
NbenCAMTA4 : --DIP------NSDI-------------- :  921
NbenCAMTA5 : RASLL------NPDI-------------- :  757
                          d
```

Supplementary Figure S3. **The maximum-likelihood phylogenetic tree of CAMTA family genes in 60 representative plant species.**

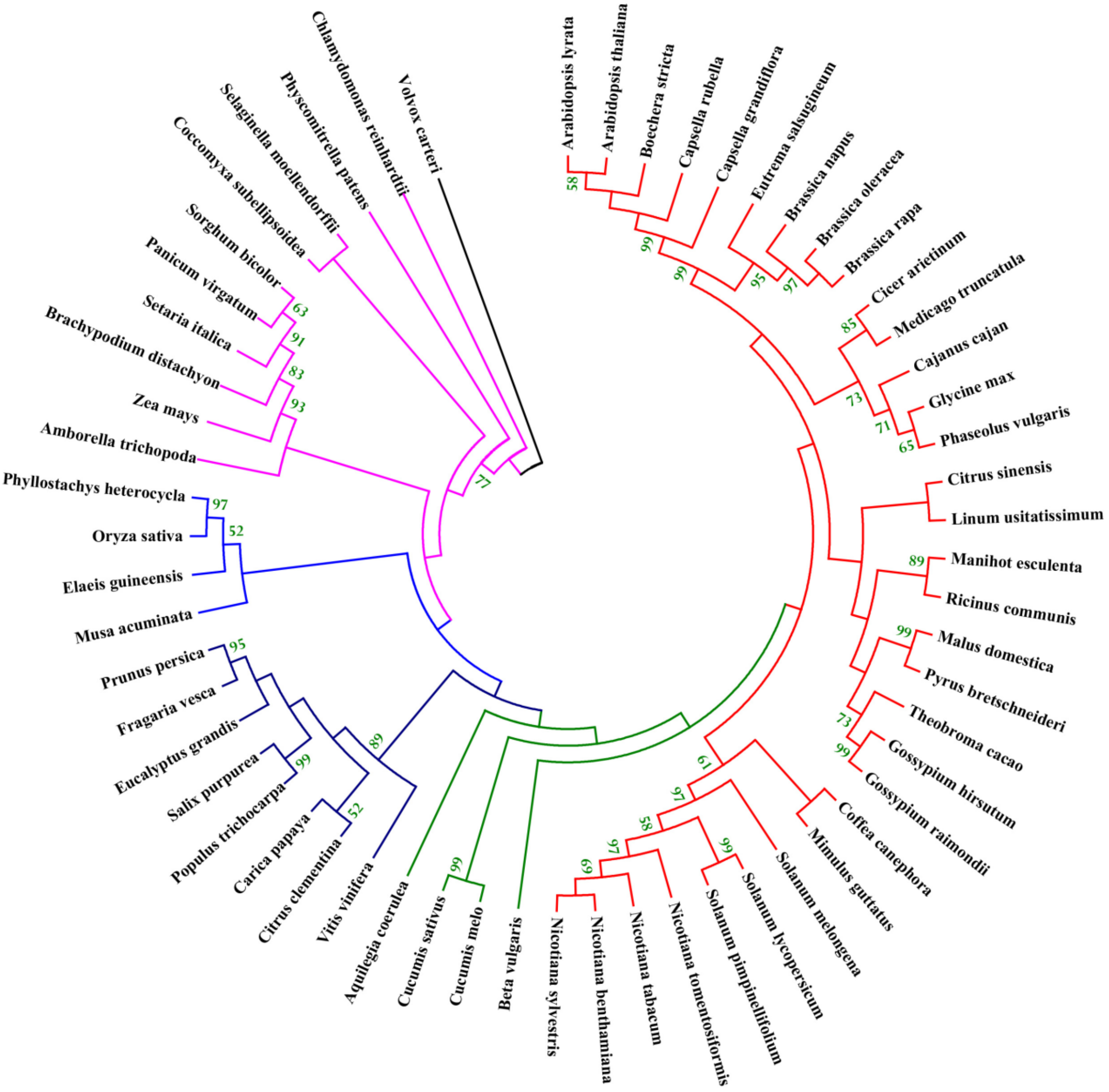

Supplementary Figure S4. Multiple sequence alignment of NtabCAMTA and AtCAMTA proteins. The names of the identified CAMTA genes are indicated to the left of the alignments. The length of the alignment is shown on the top, while the length of each protein is given on the right. Conserved consensus residues are shown in the bottom. Invariable residues conserved at 100% are highlighted in red, >80% are highlighted in blue and residues conserved at >60% are highlighted in green. The sequences were aligned by MEGA6.0 and viewed by GENEDOC program.

```
                      *         20         *         40
NtabCAMTA1 : ------------------------MANGGAC---------------- :  7
NtabCAMTA2 : ----------------------------------------------- :  -
NtabCAMTA3 : ----------------------------------------------- :  -
NtabCAMTA4 : ------------------------MADSRRYGLNA------------ : 11
NtabCAMTA5 : ----------------------------------------------- :  -
NtabCAMTA6 : ----------------------------------------------- :  -
NtabCAMTA7 : ----------------------------------------------- :  -
NtabCAMTA8 : -MQKRASPSIFSPISFLRRARRNSSPSSGNNGSVQGLWQHLPRHPGS : 46
NtabCAMTA9 : ----------------------------------------------- :  -
NtabCAMTA1 : ---------MTELYNKQPTIAPVSSPNTKTVKYSRFQVGSGNRILNE : 38
NtabCAMTA1 : --------------MESSRAGQLAGSDIHGFRTLQGIISVTLILLVY : 33
NtabCAMTA1 : --------------MESSRAGQLAGSDIHGFHTL------------- : 20
NtabCAMTA1 : --------------MESSRAGQLAGSDIHGFHTLQ------------ : 21
AtCAMTA1   : MARKKKSVSFSHLDIARNEGNKIISFSVHAYSDRLGFVDSLFDYESL : 47
AtCAMTA2   : ------------------------MADRGSFGFAP------------ : 11
AtCAMTA3   : ------------------------MAEARRFSPVH------------ : 11
AtCAMTA4   : --------------MSSVAEDNSFTCDIATIFVAICRNPPANPSDSL : 33
AtCAMTA5   : --------------MAGVDSGKLIGSEIHGFHTLQ------------ : 21
AtCAMTA6   : --------------MDGDGLGRLIGSEIHGFHTLQ------------ : 21

               *        60         *        80         *
NtabCAMTA1 : --------------------------ECRLEAVVDGSGSGHFYKRKL : 28
NtabCAMTA2 : -----------------------------------MTVDDW------ :  6
NtabCAMTA3 : -------------------------LNADIDQILLEAQHRWLRPAEI : 22
NtabCAMTA4 : --------------------------QLDIDQILLEAQHRWLRPAEI : 32
NtabCAMTA5 : ----------------------------DIDQILLEAQHRWLRPAEI : 19
NtabCAMTA6 : ----------------------------------MADTRRYL----- :  8
NtabCAMTA7 : ------------------------ISGYDINDLVREAQIRWLKPAEV : 23
NtabCAMTA8 : TP------------------------RYDINDLVREAQIRWLKPAEV : 69
NtabCAMTA9 : ------------------------VSGYNINDLVREGHFRWLRPAEV : 23
NtabCAMTA1 : RGRERAILHHREILLLRI--SMAES-GYNINNLVREGRFRWLRPAEV : 82
NtabCAMTA1 : AEMLGCVLVTKEQFHCNL--GVNSESDLDIPSIMEEAKMRWLRPNEI : 78
NtabCAMTA1 : ----------------------------------------------- :  -
NtabCAMTA1 : --------------------------DLDIPSIMEEAKMRWLRPNEI : 42
AtCAMTA1   : RSLLVDFWVYPSMVDRRSFGSITPPLQLDMEQLLSEAQHRWLRPTEI : 94
AtCAMTA2   : --------------------------RLDIKQLLSEAQHRWLRPAEI : 32
AtCAMTA3   : --------------------------ELDVGQILSEARHRWLRPPEI : 32
AtCAMTA4   : F-------------------------QYEISTLYQEAHSRWLKPPEV : 55
AtCAMTA5   : --------------------------DLDIQTMLDEAYSRWLRPNEI : 42
AtCAMTA6   : --------------------------DLDVQTMLEEAKSRWLRPNEI : 42
                                                e   r l p e
```

```
                   100         *        120         *        140
NtabCAMTA1 : SGSLVLMGILKVF---------------------------------- :  41
NtabCAMTA2 : ----------------------------------------------- :   -
NtabCAMTA3 : CEILKNYQKFRIAPEPPNRP--------------------------- :  42
NtabCAMTA4 : CEILKNYQKFRIAPEPPNRP--------------------------- :  52
NtabCAMTA5 : CEILKNYQKFRIAPEPPNRP--------------------------- :  39
NtabCAMTA6 : ----------------SNQP--------------------------- :  12
NtabCAMTA7 : LFILRNHEYHQLSNEPAQKP--------------------------- :  43
NtabCAMTA8 : LFILRNHENHQLSNEAAQKP--------------------------- :  89
NtabCAMTA9 : VFILQNHEDQQLANQPPQKP--------------------------- :  43
NtabCAMTA1 : LFILQNHEDQQLANQPPQKP--------------------------- : 102
NtabCAMTA1 : HAILCNYKYFNIFVKPVNLP--------------------------- :  98
NtabCAMTA1 : ----------------------------------------------- :   -
NtabCAMTA1 : HAILCNYKYFNIFVKPVNLPMSNTTLSDYLLWSLSCDDFFLVLCSVI :  89
AtCAMTA1   : CEILQNYHKFHIASESPTRP--------------------------- : 114
AtCAMTA2   : CEILRNHQKFHIASEPPNRP--------------------------- :  52
AtCAMTA3   : CEILQNYQRFQISTEPPTTP--------------------------- :  52
AtCAMTA4   : LFILQNHESLTLTNTAPQRP--------------------------- :  75
AtCAMTA5   : HALLCNHKFFTINVKPVNLP--------------------------- :  62
AtCAMTA6   : HAILYNPKYFTINVKPVNLP--------------------------- :  62
                 l n               p

                   *          160        *         180
NtabCAMTA1 : ----------ACGSVFLFDRKVLRYFRKDGHNWRKKKDGKTVKEAHE :  78
NtabCAMTA2 : ----------SSGSVFLFDRKVLRYFRKDGHNWRKKKDGKTVKEAHE :  43
NtabCAMTA3 : ----------PSGSLFLFDRKVLRYFRKDGHSWRKKKDGKTVKEAHE :  79
NtabCAMTA4 : ----------PSGSLFLFDRKVLRYFRKDGHSWRKKKDGKTVKEAHE :  89
NtabCAMTA5 : ----------PSGSLFLFDRKVLRYFRKDGHSWRKKKDGKTVKEAHE :  76
NtabCAMTA6 : ----------LGGSLFLFDRKVLRYFRKDGHQWRKKKDGKTVKEAHE :  49
NtabCAMTA7 : ----------PSGSLFLFNKRVLRFFRKDGHSWRKKKDGRTVGEAHE :  80
NtabCAMTA8 : ----------PSGSLFLFNKRVLRFFRKDGHSWRKKKDGRTVGEAHE : 126
NtabCAMTA9 : ----------ASGSMFLFNKRVLRYFRKDGHSWRKKKDGRTVGEAHE :  80
NtabCAMTA1 : ----------ASGSMFLFNKRVLRYFRKDGHSWRKKKDGRTVGEAHE : 139
NtabCAMTA1 : ----------TSGTIVLFDRKMLRNFRKDGHNWKKKKDGKTVKEAHE : 135
NtabCAMTA1 : ----------QGGTIVLFDRKMLRNFRKDGHNWKKKKDGKTVKEAHE :  57
NtabCAMTA1 : LNEMEHDFSKQSGTIVLFDRKMLRNFRKDGHNWKKKKDGKTVKEAHE : 136
AtCAMTA1   : ----------ASGSLFLFDRKVLRYFRKDGHNWRKKKDGKTIREAHE : 151
AtCAMTA2   : ----------PSGSLFLFDRKVLRYFRKDGHNWRKKKDGKTVKEAHE :  89
AtCAMTA3   : ----------SSGSVFMFDRKVLRYFRKDGHNWRKKKDGKTVKEAHE :  89
AtCAMTA4   : ----------TSGSLLLFNKRVLKFFRKDGHQWRRKRDGRAIAEAHE : 112
AtCAMTA5   : ----------KSGTIVLFDRKMLRNFRKDGHNWKKKKDGKTIKEAHE :  99
AtCAMTA6   : ----------NSGRIILFDRKMLRNFRKDGHNWKKKKDGRTVKEAHE :  99
                       sG 6 6F1446L4 FRKDGH W44K4DG4t6 EAHE

                *       200          *        220         *
NtabCAMTA1 : KLKVSVDSLGISTVGSIDVLHCYYAHGEEDDNFQRRSYWMLEQ---- : 121
NtabCAMTA2 : KLK----------VGSIDVLHCYYAHGEEDDNFQRRSYWMLEQ---- :  76
NtabCAMTA3 : RLKEADQKLMFLQAGSIDVLHCYYAHGEENENFQRRSYWMLEE---- : 122
NtabCAMTA4 : RLK----------AGSIDVLHCYYAHGEENENFQRRSYWMLEE---- : 122
NtabCAMTA5 : RLK----------AGSIDVLHCYYAHGEENENFQRRSYWMLEE---- : 109
NtabCAMTA6 : KLK----------AGSVDVLHCYYAHGENNENFQRRSYWMLEE---- :  82
NtabCAMTA7 : RLK----------VGNAEALNCYYAHGEQNPNFQRRSYWMLDP---- : 113
NtabCAMTA8 : RLK----------VGNAEALNCYYAHGEQNPTFQRRSYWMLDP---- : 159
NtabCAMTA9 : RLK----------VGNAEALNCYYAHGEKNPNFQRRSYWMLDP---- : 113
NtabCAMTA1 : RLK----------VGNAETLNCYYAHGEKNPNFQRRSYWMLDP---- : 172
NtabCAMTA1 : HLK----------VGNEERIHVYYAHGEDHPTFVRRCYWLLDK---- : 168
NtabCAMTA1 : HLK----------VGNEERIHVYYAHGEDHPTFVRRCYWLLDK---- :  90
NtabCAMTA1 : HLK----------VGNEERIHVYYAHGEDHPTFVRRCYWLLDK---- : 169
AtCAMTA1   : KLK----------VGSIDVLHCYYAHGEANENFQRRCYWMLEQYYYR : 188
AtCAMTA2   : KLK----------VGSIDVLHCYYAHGEDNENFQRRCYWMLEQ---- : 122
AtCAMTA3   : RLK----------AGSVDVLHCYYAHGQDNENFQRRSYWLLQE---- : 122
AtCAMTA4   : RLK----------VGNAEALNCYYAHGEQDPTFRRRIYWMLDP---- : 145
AtCAMTA5   : HLK----------VGNEERIHVYYAHGEDTPTFVRRCYWLLDK---- : 132
AtCAMTA6   : HLK----------VGNEERIHVYYAHGEDNTTFVRRCYWLLDK---- : 132
              LK           G    6  YYAHG2    F RR YW6L
```

```
                  240         *        260         *        280
NtabCAMTA1 : --------------------------DLMHIVFVHYLEV-------- :  134
NtabCAMTA2 : --------------------------DLMHIVFVHYLEV-------- :   89
NtabCAMTA3 : --------------------------EMSHIVLVHYREV-------- :  135
NtabCAMTA4 : --------------------------EMSHIVLVHYREV-------- :  135
NtabCAMTA5 : --------------------------EMSHIVLVHYREV-------- :  122
NtabCAMTA6 : --------------------------KLEHIVLVHYREV-------- :   95
NtabCAMTA7 : --------------------------VYEHIVLVHYRDI-------- :  126
NtabCAMTA8 : --------------------------AYEHIVLVHYRDI-------- :  172
NtabCAMTA9 : --------------------------AYEHIVLVHYRDI-------- :  126
NtabCAMTA1 : --------------------------AYEHIVLVHYRDI-------- :  185
NtabCAMTA1 : --------------------------SLEHIVLVHYRETQEVCFLYL :  189
NtabCAMTA1 : --------------------------SLEHIVLVHYRET-------- :  103

                    *        300         *        320
NtabCAMTA1 : -KGNKANVGCVRSIKSAHSNYLNDCSLSD--------------SFPR :  166
NtabCAMTA2 : -KGNKANMGCVRSIKSAHSNYLNDCSLSD--------------SFPR :  121
NtabCAMTA3 : -KGNRTNFSRTREPQEATPRFQET----DEDVHSSEVDSSASTKFYP :  177
NtabCAMTA4 : -KGNRTNFSRTREPQEAAPRFQET----DEDVHSSEVDSSASTKFYP :  177
NtabCAMTA5 : -KGNRTNFSRTREPQEAAPRFQET----DEDVHSSEVDSSASTKFYP :  164
NtabCAMTA6 : ----------IESYRVGASRLQPI----------------------- :  109
NtabCAMTA7 : -TEGRQNPAFMSESSPISSTFSPSPSSYSTQQTGSAVIAGESYEQYQ :  172
NtabCAMTA8 : -TEGRQNPAFMSESSPISSTFSPSPSSYSTQQTGSTLIAGESYEQYQ :  218
NtabCAMTA9 : -TEGMQIAAFMSQSSPISSTFSLSPSLYSTQHPGFTVFGSESYQQYP :  172
NtabCAMTA1 : -TEGMQIAAFMSQSSPISSTFSLSPSLYSTQHPGFTVVGSESYQQYQ :  231
NtabCAMTA1 : ARGEILRAGNLRRKRITYVSCCFMCKCFDEDVDHLLLHFQVVITLLV :  236
NtabCAMTA1 : ---------------------------------------------QE :  105
NtabCAMTA1 : ---------------------------------------------QE :  184
AtCAMTA1   : -KGNRTSIGMKENNS------------------------------NS :  243
AtCAMTA2   : -KGNRMSTSGTKENHSNSLSGTGSVNVDSTATRSSILSPLCEDADSG :  181
AtCAMTA3   : -KGSRVSTSFNRMQRTEDAARSPQ---ETGDALTSEHDGYASCSFNQ :  178
AtCAMTA4   : -------SEREEGQQTGGQVYQFAPILSTQNVS------------YN :  186
AtCAMTA5   : ----------------------------------------------- :    -
AtCAMTA6   : ----------------------------------------------- :    -

              *        340         *        360         *
NtabCAMTA1 : SLKKLASVNADSTSVASTLTS--AHEEAESGNVVCEDSHQACSRFQS :  211
NtabCAMTA2 : GHKKLASANADSTSVASTLTS--AHEEAES-----EDSHQACSRFQS :  161
NtabCAMTA3 : NGYQVNSQVTDATSLSSAQAS--EYEDAES-----AYNQHPTSGFHS :  217
NtabCAMTA4 : NDYQVNSQVTDTTSLSSVQAS--EYEDAES-----AYNQHPTSGFHS :  217
NtabCAMTA5 : NDYQVNSQVTDTTSLSSVQAS--EYEDAES-----AYNQHPTSGFHS :  204
NtabCAMTA6 : ---HPGQLLENPSSSPCFVSGLIVQESHTS---------SPSSVDWK :  144
NtabCAMTA7 : NQFSPGEICSDAV----INNN--RTSDITG-----RTNEVMSSPGLE :  208
NtabCAMTA8 : NQSSPGEICSDAV----INNN--GMSDIIG-----RTKEVMSSPGLE :  254
NtabCAMTA9 : NESSPGSGEVCSDAGINGKGM--NISDITG-----RTEGVSSSPRVE :  212
NtabCAMTA1 : NESSPGSGEICSGAGINSNGM--NISDITG-----RTEGVSSSPQVE :  271
NtabCAMTA1 : TQGSPVTSVAKGSPATPVNSN--SSSDPSD-----PSGWVLSEECNS :  276
NtabCAMTA1 : AQGSPATSVAKGSPATPVNSN--SSSDPSD-----PSGWVLSEKCNS :  145
NtabCAMTA1 : AQGSPATSVAKGSPATPVNSN--SSSDPSD-----PSGWVLSEKCNS :  224
AtCAMTA1   : VNGTASVNIDSTASPTSTLSS--LCEDADT----VLVQGIVNKQVPS :  284
AtCAMTA2   : DSRQASSSLQQNPEPQTVVPQIMHHQNASTINSYNTTSVLGNRDGWT :  228
AtCAMTA3   : NDHSNHSQTTDSASVNGFHSP--ELEDAES-----AYNQHGSSTAYS :  218
AtCAMTA4   : QYIGDSSDIYQQSSTSPGVAE--VNSNLEG-----------SASSSE :  220
AtCAMTA5   : -------HEVHAAPATPGNSY--SSSITDH-----------LSPKIV :  172
AtCAMTA6   : -------------------------QEAAT-----TSGDSISSPISV :  162
```

```
                    380         *        400         *        420
NtabCAMTA1 : YPERASGMDRHLVENRDAIY---SSYGS------------------- :  236
NtabCAMTA2 : YPERASGMDRNLVENRDTIY---SSYGS------------------- :  186
NtabCAMTA3 : FLDAQPSMMQKAGESLPVPYHPIPFSNDHQVQFAGSSDMDFFSSAPG :  264
NtabCAMTA4 : FLDAQPSMTQKAGEGLAVPYHPIPFSNDHQVQFAGSSDMDFFSIAPG :  264
NtabCAMTA5 : FLDAQPSMTQKAGEGLAVPYHPIPFSNDHQVQFAGSSDMDFFSIAPG :  251
NtabCAMTA6 : EQALSSELYTGDSKGNEVNPLLVPASGH------------------- :  172
NtabCAMTA7 : MSQALRRLEEQLSLNDDSFKEIDPLY--------------------- :  234
NtabCAMTA8 : MSQALRRLEEQLSLNDDSFKEIDPLY--------------------- :  280
NtabCAMTA9 : ISQALRKLEEQLSLNDDSLEQIDPLY--------------------- :  238
NtabCAMTA1 : ISQALRKLEEQLSLNETDPLY-------------------------- :  292
NtabCAMTA1 : VDERAYGSSQHAHLE---PNRDMTAKNH------------------- :  301
NtabCAMTA1 : VDERTYGSSQHAHLE---PNRDVTAKNH------------------- :  170
NtabCAMTA1 : VDERTYGSSQHAHLE---PNRDVTAKNH------------------- :  249
AtCAMTA1   : YDHLLNLKLEIAMVGHLLLACVMFHRFM------------------- :  312
AtCAMTA2   : SAHGNRVKGSNSQRSGDVPAWDASF---------------------- :  253
AtCAMTA3   : HQELQQPATGGNLTGFD-PYYQISLTPR------------------- :  245
AtCAMTA4   : FGQALKMLKEQLSIGDEHVNSVDPHYIQ------------------- :  248
AtCAMTA5   : AEDTSSGVHNTCNTGFEVRSNSLGSRNH------------------- :  200
AtCAMTA6   : SEQTFPNRVAAEDIDTVVRNHDISLH--------------------- :  188

                     *        440         *        460         *
NtabCAMTA1 : -----------PQSSVEYTSLSSIDGGGKCGRGNFASGPQRTIDLGS :  272
NtabCAMTA2 : -----------PQSSVEYTSLPGIDVGEKCGLGNFASGPQRTIDLGS :  222
NtabCAMTA3 : NKSRNTANTYIPSRNLDFPSWETISVNNPAAYQSYHFQPSSQSGANN :  311
NtabCAMTA4 : NKSGNTANTYIPSRNLDFPSWETTSVNNPAAYQSYHFQPSSQSGANN :  311
NtabCAMTA5 : NKSGNTANTYIPSRNLDFPSWETTSVNNPAAYQSYHFQPSSQSGANN :  298
NtabCAMTA6 : -----------FLPITSSFSTEKPTGLVEFSRDNFQLN--------- :  199
NtabCAMTA7 : -----------------ADAISDDSSLVEMQGS-------------- :  250
NtabCAMTA8 : -----------------ADAISDDSSLVEMQGN-------------- :  296
NtabCAMTA9 : ---------------SEIENSDDVENFVHDNNSLVQIQ--------- :  261
NtabCAMTA1 : ---------------SEIENSDDVENFGHDNSSLVQIQ--------- :  315
NtabCAMTA1 : -----------EQRLLEINTLEWDELLAPENPNKLNAT--------- :  328
NtabCAMTA1 : -----------EQRLLEINTLEWDELLAPDNPNKLIAT--------- :  197
NtabCAMTA1 : -----------EQRLLEINTLEWDELLAPDNPNKLIAT--------- :  276
AtCAMTA1   : -----------GTESEKMQPSNTDSMLVEEN---------------- :  332
AtCAMTA2   : -----------ENSLARYQNLPYNAPLTQTQPSTFGLI--------- :  280
AtCAMTA3   : -----------DSYQKELRTIPVTDSSIMVDKSKTINSP-------- :  273
AtCAMTA4   : -----------PESLDSLQFLEYSDIDHLAQPTTVYQRPENNKLE-- :  282
AtCAMTA5   : -----------EIRLHEINTLDWDELLVPADISNQSHP--------- :  227
AtCAMTA6   : ----------------DINTLDWDELLVPTDLNN------------- :  206
```

```
                            480         *        500         *
NtabCAMTA1 : QEPVSQHCSNGEMVCQDDFKNNLSVQRNWQYSFGDSASQFHGQIVNQ :  319
NtabCAMTA2 : QEPVSQHCSNGEIVCQDDFKNNLSVKGNWQYSFGDSASQFHGQIVNQ :  269
NtabCAMTA3 : MTHEQGSTTMGQVFLNDFKKQG-QNRIDSLG---DWQTSEGDAAFIS :  354
NtabCAMTA4 : MTHEQGNTKTGQVFLNDFKRQERQNRIDGLG---DWQTSEGDAAFIS :  355
NtabCAMTA5 : MTHEQGNTKTGQVFLNDFKRQERQNRIDGLG---DWQTSEGDAAFIS :  342
NtabCAMTA6 : -------PQFGSFVSIDAQSSDRNLNVTLQK------KFYSGYLNVA :  233
NtabCAMTA7 : ---------SNSLLLQHHSAESSESHHQHLT--------QDGHIWKD :  280
NtabCAMTA8 : ---------SNSLLLQHHSAESSESHHQHLT--------QDGHVWKD :  326
NtabCAMTA9 : -------HKSNNLLLQPHSGESSESQHQLLN--------LDGNIWKE :  293
NtabCAMTA1 : -------HKSNNLLLQPYSGESSESQHQLLN--------LDGDIWKE :  347
NtabCAMTA1 : -------QEAGG---RASAGQQNQFEVNGYS-------LNDGSLSVS :  358
NtabCAMTA1 : -------QEAGG---RASVGQQNQIEVNGYS-------LNDGSLSVS :  227
NtabCAMTA1 : -------QEAGG---RASVGQQNQIEVNGYS-------LNDGSLSVS :  306
AtCAMTA1   : -------SEKGGRLKAEHIRNPLQTQFNWQD-------DTDLALFEQ :  365
AtCAMTA2   : --PMEGKTEKGSLLTSEHLRNPLQSQVNWQT-------PVQESVPLQ :  318
AtCAMTA3   : -------GVTNGLKNRKSIDSQTWEEILGNC-----------GSGVE :  302
AtCAMTA4   : -------RCYGGNFGAQYSAKNDSNKLERCY---------GGYVGGA :  313
AtCAMTA5   : -------TEEDMLYFTEQLQTAPRGSVKQGN----HLAGYNGSVDIP :  263
AtCAMTA6   : -------QSAPTVDNLSYFTEPLQNAANGTA---------------- :  230

              520         *        540         *        560
NtabCAMTA1 : DLIGDS-----SHDLVNSFHNKNLSSDLYTGRGQSYLYPDEQEEQLT :  361
NtabCAMTA2 : DLIADS-----SYDLVNSFHNKNLSSDLYTGRGQSYLYPDEQEEQLT :  311
NtabCAMTA3 : KWSMDQKLNPNLASDHTIRSSAAYNVELHNSLEASHILPSHQDKHPM :  401
NtabCAMTA4 : KWSMDQKLHPDLASDHTIRSSAAYNVELHNSLEASHILPSHQDKHPM :  402
NtabCAMTA5 : KWSMDQKLHPDLASDHTIRSSAAYNVELHNSLEASHILPSHQDKHPM :  389
NtabCAMTA6 : DLLSSKLTYARLDGGRAVKDVANSRNRLTITSGEVLEENIHLAPAQI :  280
NtabCAMTA7 : -------------------------------------MLDHYGVSTA :  290
NtabCAMTA8 : -------------------------------------MLDHYGVSTA :  336
NtabCAMTA9 : -------------------------------------MLDHCRSFPA :  303
NtabCAMTA1 : -------------------------------------MLDHCRSFPA :  357
NtabCAMTA1 : RVPVAS--------------LESFV-CQVAGSDTVNFNPSNDTSFRS :  390
NtabCAMTA1 : RVPVAS--------------LESFV-CQVAGSDTVNFNPSNDMPFHS :  259
NtabCAMTA1 : RVPVAS--------------LESFV-CQVAGSDTVNFNPSNDMPFHS :  338
AtCAMTA1   : --------------------------SAQDNFETFSSLLGSENLQPF :  386
AtCAMTA2   : KWPMDS-----HSGMTDATDLALFGQGAHENFGTFSSLLGSQDQQSS :  360
AtCAMTA3   : ALPLQP------NSEHEVLDQILESSFTMQDFASLQESMVKSQNQEL :  343
AtCAMTA4   : EYHSSN--------------LMLVKNGSGPSGGTGGSGDQGSESWKD :  346
AtCAMTA5   : SFPGLE--------------DPVYQNNNSCGAGEFSSQHSHCGVDPN :  296
AtCAMTA6   : ----------------------------------------------- :    -
```

```
                            *        580          *        600          *
NtabCAMTA1  : QLNIQYLNSLVEVQGDFNQENSMDMLGLGDY---YTIKQPHLNSVKM :  405
NtabCAMTA2  : QLNIQYLNSLVEVQGDFNQENSMDMLGLGDY---STIKHPHLNSVKM :  355
NtabCAMTA3  : QNELPSQLSDANVGGSLNAELDHNL-SIGVRTDHSSLKQPLLDGVLR :  447
NtabCAMTA4  : QNELPSQLSDPNVGGSLNADLDHNL-SIGVRTDHSSLKQPLLDGVLR :  448
NtabCAMTA5  : QNELPSQLSDPNVGGSLNADLDHNL-SIGVRTDHSSLKQPLLDGVLR :  435
NtabCAMTA6  : QNISSSQTVVTPDAAVQNSSLEGRLNSDEAG---------------- :  311
NtabCAMTA7  : DESLNKSLPKLDENGMLQISSERGAIEAYQSYKWPNF---------- :  327
NtabCAMTA8  : AESLTKSLPKLDENGMLQISSERGAIEAYQSYKWPNF---------- :  373
NtabCAMTA9  : AESPAKCFEKLDENGTLQTSSGVGPIEATESDRWLKF---------- :  340
NtabCAMTA1  : AESQDKCFEKLDENGTLQTLSGMGPIEVTESDRWLKF---------- :  394
NtabCAMTA1  : GDGQMTSNFQKNESGVTTVGAGDSFDSLNKD---------------- :  421
NtabCAMTA1  : GDGQMTSNFRKNEPGVTTVGAGDSFDSLNKD---------------- :  290
NtabCAMTA1  : GDGQMTSNFRKNEPGVTTVGAGDSFDSLNKD---------------- :  369
AtCAMTA1    : GISYQAPPSNMDSEYMPVMKILRRSED-------------------- :  413
AtCAMTA2    : SFQAPFTN---NEAAYIPKLGPEDLIYEASANQTLPLRKALLKKED- :  403
AtCAMTA3    : NSGLTSDRTVWFQGQDMELNAISNLASNEKAPYLSTMKQHLLHGALG :  390
AtCAMTA4    : VLEACEASIPLNSEGSTPSSAKGLLAGLQEDSNWS------------ :  381
AtCAMTA5    : LQRRDFSATVTDQPGDALLNN-------------------------- :  317
AtCAMTA6    : ------------EHGNATVADG-SLDALLND---------------- :  248
                            g

                       620          *        640          *        6
NtabCAMTA1  : EEGLKKVDSFSRWVVKELEDV------EELHMQPTNRIS-WNVIDTL :  445
NtabCAMTA2  : EEGLKKVDSFSRWVVKELEDV------EELHMQRTNRIS-WNVIDTE :  395
NtabCAMTA3  : E-GLKKLDSFDRWMSKELEDV------SEPHMQSNSSSY-WDNVGDD :  486
NtabCAMTA4  : E-GLKKLDSFDRWMSKELEDV------SEPHMQSNSSSY-WDNVGDD :  487
NtabCAMTA5  : E-GLKKLDSFDRWMSKELEDV------SEPHMQSNSSSY-WDNVGDD :  474
NtabCAMTA6  : --SLKKLDSFGRWMDREIA-----VDGNESLLASDSGNY-WNTLDNG :  350
NtabCAMTA7  : --SEKEAQKAPIPAFKQLENFKYPAYSPGVTAFGSNSDQ-CTTIFDQ :  371
NtabCAMTA8  : --SEKEAQKAPIPAFKQLENFKYPAYSPGVTAFGSNSDQ-CTTIFDQ :  417
NtabCAMTA9  : --GGKALKSSL-TNFKQVEDFKY-PACARINTYGSYSDQ-YTTIFDQ :  382
NtabCAMTA1  : --GGKEALKSSLTNFKQVEDFKY-PACARINTYGSYSDQ-YTTIFDQ :  437
NtabCAMTA1  : --GLQTQDSFGRWINYFISDS---PGSADEMMTPESSVT-------- :  455
NtabCAMTA1  : --GLQTQDSFGRWINYFISDS---PGSADEMMTPESSVT-------- :  324
NtabCAMTA1  : --GLQTQDSFGRWINYFISDS---PGSADEMMTPESSVT-------- :  403
AtCAMTA1    : --SLKKVDSFSKWAIKELGEM------EDLQMQSSRGDIAWTTVECE :  452
AtCAMTA2    : --SLKKVDSFSRWVSKELGEM------EDLQMQSSSGGIAWTSVECE :  442
AtCAMTA3    : EEGLKKMDSFNRWMSKELGDVGVIADANESFTQSSSRTY-WEEVESE :  436
AtCAMTA4    : --YSNQVDQSTFLLPQDLGSFQLPASYSALVAPENNGEY-CGMMEDG :  425
AtCAMTA5    : --GYGSQDSFGRWVNNFISDS------PGSVDDPSLEAV-YTPGQDS :  355
AtCAMTA6    : --GPQSRESFGRWMNSFISES------NGSLEDPSFEPM-VMPRQDP :  286
                      s            6

              60        *          680          *        700
NtabCAMTA1  : D--DGSCLPTQLHVDSDSLNPSLSQEQVFSIIDFSPNWAYSNLETK- :  489
NtabCAMTA2  : D--DGSCLPTQLHVDSDSLNPSLSQEQVFSIIDFSPNWAYSNLETK- :  439
NtabCAMTA3  : DGVDNSTIASQVQLDTYMLSPSLSQDQFFSIIDFSPSWAFAGSEIK- :  532
NtabCAMTA4  : DGVDNSTIASQVQLDTYMLSPSLSQDQFFSIIDFSPSWAFAGSEIK- :  533
NtabCAMTA5  : DGVDNSTIASQVQLDTYMLSPSLSQDQFFSIIDFSPSWAFAGSEIK- :  520
NtabCAMTA6  : DKEVARL-SCHMQLDTNSLGPFLSQEQLFSISDFAPDWAYSGVETKV :  396
NtabCAMTA7  : DQIGTSL---------EDEMSLTISQKQKFTIRDISPDWGYSSEATK- :  409
NtabCAMTA8  : DQIGTSF---------EDEMSLTISQKQKFTFRDISPDWGYSSEATK- :  455
NtabCAMTA9  : DQIGTSF---------EDDMSLTIAQKQKFTIHDISPDWGYSSEATK- :  420
NtabCAMTA1  : DLIGTSF---------EDDMSLTIAQKQKFTFHDISPDWGYSSEATK- :  475
NtabCAMTA1  : -------------------IDQSYVMQQIFNITEISPTWALSSEETK- :  483
NtabCAMTA1  : -------------------IDQSYVMQQIFNITEISPTWALSSEETK- :  352
NtabCAMTA1  : -------------------IDQSYVMQQIFNITEISPTWALSSEETK- :  431
AtCAMTA1    : TAAAGIS------------LSPSLSEDQRFTIVDFWPKSAKTDAEVE- :  487
AtCAMTA2    : NAAAGSS------------LSPSLSEDQRFTMIDFWPKWTQTDSEVE- :  477
AtCAMTA3    : DGSNGHN--SRRDMDGYVMSPSLSKEQLFSINDFSPSWAYVGCEVV- :  480
AtCAMTA4    : MKIGLPF---------EQEMRVTGAHNQKFTIQDISPDWGYANETTK- :  463
AtCAMTA5    : STPPTVF------------HSHSDIPEQVFNITDVSPAWAYSTEKTK- :  390
AtCAMTA6    : LAPQAVF------------HSHSNIPEQVFNITDVSPAWAYSSEKTK- :  321
                                           Q F    d sP w      k
```

```
                      *       720         *       740         *
NtabCAMTA1  : ---------------------------------------VLITGRFL  :  497
NtabCAMTA2  : ---------------------------------------VLITGRFL  :  447
NtabCAMTA3  : ---------------------------------------VLITGKFL  :  540
NtabCAMTA4  : ---------------------------------------VLITGKFL  :  541
NtabCAMTA5  : ---------------------------------------VLITGKFL  :  528
NtabCAMTA6  : NLPDLIEWSVLIRNFENPRRHIVLVIDVKIQINGYISLQVLIIGTFL  :  443
NtabCAMTA7  : ---------------------------------------VVIIGSFL  :  417
NtabCAMTA8  : ---------------------------------------VVIIGSFL  :  463
NtabCAMTA9  : ---------------------------------------IVIVGSFL  :  428
NtabCAMTA1  : ---------------------------------------VMIVGSFL  :  483
NtabCAMTA1  : ---------------------------------------ILVIGHFP  :  491
NtabCAMTA1  : ---------------------------------------ILVIGHFP  :  360
NtabCAMTA1  : ---------------------------------------ILVIGHFP  :  439
AtCAMTA1    : ---------------------------------------VMVIGTFL  :  495
AtCAMTA2    : ---------------------------------------VMVIGTFL  :  485
AtCAMTA3    : ---------------------------------------VFVTGKFL  :  488
AtCAMTA4    : ---------------------------------------VIIIGSFL  :  471
AtCAMTA5    : [illegible]

                   760         *       780         *        80
NtabCAMTA1  : KSEGELIECKWSCMFGEIEVPAEVLADGVLRCHAPPHKPGVLPFYVT  :  544
NtabCAMTA2  : KSEGELIECKWSCMFGEVEVPAEVLADGVLRCHAPPHKPGVLPFYVT  :  494
NtabCAMTA3  : KSQPEVEK--WACMFGELEVPAEVIADGVLRCHTPNQKVGRVPFYIT  :  585
NtabCAMTA4  : KSQPEVEKCSWACMFGELEVPAEVIADGVLRCHTPIQKAGRVPFYIT  :  588
NtabCAMTA5  : KSQPEVEKCSWACMFGELEVPAEVIADGVLRCHTPIQKAGRVPFYIT  :  575
NtabCAMTA6  : GHGKHPTSQKWSCMFGEVEVSAELLTQSIIRCEVPSHSPGRVPFYVT  :  490
NtabCAMTA7  : CNPSE---CMWTCMFGDTEVPIQIIQEGVICCQAPPHLPGKVTLCVT  :  461
NtabCAMTA8  : CNPSE---CMWTCMFGDSEVPVQIIQEGVICCQAPPHLPGKVTLCVT  :  507
NtabCAMTA9  : CNPSE---YTWTCMFDDIEVPVQIINEGAIRCQAPPHLPCKVTLCVT  :  472
NtabCAMTA1  : CNPSE---YTWTCMFGDIEVPVQIIKEGAIRCQAPPHLPAEV-----  :  522
NtabCAMTA1  : GGQSQLAKSNLFCVCADVCFPAEFVQSGVYRCVISPQPPGLVNLYLS  :  538
NtabCAMTA1  : GAQSQLAKSNLFCVCADVCFPAEFVQSGVYRCVISPQPPGLVSLYLS  :  407
NtabCAMTA1  : GAQSQLAKSNLFCVCADVCFPAEFVQSGVYRCVISPQPPGLVSLYLS  :  486
AtCAMTA1    : LSPQEVTKYNWSCMFGEVEVPAEILVDGVLCCHAPPHTAGHVPFYVT  :  542
AtCAMTA2    : LSPQEVTSYSWSCMFGEVEVPADILVDGVLCCHAPPHEVGRVPFYIT  :  532
AtCAMTA3    : KTREETEIGEWSCMFGQTEVPADVISNGILQCVAPMHEAGRVPFYVT  :  535
AtCAMTA4    : CDPTE---STWSCMFGNAQVPFEIIKEGVIRCEAPQCGPGKVNLCIT  :  515
AtCAMTA5    : DSFQHLGRSNLICICGELRVPAEFLQMGVYRCFLPPQSPGVVNLYLS  :  445
AtCAMTA6    : DSYQHLERSNLYCVCGDFCVPAEYLQAGVYRCIIPPHSPGMVNLYLS  :  376
                          C6 g   vp   6  g   C  p    g 6

                0        *       820         *       840
NtabCAMTA1  : CSNRLACSEVREFEYRLGAYQ-----EFGAANV---S-ATEMHLLER  :  582
NtabCAMTA2  : CSNRLACSEVREFEYRLGAYQ-----EIGAANVS----ATEMHLLER  :  532
NtabCAMTA3  : CSNRLACSEVREFEFRVSESQ-----DVDV--ANSCS-SSESLLHMR  :  624
NtabCAMTA4  : CCNRLACSEVREFEFRVTEGQ-----DADVANANSCS-SSESLLHMR  :  629
NtabCAMTA5  : CCNRLACSEVREFEFRVTEGQ-----DADVANANSCS-SSESLLHMR  :  616
NtabCAMTA6  : CSNRLACSEVREFEYREKSSE-----LA----LALRP-SDEVRLQVR  :  527
NtabCAMTA7  : SGNRESCSEVKEFEYRVKPDDCARN-NRSDIEGAYKS-TEELLLLVR  :  506
NtabCAMTA8  : SGNRESCSEVKEFEYRDKPDDCARN-NRSDVEGAYKS-TEELLLLVR  :  552
NtabCAMTA9  : TGNRVSCSEVWEFEYRVKFDDHGQK-NLAEVGGACKS-SEELLLLVR  :  517
NtabCAMTA1  : ------------------------------GGACKS-SEELLHLVR  :  537
NtabCAMTA1  : FDGNTPISQVMTYEFRAPSAR-----KWTAPLEEQSS-WDEFRVQMR  :  579
NtabCAMTA1  : FDGNTPISQVMTYEFRAPSAC-----KWTAPLEEQSS-WDEFRVQMR  :  448
NtabCAMTA1  : FDGNTPISQVMTYEFRAPSAC-----KWTAPLEEQSS-WDEFRVQMR  :  527
AtCAMTA1    : CSNRFACSEVREFDFLSGSTQ-----KINATDVYGTY-TNEASLQLR  :  583
AtCAMTA2    : CSDRFSCSEVREFDFLPGSTR-----KLNATDIYGAN-TIETSLHLR  :  573
AtCAMTA3    : CSNRLACSEVREFEYKVAESQ-----VFDREADDEST-ID--ILEAR  :  574
AtCAMTA4    : SGDGLLCSEIREFEYREKPDTCCPKCSEPQTSDMSTS-PNELILLVR  :  561
AtCAMTA5    : VDGNKPISQLFSFEHRSVQFI-----EKAIPQDDQLYKWEEFEFQVR  :  487
AtCAMTA6    : ADGHKPISQCFRFEHRAVPVL-----DKTVPEDNQDSKWEEFEFQVR  :  418
                     s     e                            e    R
```

```
                   *        860         *        880         *
NtabCAMTA1 : IESLLSLEP-LSSCHSSDSMEAAKEKQSTVNRIICMMEEE---NQQM :  625
NtabCAMTA2 : IESLMSLGP-VSSCHSSDSMEAAKEKHSTVNKIICMMEEE---NQQM :  575
NtabCAMTA3 : FGKLLSLESTVSLSSPPRSEDDVSNVCSKINSLLKEDDNE---WEEM :  668
NtabCAMTA4 : FGKLLSLESTVSLSSPPRSEDDVSHVCSKINSLLNEDDNE---WEEM :  673
NtabCAMTA5 : FGKLLSLESTVSLSSPPRSEDDVSHVCSKINSLLNEDDNE---WEEM :  660
NtabCAMTA6 : LAKLLYSGLNKKFLDCSSTDCERGKLKTLLCSLKCNIGNASESLEDL :  574
NtabCAMTA7 : FVQMLLLDLSVHKEDSSELSND-------FLEKSKANEDS---WSQV :  543
NtabCAMTA8 : FVQLLLLDLSAQKEDSSMLSND-------FLEKCKANEDS---WSQV :  589
NtabCAMTA9 : FVQMLLSDSSVQKGDGSGSSND-------ILENSKASEDS---WSQV :  554
NtabCAMTA1 : FVQMLLSDSSVQKGDGSGSSND-------ILENSKASEDS---WSQV :  574
NtabCAMTA1 : LAHLLFSTSKSLSIFSSKVHQDSLKEAKRFVRKCSHITDN---WAYL :  623
NtabCAMTA1 : LAHLLFSTSKSLSIFSSKVHQDSLKEAKRFVRKCSHITDN---WAYL :  492
NtabCAMTA1 : LAHLLFSTSKSLSIFSSKVHQDSLKEAKRFVRKCSHITDN---WAYL :  571
AtCAMTA1   : FEKMLAHRDFVHEHHIFEDVGD----KRRQISKIMLLKEE---KEYL :  623
AtCAMTA2   : FENLLALRCSVQEHHIFENVGE----KRRKISKIMLLKDE---KEPP :  613
AtCAMTA3   : FVKLLCSKSENTS-PVSGNDSDLSQLSEKISLLLFENDDQ---LDQM :  617
AtCAMTA4   : FVQTLLSDRSSERKSNLESGND------KLLTKLKADDDQ---WRHV :  599
AtCAMTA5   : LAHLLFTSSNKISVLTSKISPENLLEAKKLASRTSHLLNS---WAYL :  531
AtCAMTA6   : LSHLLFTSSNKLNVLSSKISPHNLRDAKKLASKTNHLLNS---WAYL :  462
               6

               900         *        920         *        940
NtabCAMTA1 : IERASDHDTSQCGVKEDLFLERKLKQNFYAWLVRQVT-DDGRGRTAI :  671
NtabCAMTA2 : IERASDYDTSQCGVKEDLFLERKLKQNFYAWLVRQVT-DDGRGRTAI :  621
NtabCAMTA3 : LNLTYENNFMAEKVKDQLL-QKLLKEKLRVWLLQKVA-EGGKGPNVL :  713
NtabCAMTA4 : LNLTYENNFMAEKVKDQLL-QKLLKEKLRVWLLQKVA-EGGKGPNVL :  718
NtabCAMTA5 : LNLTYENNFMAEKVKDQLL-QKLLKEKLRVWLLQKVA-EGGKGPNVL :  705
NtabCAMTA6 : LAIIEGNHI---NFRDTLI-QSFMKDKFYEWLVSRAH-EEDKGPNIL :  616
NtabCAMTA7 : IESLLFGTSTSTITIDWLL-QELLKDKFQQWLSYKLQRKDNQMGCSL :  589
NtabCAMTA8 : IESLLFGTSTSTITIDWLL-QELLKDKFQQWLSYKLQRKDNQMGCSL :  635
NtabCAMTA9 : IESLLFGTSTSMVTVDWLL-QELLKDRLKQWLSSKLQVKNNQMGYSF :  600
NtabCAMTA1 : IESLLFGTSTSMVTVDWLL-QELLKDKLQQWLSSKLQVQNNQMGYSF :  620
NtabCAMTA1 : IKSIEDRKLPVPHAKDCLF-ELSLQTKFHEWLLERV--IGGCKTSEW :  667
NtabCAMTA1 : IKSIEDRKLPVPHAKDCLF-ELSLQTKFHEWLLERV--IGGCKTSEW :  536
NtabCAMTA1 : IKSIEDRKLPVPHAKDCLF-ELSLQTKFHEWLLERV--IGGCKTSEW :  615
AtCAMTA1   : LPGTYQRDSTKQEPKGQLF-RELFEEELYIWLIHKVT-EEGKGPNIL :  668
AtCAMTA2   : LPGTIEKDLTELEAKERLI-REEFEDKLYLWLIHKVT-EEGKGPNIL :  658
AtCAMTA3   : L----MNEISQENMKNNLL-QEFLKESLHSWLLQKIA-EGGKGPSVL :  658
AtCAMTA4   : IGTIIDGSASSTSTVDWLL-QELLKDKLDTWLSSRSC-DEDYITCSL :  644
AtCAMTA5   : MKSIQANEVPFDQARDHLF-ELTLKNRLKEWLLEKV--IENRNTKEY :  575
AtCAMTA6   : VKSIQGNKVSFDQAKDHLF-ELSLKNRLKEWLMEKV--LEGRNTLDY :  506
             6                  L             WL
NtabCAMTA5 : DEGGQGVLHFAAALGYDWAIPPTIAAGVSVNFRDVNGWTALHWAASY :  752
NtabCAMTA6 : DDEGQGVIHLVASLGYEWGLVLLTAAGINPNLRDARGRTALHWAAHY :  663
NtabCAMTA7 : SKKEQGIIHMVSGLGFEWALHPILNAGVSVDFRDINGWTALHWAARF :  636
NtabCAMTA8 : SKKEQGIIHMVSGLGFEWALHPILNAAVSVNFRDINGWTALHWAARF :  682
NtabCAMTA9 : SRKEQGIIHMVAGLGFEWALHPILDAGVGVNFRDINGWTALHWAARF :  647
NtabCAMTA1 : SRKEQGIIHMVAVLGFEWALQPILDAGVSVNFRDINGWTALHWAARF :  667
NtabCAMTA1 : DEQGQGVIHLCAILGYTWAVYPFSWSGLSLDYRDKYGWTALHWAAHY :  714
NtabCAMTA1 : DEQGQGVIHLCAILGYTWAVYPFSWSGLSLDYRDKYGWTALHWAAHY :  583
NtabCAMTA1 : DEQGQGVIHLCAILGYTWAVYPFSWSGLSLDYRDKYGWTALHWAAHY :  662
AtCAMTA1   : DEDGQGILHFVAALGYDWAIKPVLAAGVNINFRDANGWSALHWAAFS :  715
AtCAMTA2   : DEDGQGVLHLAAALGYDWAIKPILAAGVSINFRDANGWSALHWAAFS :  705
AtCAMTA3   : DEGGQGVLHFAASLGYNWALEPTIIAGVSVDFRDVNGWTALHWAAFF :  705
AtCAMTA4   : SKQEQGIIHMVAGLGFEWAFYPILAHGVNVDFRDIKGWSALHWAAQF :  691
AtCAMTA5   : DSKGLGVIHLCAVLGYTWSILLFSWANISLDFRDKQGWTALHWAAYY :  622
AtCAMTA6   : DSKGLGVIHLCASLGYTWSVQLFSLSGLSLNFRDKQGWTALHWAAYY :  553
                 qG66H  a LG5 Wa  p     g6s 1 RD  Gw3ALHWAA
```

```
                *      1000        *       1020        *
NtabCAMTA1 : GREKTVVGL-VSLGASPGALTDPSAEFPLGRTPADLASANGHKGISG :  764
NtabCAMTA2 : GREKTVVGL-VSLGASPGALTDPSAEFPLGRTPADLASANGHKGISG :  714
NtabCAMTA3 : GRERTVGFLIISLGAAPGALTDPTPKHPSGRTPADLASSNGHKGIAG :  807
NtabCAMTA4 : GRERTVGFL-ISLGAAPGALTDPTPKHPSGRTPADLASSNGHKGIAG :  811
NtabCAMTA5 : GRERTVGFL-ISLGAAPGALTDPTPKHPSGRTPADLASSNGHKGIAG :  798
NtabCAMTA6 : GREDMVIAL-VKLGVAVCAVDDPTAAFPGGQTAADLASSGGHKGVAG :  709
NtabCAMTA7 : GREQMVASL-IASGASAGAVTDPSPRDPVGKTAASIASSCGHKGLAG :  682
NtabCAMTA8 : GREKMVASL-IASGASAGAVTDPSSRDPVGKTAASIASSCGHKGLAG :  728
NtabCAMTA9 : GREKMVASL-VASSAFAGAVTDPSSQDPFGRTAASIASSCGHKGVAG :  693
NtabCAMTA1 : GREKMVASL-VASGAFAGAVTDPSSQDPFGKTAASIASSCGHKGVAG :  713
NtabCAMTA1 : GREKMVATL-LSASAKPNLVTDPTSENPGGSTAADLASKNGFEGLGA :  760
NtabCAMTA1 : GREKMVATL-LSAGAKPNLVTDPTSENPGGSTAADLASKNGFEGLGA :  629
NtabCAMTA1 : GREKMVATL-LSAGAKPNLVTDPTSENPGGSTAADLASKNGFEGLGA :  708
AtCAMTA1   : GREETVAVL-VSLGADAGALTDPSPELPLGKTAADLAYANGHRGISG :  761
AtCAMTA2   : GREDTVAVL-VSLGADAGALADPSPEHPLGKTAADLAYGNGHRGISG :  751
AtCAMTA3   : GRERIIGSL-IALGAAPGTLTDPNPDFPSGSTPSDLAYANGHKGIAG :  751
AtCAMTA4   : GSEKMVAAL-IASGASAGAVTDPSRQDPNGKTAASIAASNGHKGLAG :  737
AtCAMTA5   : GREKMVAAL-LSAGARPNLVTDPTKEFLGGCTAADLAQQKGYDGLAA :  668
AtCAMTA6   : GREKMVAAL-LSAGARPNLVTDSTKDNLGGCMAADLAQQNGYDGLAA :  599
             GrE  6  L 6  ga    6tDp    p G t a 6A   G  G6

               1040        *       1060        *       1080
NtabCAMTA1 : FLAESSLTTHLSKLTVTDAKEELASEVSGAKVGETVTERVAVTTTGD :  811
NtabCAMTA2 : FLAESSLTTHLSKLTVTDATEELASEVSGAKVGETVTERVAVTTTGD :  761
NtabCAMTA3 : YLAESSLSSHLSSLELKEMKQGE-TVQPFGEAVQTVSERSATPAWDG :  853
NtabCAMTA4 : YLAESSLSFHLSSLELKEMKQGE-NVQPFGEAVQTVSERSATPAWDG :  857
NtabCAMTA5 : YLAESSLSFHLSSLELKEMKQGE-NVQPFGEAVQTVSERSATPAWDG :  844
NtabCAMTA6 : YLAESELTAHLQSLAINNNALDSICAGLEAEKAFESAAQEVVPLNGT :  756
NtabCAMTA7 : YLSEVALTSHLSSLTLVESELSKGTADVEAEKTISSISNTSATTN-- :  727
NtabCAMTA8 : YLSEVALTSHLSSLTLEESELSKGTADVEAEKTISSISNTSATTN-- :  773
NtabCAMTA9 : YLSEVALTSHLSSLTLEENELSKGTADVEAERTISSISTTSAATH-- :  738
NtabCAMTA1 : YLSEVALTSHLSSLTLEESELSKGAADVEAERTISSISTTNAATH-- :  758
NtabCAMTA1 : YLAEKALVAHFKDMTL--------AGNVSGSLQTTTEH--INPGNFT :  797
NtabCAMTA1 : YLAEKALVAHFKDMTL--------AGNVSGSLQTTTEH--INPGNFT :  666
NtabCAMTA1 : YLAEKALVAHFKDMTL--------AGNVSGSLQTTTEH--INPGNFT :  745
AtCAMTA1   : FLAESSLTSYLEKLTVDSKENSP-ANSCGEKAVQTVSERTAAPMTYG :  807
AtCAMTA2   : FLAESSLTSYLEKLTVDAKENSS-ADSSGAKAVLTVAERTATPMSYG :  797
AtCAMTA3   : YLSEYALRAHVSLLSLNDKN----AETVE-----------MAPS--- :  780
AtCAMTA4   : YLSEVALTNHLSSLTLEETENSKDTAQVQTEKTLNSISEQSPSGN-- :  782
AtCAMTA5   : FLAEKCLVAQFKDMQT--------AGNISGNLETIKAEKSSNPGNAN :  707
AtCAMTA6   : YLAEKCLVAQFRDMKI--------AGNITGDLEACKAE-MLNQGTLP :  637
             5L E  L  h   6

                     *       1100        *       1120
NtabCAMTA1 : DMPDVLSLKDSLAAIRNATQAAARIHQIFRVQSFQRKQIIE------ :  852
NtabCAMTA2 : DVPDVLSLKDSLAAIRNATQAAARIHQIFRVQSFQRKQIIE------ :  802
NtabCAMTA3 : DWPHGVSLKDSLAAVRNATQAAARIHQVFRVQSFQRKQLKE------ :  894
NtabCAMTA4 : DWPHGVSLKDSLAAVRNATQAAARIHQVFRVQSFQRKQLKE------ :  898
NtabCAMTA5 : DWPHGVSLKDSLAAVRNATQAAARIHQVFRVQSFQRKQLKE------ :  885
NtabCAMTA6 : -IHDDISLKGSLASVRKSAHAAALIQAAFRARSFHQRQLRE------ :  796
NtabCAMTA7 : --EDQRSLKDSLAAVRNAAQAAARIQSAFRAHSFRKRQQRESAIATT :  772
NtabCAMTA8 : --EDQRSLKDSLAAVRNAAQAAARIQSAFRAHSFRKRQQRESAVTAT :  818
NtabCAMTA9 : --EDQLSLKDTLAAVRNAAQAAARIQSAFRAHSFRKRRQREAARAAT :  783
NtabCAMTA1 : --EDQLSLKDTLAAVRNAAQAAARIQSAFRAHSFRKRRQREAARAAT :  803
NtabCAMTA1 : --EEELYLKDTLAAYRTAADAAARIQAAFREHSFK-VQTKA------ :  835
NtabCAMTA1 : --EEELYLKDTLAAYRTAADAAARIQAAFREHSFK-VQTKA------ :  704
NtabCAMTA1 : --EEELYLKDTLAAYRTAADAAARIQAAFREHSFK-VQTKA------ :  783
AtCAMTA1   : DVPEKLSLKDSLTAVRNATQAADRLHQVFRMQSFQRKQLCD-----I :  849
AtCAMTA2   : DVPETLSMKDSLTAVLNATQAADRLHQVFRMQSFQRKQLSE-----L :  839
AtCAMTA3   : --PSSSSLTDSLTAVRNATQAAARIHQVFRAQSFQKKQLKE------ :  819
AtCAMTA4   : --EDQVSLKDTLAAVRNAAQAAARIQAAFRAHSFRKRKQRE--AALV :  825
AtCAMTA5   : --EEEQSLKDTLAAYRTAAEAAARIQGAFREHELK-VRSSA------ :  745
AtCAMTA6   : --EDEQSLKDALAAYRTAAEAAARIQGAFREKALKAARSSV------ :  676
                   s6kd Laa r a  AAar6    FR  sf
```

```
                       *       1140            *       1160            *
NtabCAMTA1 : CSDNELS-SDENALSIVASRACKLGQ--NNGIAH----AAATQIQKK :  892
NtabCAMTA2 : RSDNELS-SDENALSIVASRACKLGQ--NNGIAH----AAATQIQKK :  842
NtabCAMTA3 : HGGSEFGLSDEHALSLLALKTNKAGQ--HDEPVH----TAAVRIQNK :  935
NtabCAMTA4 : HGGSEFGLSDEHALSLLALKTNKAGQ--HDEPVH----TAAVRIQNK :  939
NtabCAMTA5 : HGGSEFGLSDEHALSLLALKTNKAGQ--HDEPVH----TAAVRIQNK :  926
NtabCAMTA6 : -SRNDVSEASVDLVALGSLNKVQKVNH-FEDYLH----PAAIKIQQK :  837
NtabCAMTA7 : ASGDEYGILSNDILGLSAASKWAFRN--TRDYNS-----AALAIQKK :  812
NtabCAMTA8 : ASGDEYGILSNNIHGLSAASKWAFRN--TRDYNS-----AALAIQKK :  858
NtabCAMTA9 : TSGDEYCVLSNDVLGLSAASKLAFRN--MRDYNS-----AALAIQKK :  823
NtabCAMTA1 : TSGDEYCVLSNDVLGLSAASKLAFRN--MRDYNS-----AALAIQKK :  843
NtabCAMTA1 : ---VESSNPEIEARNIVAAMKIQHA---FRNYESRKKLAAAARIQYR :  876
NtabCAMTA1 : ---VESSNPEMEARNIVAAMKIQHA---FRNYESRKKLAAAARIQYR :  745
NtabCAMTA1 : ---VESSNPEMEARNIVAAMKIQHA---FRNYESRKKLAAAARIQYR :  824
AtCAMTA1   : GDDEKIDISDQLAVSFAASKTKNPGQ--GDVSLS----CAATHIQKK :  890
AtCAMTA2   : GGDNKFDISDELAVSFAAAKTKKSGH--SSGAVH----AAAVQIQKK :  880
AtCAMTA3   : FGDKKLGMSEERALSMLAPKTHKSGRAHSDDSVQ----AAAIRIQNK :  862
AtCAMTA4   : ACLQEYGMYCEDIEGISAMSKLTFGK--GRNYNS-----AALSIQKN :  865
AtCAMTA5   : ---VRFASKEEEAKNIIAAMKIQHA---FRNFEVRRKIAAAARIQYR :  786
AtCAMTA6   : ---IQFANKEEEAKSIIAAMKIQNA---FRKYDTRRKIEAAYRIQCR :  717
                                 a                    AA  IQ
```

```
               1180          *      1200          *       1220
NtabCAMTA1 : FRGWNKRRKEFLLIRQKIVKIQAHVRGHQVRKKYKPIIWSVGILEKVI :  939
NtabCAMTA2 : FRGWNKRRKEFLLIRQKIVKIQAHVRGHQVRKKYKPIIWSVGILEKVI :  889
NtabCAMTA3 : FRSWKGRRDYLLIRQRIIKIQAHVRGHQVRNKYKNIIWSVGILEKVI :  982
NtabCAMTA4 : FRSWKGRRDYLLIRQRIIKIQAHVRGHQVRNKYKNIIWSVGILEKVI :  986
NtabCAMTA5 : FRSWKGRRDYLLIRQRIIKIQAHVRGHQVRNKYKNIIWSVGILEKVI :  973
NtabCAMTA6 : YRGWKGRREFLKIRNRIVKIQAHVRGHQVRKQYKKFVWSVSIVEKAI :  884
NtabCAMTA7 : YRGWKGRKDFLAFRQKVVKIQAHVRGYQVRKQYK-VCWAVGILEKVV :  858
NtabCAMTA8 : YRGWKGRKDFLAFRQKVVKIQAHVRGYQVRKQYK-VCWAVGILEKVV :  904
NtabCAMTA9 : YRGWKCRKDFLAFRQKVVKIQAHVRGYQVRKEYK-VCWAVGILEKVV :  869
NtabCAMTA1 : YRGWKCRKDFLAFRQKVVKIQAHVRGYQVRKEYK-VCWAVGILEKVV :  889
NtabCAMTA1 : FRSWKMRKDFLNMRRHAIKIQAVFRGFQVRKQYRKIVWSVGVLEKAV :  923
NtabCAMTA1 : FRSWKMRKDFLNMRRHAIKIQAVFRGFQVRKQYRKIVWSVGVLEKAV :  792
NtabCAMTA1 : FRSWKMRKDFLNMRRHAIKIQAVFRGFQVRKQYRKIVWSVGVLEKAV :  871
AtCAMTA1   : YRGWKKRKEFLLIRQRIVKIQAHVRGHQVRKQYRTVIWSVGLLEKII :  937
AtCAMTA2   : YRGWKKRKEFLLIRQRIVKIQAHVRGHQVRKQYRAIIWSVGLLEKII :  927
AtCAMTA3   : FRGYKGRKDYLITRQRIIKIQAHVRGYQFRKNYRKIIWSVGVLEKVI :  909
AtCAMTA4   : FRGYKDRKCFLELRQKVVKIQAHVRGYQIRKNYKVICWAVRILDKVV :  912
AtCAMTA5   : FQTWKMRREFLNMRKKAIRIQAAFRGFQVRRQYQKITWSVGVLEKAI :  833
AtCAMTA6   : FQTWKIRREYLNMRRQAIRIQAAFRGLQARRQYKKILWSVGVLEKAV :  764
             5r 5k R4 5L  R   64IQA  RG Q R  Y    W Vg66eK 6
```

```
                   *        1240          *       1260
NtabCAMTA1 : LRWRRKRSGLRGFRSEVVMNKPI-------IQD---DSLPEDDYDFL :  976
NtabCAMTA2 : LRWRRKRSGLRGFRSEVVINKPS-------IQD---DSLPEDDYDFL :  926
NtabCAMTA3 : LRWRRKGSGLRGFKPEATLTEGSN------MQD---RPVQEDDYDFL : 1020
NtabCAMTA4 : LRWRRKGSGLRGFKPEATLTEGSD------TQD---RPVQEDDYDFL : 1024
NtabCAMTA5 : LRWRRKGSGLRGFKPEATLTEGSN------MQD---RPVQEDDYDFL : 1011
NtabCAMTA6 : LRWRRKKPGLRGFQPEKTSQK---------ELP---EFEKNDEYEYL :  919
NtabCAMTA7 : LRWRRRGVGLRGFRHDA---------------E---SIDESEDEDIL :  887
NtabCAMTA8 : LRWRRRGVGLRGFRHDT---------------E---SIDESEDEDIL :  933
NtabCAMTA9 : LRWRRRGVGLRGFRLEE---------------E---PIEESEDEDIL :  898
NtabCAMTA1 : LRWRRRGVGLRGFRLEE---------------E---PIEESEDEDIL :  918
NtabCAMTA1 : LRWRLKRKGFRGLQVQS-------------SQAVDIKPDGDVEEDFF :  957
NtabCAMTA1 : LRWRLKRKGFRGLQVQS-------------SQAVDIKPDGDVEEDFF :  826
NtabCAMTA1 : LRWRLKRKGFRGLQVQS-------------SQAVDIKPDGDVEEDFF :  905
AtCAMTA1   : LRWRRKGNGLRGFKRNAVAKTVEPEPPVSAICP---RIPQEDEYDYL :  981
AtCAMTA2   : LRWRRKGSGLRGFKRDTISK----------PTEPVCPAPQEDDYDFL :  964
AtCAMTA3   : LRWRRKGAGLRGFKSEALVEK---------MQD---GTEKEEDDDFF :  944
AtCAMTA4   : LRWRRKGVGLRGFRQDV---------------E---STEDSEDEDIL :  941
AtCAMTA5   : LRWRLKRKGFRGLQVSQ-------------PDE---KEGSEAVEDFY :  864
AtCAMTA6   : LRWRQKRKGFRGLQVAA--------------EE---DSPGEAQEDFY :  794
             LRWR 4   G RG                                d
```

```
                   *        1280            *        1300            *
NtabCAMTA1 : KEGRKHT-EVRMQKALARVKSMTQYPEGRAQYRRLLTAAEGLREVKP : 1022
NtabCAMTA2 : KEGRKQT-EVRMQKALARVKSMTQYPEGRAQYRRLLTAAEGLREV-K :  971
NtabCAMTA3 : KEGRKQT-EQRLQKALARVKSMVQYPEARDQYRRLLNVVSDMKDT-T : 1065
NtabCAMTA4 : KEGRKQT-EQRLQKALARVKSMVQYPEARDQYRRLLNVVSDMKDT-T : 1069
NtabCAMTA5 : KEGRKQT-EQRLQKALARVKSMVQYPEARDQYRRLLNVVSDMKDT-T : 1056
NtabCAMTA6 : SIGRKQK-FAGVQKALARVQSMVRHPEARDQYMRLVAKFDSFKELMA :  965
NtabCAMTA7 : KVFRKQKVDAALDEAVSRVLSMVESPGARQQYHRILEKYRQAKAE-- :  932
NtabCAMTA8 : KVFRKQKVDAALDEAVSRVLSMVESPGARQQYHRILEKYRQAKAE-- :  978
NtabCAMTA9 : KLFRKQKVDAAINEAVSRVLSMVDSPEARQQYHRILEKYRQAKGKPT :  945
NtabCAMTA1 : KLFRKQKVDAAINEAVSRVLSMVDSPEARQQYHRILEKYRQAKAE-- :  963
NtabCAMTA1 : RASRKQA-EERVERSVVRVQAMFRSKRAQEEYRRMKLEHDNATLE-- : 1001
NtabCAMTA1 : RASRKQA-EERVERSVVRVQAMFRSKRAQEEYRRMKLEHDNATLE-- :  870
NtabCAMTA1 : RASRKQA-EERVERSVVRVQAMFRSKRAQEEYRRMKLEHDNATLE-- :  949
AtCAMTA1   : KEGRKQT-EERLQKALTRVKSMVQYPEARDQYRRLLTVVEGFRENEA : 1027
AtCAMTA2   : KEGRKQT-EERLQKALTRVKSMAQYPEARAQYRRLLTVVEGFRENEA : 1010
AtCAMTA3   : KQGRKQT-EDRLQKALARVKSMVQYPEARDQYRRLLNVVNDIQESKV :  990
AtCAMTA4   : KVFRKQKVDVAVNEAFSRVLSMSNSPEARQQYHRVLKRYCQTKAELG :  988
AtCAMTA5   : KTSQKQA-EERLERSVVKVQAMFRSKKAQQDYRRMKLAHEEAQLE-- :  908
AtCAMTA6   : KTSQRQA-EERLERSVVRVQAMFRSKKAQQDYRRMKLTHEEAQLE-- :  838
                  r4q     6      4V  M      a    Y R6
[illegible]
NtabCAMTA2 : DGSTCIQESSEDTSYP------------------------------- :  987
NtabCAMTA3 : TTSDGAPSNSGEAADF------------------------------- : 1081
NtabCAMTA4 : TTSDGAPSNSVEAADF------------------------------- : 1085
NtabCAMTA5 : TTSDGAPSNSVEAADF------------------------------- : 1072
NtabCAMTA6 : KAKCGKLYSYCAGSHM------------------------------- :  981
NtabCAMTA7 : --LEGAESETASTA--------------------------------- :  944
NtabCAMTA8 : --LEGAESESASTA--------------------------------- :  990
NtabCAMTA9 : DGTTGRSNGRSSSTTKHAKPGRNARGRGGGGHHAIVGGTISGIFKPK :  992
NtabCAMTA1 : ---LGVNSDTVSTA--------------------------------- :  974
NtabCAMTA1 : ----------------------------------------------- :    -
NtabCAMTA1 : ----------------------------------------------- :    -
NtabCAMTA1 : ----------------------------------------------- :    -
AtCAMTA1   : SSSASINNKEEEAVNC------------------------------- : 1043
AtCAMTA2   : SSSSALKNNTEEAANYN------------------------------ : 1027
AtCAMTA3   : EKALENSEATCFDD--------------------------------- : 1004
AtCAMTA4   : KTETLVGED-------------------------------------- :  997
AtCAMTA5   : ----------------------------------------------- :    -
AtCAMTA6   : ----------------------------------------------- :    -

                   *      1380            *       1400            *
NtabCAMTA1 : --------EEELFDVENLL-DDDTFMSIAFE---------------- : 1060
NtabCAMTA2 : --------EEELFDVENLL-DDDTFMSIAFE---------------- : 1009
NtabCAMTA3 : --------GDDLIDLDDLL-DDDTFMSTAP----------------- : 1102
NtabCAMTA4 : --------GDDLIDLDDLL-DDDTFMSTAP----------------- : 1106
NtabCAMTA5 : --------GDDLIDLDDLL-DDDTFMSTAP----------------- : 1093
NtabCAMTA6 : -------EGCELLHLQGRVVGGGISKEHGPISCSKD----------- : 1010
NtabCAMTA7 : --------HGDMSNME----NDDIYQFPSY----------------- :  962
NtabCAMTA8 : --------HGDMSNME----NDDIYQFSSY----------------- : 1008
NtabCAMTA9 : RGKNTNIQGEARQKIYLQLSSDTLEVLTGHKTSTCPYSGEECGDIGF : 1039
NtabCAMTA1 : --------HGDIS-------NSDI----------------------- :  983
NtabCAMTA1 : ------------YERASVL-NPDIQIG-------------------- : 1015
NtabCAMTA1 : ------------YERASLL-NPDIQIG-------------------- :  884
NtabCAMTA1 : ------------YERASLL-NPDIQIG-------------------- :  963
AtCAMTA1   : -------EEDDFIDIESLL-NDDTLMMSISP---------------- : 1066
AtCAMTA2   : -------EEDDLIDIDSLL-DDDTFMSLAFE---------------- : 1050
AtCAMTA3   : --------DDDLIDIEALLEDDDTLMLPMSSSLWTS----------- : 1032
AtCAMTA4   : --------DDGLFDIADME-YDTLFSLP------------------- : 1016
AtCAMTA5   : --------YDGMQELDQMATEES------------------------ :  923
AtCAMTA6   : --------YGCLEDI-------------------------------- :  845
```

```
                  1420
NtabCAMTA1 : ------------ :    -
NtabCAMTA2 : ------------ :    -
NtabCAMTA3 : ------------ :    -
NtabCAMTA4 : ------------ :    -
NtabCAMTA5 : ------------ :    -
NtabCAMTA6 : ------------ :    -
NtabCAMTA7 : ------------ :    -
NtabCAMTA8 : ------------ :    -
NtabCAMTA9 : IPKEKPQQQPKQ : 1051
NtabCAMTA1 : ------------ :    -
NtabCAMTA1 : ------------ :    -
NtabCAMTA1 : ------------ :    -
NtabCAMTA1 : ------------ :    -
AtCAMTA1   : ------------ :    -
AtCAMTA2   : ------------ :    -
AtCAMTA3   : ------------ :    -
AtCAMTA4   : ------------ :    -
AtCAMTA5   : ------------ :    -
AtCAMTA6   : ------------ :    -
```

Supplementary Figure S5. Phylogenetic tree showing relationships of CAMTAs between *N. tabacum* and *A. thaliana*. The multiple sequence alignment of 13 putative NtabCAMTAs and 6 AtCAMTAs was performed by ClustalX program. MEGA 6.0 was used to create the unrooted maximum likelihood (ML) tree under the Jones-Taylor-Thornton (JTT) model. The bootstrap values from 1000 resampling are given at each node. The NtabCAMTAs identified in this study are shown in red diamonds and AtCAMTAs are shown in pink circles. Each group is highlighted in different color.

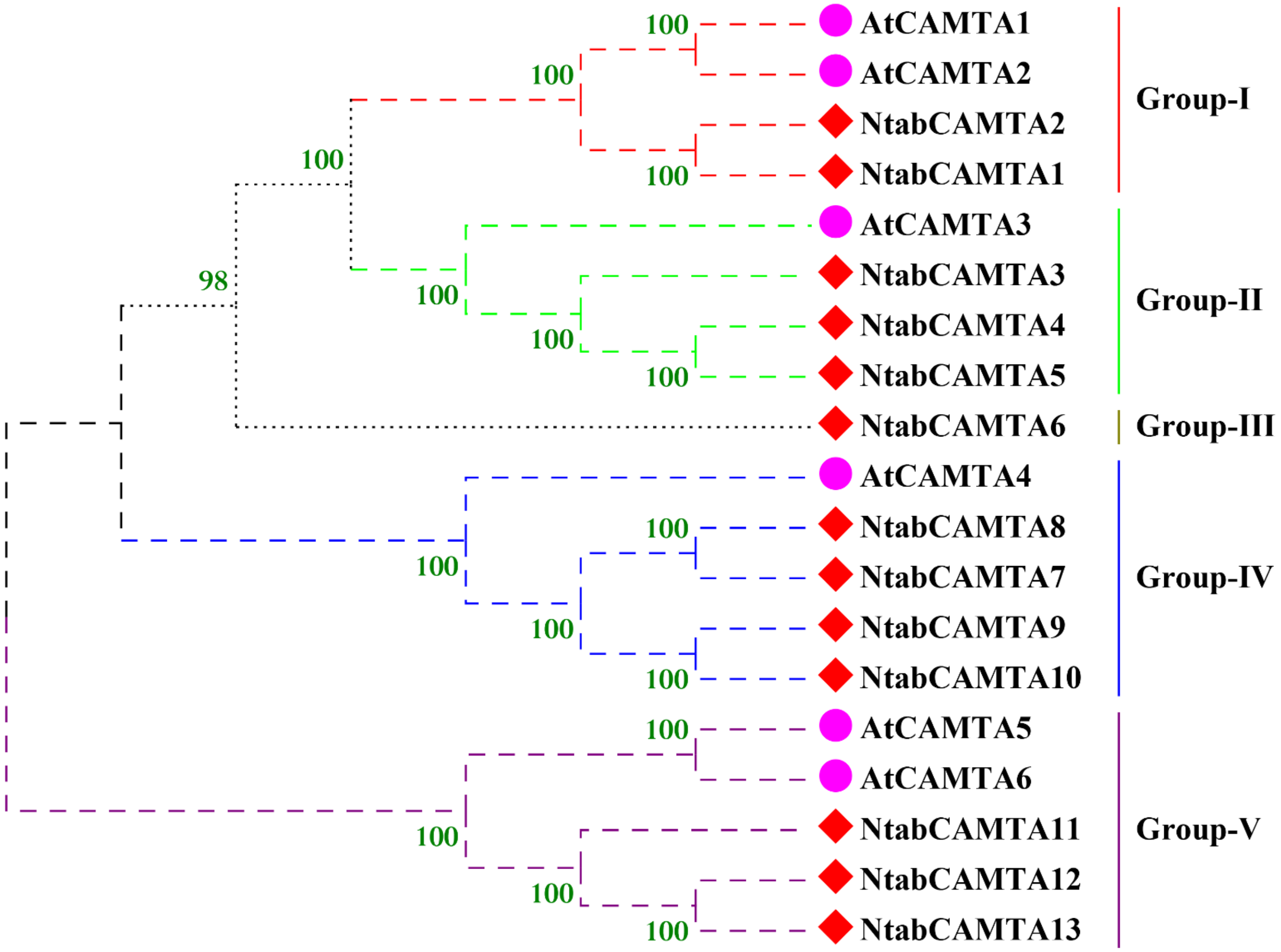

Supplementary Figure S6. Molecular Phylogenetic analysis of NtabCAMTA, NtomCAMTA and NsylCAMTA family proteins. The evolutionary history was inferred by using the Maximum Likelihood method based on the JTT matrix-based model. The tree with the highest log likelihood (-7958.3461) is shown. Initial tree(s) for the heuristic search were obtained by applying the Neighbor-Joining method to a matrix of pairwise distances estimated using a JTT model. The tree is drawn to scale, with branch lengths measured in the number of substitutions per site. The analysis involved 29 amino acid sequences. All positions containing gaps and missing data were eliminated. There were a total of 600 positions in the final dataset. Evolutionary analyses were conducted in MEGA6.

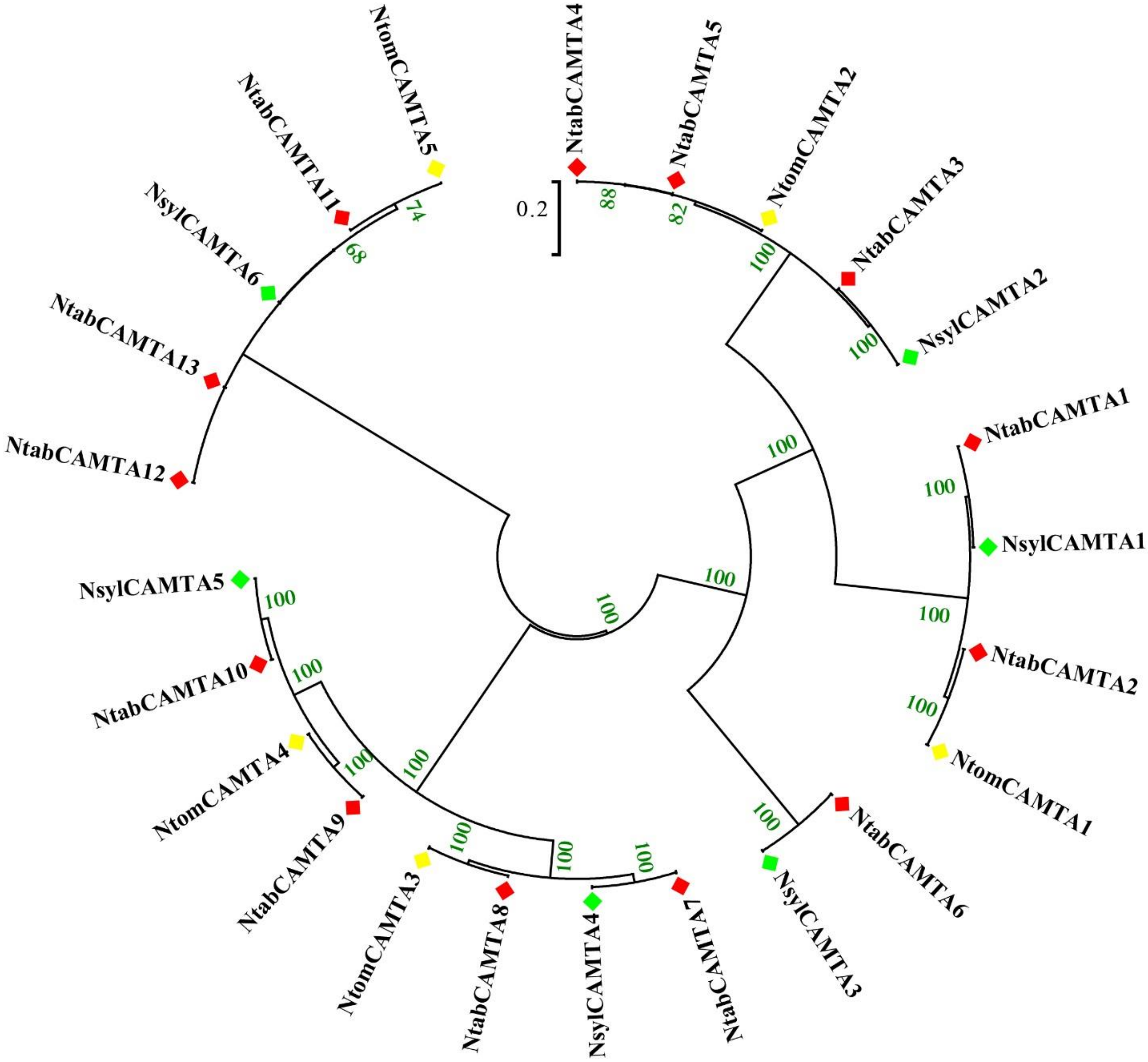

Supplementary Figure S7. Gene structure showing the exon-intron organisation and their phases within the transcripts of *CAMTA* family genes from four *Nicotiana* species. The NJ phylogenetic tree of CDs is shown on the left side of the figure, while, exons and introns are indicated as red boxes and blue lines on the right. Numbers [0, 1 and 2] given on gene structures represent their respective intron phases. The lengths of each exon and intron can be mapped to the scale given in the bottom.

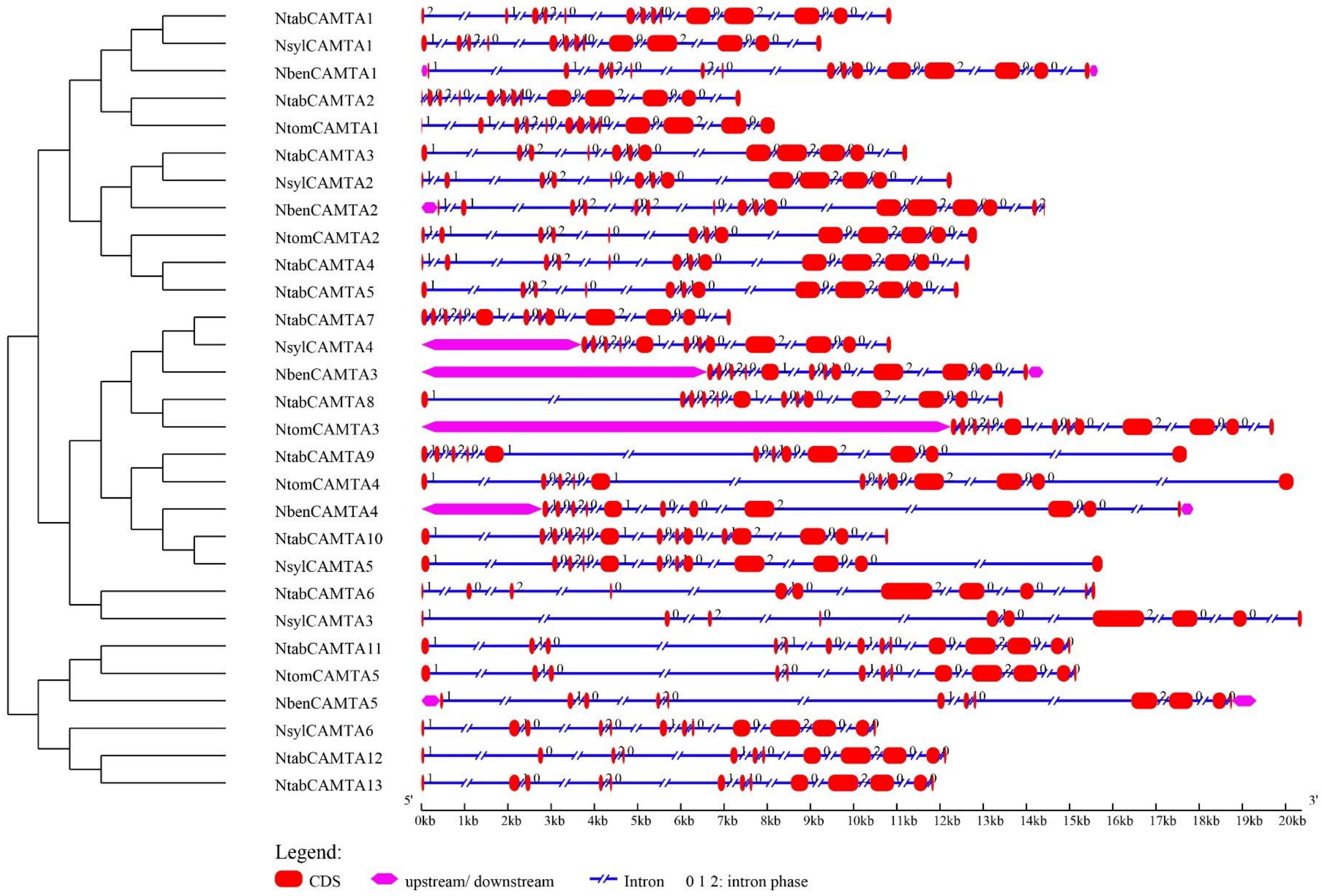

Supplementary Figure S8. Schematic diagram showing the structures of 6 *AtCAMTA* genes. The NJ phylogenetic tree of CDs is shown on the left side of the figure, while, exons and introns are indicated as red boxes and blue lines on the right. Numbers [0, 1 and 2] given on gene structures represent their respective intron phases. The lengths of each exon and intron can be mapped to the scale given in the bottom.

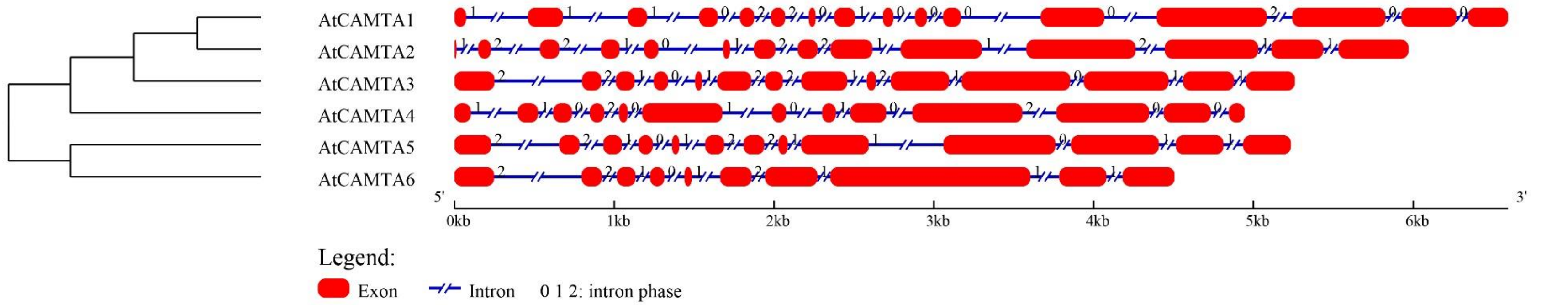

Supplementary Figure S9. **Schematic diagram showing the logos of functionally annotated conserved motifs of *NtabCAMTA*-encoded proteins.**

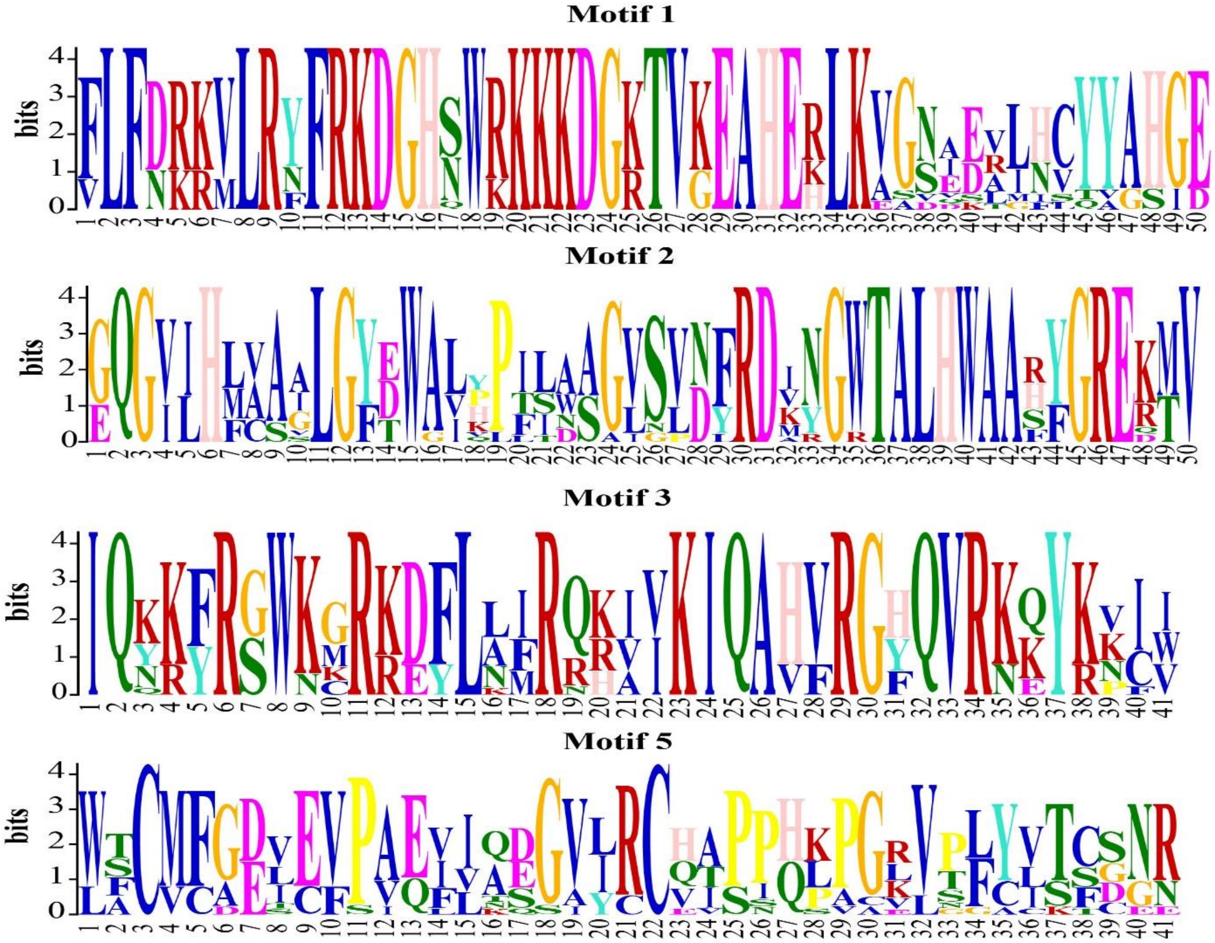

Supplementary Table 1. List of discarded gene accessions with truncated sequences or absence of *CAMTA*-specific domains.

Species	Accession	Position	Start	Stop	Strand	Exon	Nucleotide length	Protein length	CG-1	IPT/TIG	ANK	IQ
N. benthamiana	Niben101Scf05099g00009.1	Niben101Scf05099	255	4198	+	5	2660	674	-	-	1	3
	Niben101Scf09203g01025.1	Niben101Scf09203	105081	122160	-	14	4516	656	-	-	1	3
	Niben101Scf03049g02001.1	Niben101Scf03049	223588	240754	-	7	1234	339	1	-	-	-
	Niben101Scf23901g00005.1	Niben101Scf23901	12790	17700	-	7	1973	604	-	1	2	2
N. tabacum	Ntab0966680	Chr09	40792657	40804621	+	8	1098	365	1	-	-	-
	Ntab0503030	Chr06	15543250	15550312	-	11	1878	625	1	1	-	-

Supplementary Table S2. Domains analysis of CAMTA genes identified in this study.

Gene name	Accession	CG-1	IPT/TIG	ANK	IQ
NtabCAMTA8	Ntab0019010	1	1	1	3
NtabCAMTA11	Ntab0045050	1	-	1	3
NtabCAMTA6	Ntab0114010	1	-	2	2
NtabCAMTA1	Ntab0183780	1	1	3	2
NtabCAMTA10	Ntab0354250	1	1	2	3
NtabCAMTA13	Ntab0368180	1	-	1	3
NtabCAMTA3	Ntab0473890	1	1	2	2
NtabCAMTA2	Ntab0553680	1	1	3	2
NtabCAMTA5	Ntab0695280	1	1	2	2
NtabCAMTA4	Ntab0695330	1	1	2	2
NtabCAMTA7	Ntab0794220	1	1	1	3
NtabCAMTA12	Ntab0797190	1	-	1	3
NtabCAMTA9	Ntab0852870	1	1	2	3
AtCAMTA1	AT5G09410.3	1	1	1	2
AtCAMTA3	AT2G22300.1	1	1	2	2
AtCAMTA4	AT1G67310.1	1	1	2	3
AtCAMTA2	AT5G64220.1	1	-	2	2
AtCAMTA6	AT3G16940	1	1	1	3
AtCAMTA5	AT4G16150	1	1	1	3
NsylCAMTA4	Nsyl0096530.1	1	1	1	3
NsylCAMTA5	Nsyl0483140.1	1	1	2	3
NsylCAMTA1	Nsyl0348080.1	1	1	3	2
NsylCAMTA2	Nsyl0152020	1	1	2	2
NsylCAMTA3	Nsyl0198310.1	1		2	2
NsylCAMTA6	Nsyl0255340.1	1		1	3
NtomCAMTA3	Ntom0193400.1	1	1	1	3
NtomCAMTA4	Ntom0275970.1	1	1	2	3
NtomCAMTA1	Ntom0062140.1	1	1	3	2
NtomCAMTA2	Ntom0118080.1	1	1	2	2

NtomCAMTA5	Ntom0150930.1	1		1	3
NbenCAMTA3	Niben101Scf03911g05003.1	1	1	1	3
NbenCAMTA4	Niben101Scf01740g07004.1	1	1	1	3
NbenCAMTA1	Niben101Scf07773g00004.1	2	1	3	2
NbenCAMTA2	Niben101Scf02268g06007.1	2	1	2	2
NbenCAMTA5	Niben101Scf00380g00001.1	1		1	3

Supplementary Table S3. List of orthologous of tobacco *CAMTAs* from all plant lineages used to construct phylogenetic tree

Plant specie	**Protein ID**	**Similarity (%)**	**Alignment length**	**E value**	**Gene function**
Amborella trichopoda	evm_27.TU.AmTr_v1.0_scaffold00038.113	100	740	0	
Aquilegia coerulea	Aquca_010_00201.2	45.12	1075	0	signal responsive 1
Arabidopsis lyrata	919730	46.65	1059	0	
Arabidopsis thaliana	AT5G64220.2	47.04	1063	0	Calmodulin-binding transcription activator protein with CG-1 and Ankyrin domains
Brachypodium distachyon	Bradi1g21372.1.p	43.85	1024	0	ethylene induced calmodulin binding protein
Brassica napus	GSBRNA2T00135980001	46.07	1055	0	
Brassica oleracea	Bol043749	46.08	1046	0	
Brassica rapa	Bra009382	45.97	1055	0	
Boechera stricta	Bostr.0568s0382.1.p	46.94	1061	0	
Beta vulgaris	BVRB_9g215020	48.42	1045	0	
Cicer arietinum	XP_004485583.1	49.14	1046	0	PREDICTED: calmodulin-binding transcription activator 2-like isoform X2
Cajanus cajan	C.cajan_22901	49.19	1053	0	
Coffea canephora	Cc02_g21520	58.86	1033	0	Calmodulin-binding transcription activator 2
Citrus clementina	Ciclev10024771m	46.03	1058	0	signal responsive 1
Capsella grandiflora	Cagra.0248s0103.1.p	48.25	1030	0	Calmodulin-binding transcription activator protein with CG-1 and Ankyrin domains
Cucumis melo	MELO3C018642P2	47.92	1081	0	

Carica papaya	evm.model.supercontig_213.8	55.75	565	0	signal responsive 1
Chlamydomonas reinhardtii	Cre16.g674300.t1.1	30.7	114	1.00E-07	ankyrin repeat-containing protein 2
Capsella rubella	Carubv10025787m	47.91	1054	0	Calmodulin-binding transcription activator protein with CG-1 and Ankyrin domains
Cucumis sativus	Cucsa.217940.1	48.8	1041	0	Calmodulin-binding transcription activator protein with CG-1 and Ankyrin domains
Citrus sinensis	orange1.1g001406m	51.37	1057	0	Calmodulin-binding transcription activator protein with CG-1 and Ankyrin domains
Coccomyxa subellipsoidea	61775	30.2	351	2.00E-27	ethylene induced calmodulin binding protein
Eucalyptus grandis	Eucgr.H04783.1	46.82	1038	0	signal responsive 1
Elaeis guineensis	XP_010926293.1	45.71	1050	0	hypothetical protein SORBIDRAFT_01g044480
Eutrema salsugineum	Thhalv10012552m	46.4	1041	0	ethylene induced calmodulin binding protein
Fragaria vesca	mrna16296.1-v1.0-hybrid	43.4	1106	0	signal responsive 1
Gossypium hirsutum	CotAD_33349	48.43	1086	0	
Glycine max	Glyma.05G117000.1.p	48.5	1070	0	Calmodulin-binding transcription activator protein with CG-1 and Ankyrin domains
Gossypium raimondii	Gorai.013G061100.1	48.79	1074	0	Calmodulin-binding transcription activator protein with CG-1 and Ankyrin domains
Linum usitatissimum	Lus10003405	46.6	1103	0	Calmodulin-binding transcription activator protein with CG-1 and Ankyrin domains
Musa acuminata	GSMUA_Achr6T10390_001	46.27	1046	0	Putative Calmodulin-binding transcription activator 3

Malus domestica	MDP0000255517	49.22	1030	0	Calmodulin-binding transcription activator protein with CG-1 and Ankyrin domains
Manihot esculenta	cassava4.1_000687m	48.6	1072	0	Calmodulin-binding transcription activator protein with CG-1 and Ankyrin domains
Mimulus guttatus	Migut.B00609.1.p	55.98	1029	0	ethylene induced calmodulin binding protein
Medicago truncatula	Medtr2g008840.4	48.75	1044	0	calmodulin-binding transcription activator 1
Musa acuminata	GSMUA_Achr6P10390_001	46.27	1046	0	Putative Calmodulin-binding transcription activator 3
Nicotiana tabacum	Ntab0183780,MF142771				
Nicotiana tomentosiformis	Ntom0062140				
Nicotiana sylvestris	Nsyl0348080				
Nicotiana benthamiana	Niben101Scf07773g00004				
Oryza sativa	LOC_Os03g09100.1	49.76	635	0	calmodulin-binding transcription activator, putative, expressed
Pyrus bretschneideri	Pbr028458.1	50.24	1021	0	
Phyllostachys heterocycla	PH01001360G0240	51.42	669	0	calmodulin-binding transcription activator, putative, expressed
Physcomitrella patens	Pp3c8_2690V3.1	40.48	667	1.00E-131	Calmodulin-binding transcription activator protein with CG-1 and Ankyrin domains
Prunus persica	ppa000612m	45.26	1096	0	signal responsive 1
Populus trichocarpa	Potri.005G075100.2	45.65	1093	0	signal responsive 1
Panicum virgatum	Pavir.Ba00386.1.p	44.61	1002	0	Calmodulin-binding transcription activator protein with CG-1 and Ankyrin domains
Phaseolus vulgaris	Phvul.002G209300.1	48.11	1083	0	Calmodulin-binding transcription activator

					protein with CG-1 and Ankyrin domains
Ricinus communis	30063.m001397	45.32	1079	0	Calmodulin-binding transcription activator protein with CG-1 and Ankyrin domains
Sorghum bicolor	Sobic.002G380200.2.p	43.84	990	0	signal responsive 1
Setaria italica	Si028797m	44.67	1003	0	ethylene induced calmodulin binding protein
Solanum lycopersicum	Solyc01g105230.2.1	84.96	1017	0	Calmodulin-binding transcription activator 2 IPR005559 CG-1
Solanum melongena	Sme2.5_00225.1_g00014.1	85.35	1017	0	
Selaginella moellendorffii	106391	41.73	623	8.00E -136	signal responsive 1
Solanum pimpinellifolium	Sopim01g105230	84.96	1017	0	Calmodulin-binding transcription activator 2 (AHRD V1 **** Q0WNN4_ARATH); contains Interpro domain(s) IPR005559 CG-1
Salix purpurea	SapurV1A.0403s0170.1.p	45.66	1084	0	signal responsive 1
Theobroma cacao	Thecc1EG007482t1	49.68	1085	0	Calmodulin-binding transcription activator protein with CG-1 and Ankyrin domains
Volvox carteri	Vocar20002845m	31.3	115	5.00E -06	ankyrin repeat family protein
Vitis vinifera	GSVIVT01004860001	46.65	1089	0	signal responsive 1
Zea mays	GRMZM2G431243_P01	43.78	989	0	

Supplementary Table S4. Annotation of conserved MEME motifs identified in a set of 13 NtabCAMTA proteins.

Motif	Width	Sequence	E-value	Sites	Pfam annotation	other Databases
1	43	VGNIEVLHCYYAHGEENPNFQRRCYWMLDPEMEHIVLVHYREV	1.2e-374	13	CG-1	
2	59	VWDEKGQGVIHMCAALGYDWALYPILWAGVSVNFRDMNGWTALHWAAHYGREKMVAFLI	3.5e-436	13	Ankyrin repeat profile	
3	50	FYNMRPYHTAAVRIQKKFRGWKMRKDFLNMRQKIIKIQAHVRGHQVRKQY	2.0e-369	13	IQ calmodulin-binding motif	
4	41	PPSGSMFLFDRKVLRYFRKDGHNWRKKKDGKTVKEAHERLK	1.5e-364	13	CG-1 domain	
5	44	WTCMFGDICVPAEIIQDGVIRCHAPPHKPGRVPFYVTCCNRLAC	4.9e-274	13	IPT/TIG domain	casein kinase II (CK2), protein kinase C
6	57	SLGAAPGAVTDPTPEHPGGRTPADLASSCGHKGIAGYLAEVALTAHLSSMTLKEMKQ	5.8e-328	13	unkown	
7	41	DWEDQTSLKDTLAAVRNAAQAAARIHAMFRFHSFQRRQQRM	7.2e-265	20	unknown	COG0773, MurC, UDP-N-acetylmuramate-alanine ligase [Cell envelope biogenesis, outer membrane]
8	41	MSPSYSQKQIFTIIDISPDWAYSSEETKVLIIGHFLCNQPE	1.1e-221	13	Unknown	PRK09219, xanthine phosphoribosyltransferase
9	29	KICWSVGILEKVILRWRRKGVGLRGFRPE	9.3e-210	13	Unknown	Unknown
10	37	EARMQKALARVKSMVQYPEARQQYHRILNKYRQMKET	1.1e-137	10	Protein of unknown function (DUF3151)	Unknown

Supplementary Table S5. The potential miRNA targets in the set of 13 *NtabCAMTA* transcripts with cut-off threshold of 4.5.

miRNA Acc.	Target Acc.	Expectation (E)	Target Accessibility (UPE)	Alignment	Inhibition	Multiplicity
**nta-miR159**	NtabCAMTA2	4.0	20.486	miRNA 20 UCUCGAGGGAAGUUAGGUUU 1<br>::::.:.:.::.:::::..<br>Target 2550 AGAGUUUCUUUUAAUCCGGC 2569	Cleavage	1
**nta-miR394**	NtabCAMTA9	4.0	20.833	miRNA 20 CCUCCACCUGUCUUACGGUU 1<br>:::: ::::: ::::::<br>Target 2920 GGAGGAGGACAUCAUGCCAU 2939	Translation	1
**nta-miR395a**	NtabCAMTA2	4.5	23.019	miRNA 20 UCAAGGGGGUUUGUGAAGUC 1<br>.:::::: ::.: ..::.::<br>Target 1398 GGUUCCCGCAGAGGUUUUAG 1417	Cleavage	1
**nta-miR395b**	NtabCAMTA2	4.5	23.019	miRNA 20 UCAAGGGGGUUUGUGAAGUC 1<br>.:::::: ::.: ..::.::<br>Target 1398 GGUUCCCGCAGAGGUUUUAG 1417	Cleavage	1
**nta-miR395c**	NtabCAMTA2	4.5	23.019	miRNA 20 UCAAGGGGGUUUGUGAAGUC 1<br>.:::::: ::.: ..::.::<br>Target 1398 GGUUCCCGCAGAGGUUUUAG 1417	Cleavage	1
**nta-miR477a**	NtabCAMTA6	4.5	18.36	miRNA 21 GUCUUCGGGAACUCCCUCUCA 1<br>::::.:: : :::::::::<br>Target 2371 CAGAGGCAGCUUAGGGAGAGU 2391	Translation	1
**nta-miR6020b**	NtabCAMTA11	4.5	10.491	miRNA 20 UUCUAUGAGCUUCUUGUAAA 1<br>:::: .::: : ::::::::<br>Target 392 AAGAAGCUC-AUGAACAUUU 410	Translation	1
**nta-miR6163**	NtabCAMTA11	4.5	22.015	miRNA 22 AGUUUGAAUCCGUCAUGAAGGU 1<br>:.: :::.:::: :::. ::::<br>Target 2046 UUAUACUUGGGCUGUAUAUCCA 2067	Translation	1
**nta-miR6163**	NtabCAMTA13	4.5	22.009	miRNA 22 AGUUUGAAUCCGUCAUGAAGGU 1<br>:.: :::.:::: :::. ::::<br>Target 1890 UUAUACUUGGGCUGUAUAUCCA 1911	Translation	1
**nta-miR6163**	NtabCAMTA12	4.5	22.009	miRNA 22 AGUUUGAAUCCGUCAUGAAGGU 1<br>:.: :::.:::: :::. ::::<br>Target 1653 UUAUACUUGGGCUGUAUAUCCA 1674	Translation	1
**nta-miR6163**	NtabCAMTA1	4.5	19.401	miRNA 20 UUUGAAUCCGUCAUGAAGGU 1<br>::.:: : :::: :.:::.:<br>Target 1942 AAGCUGAAGCAGAAUUUCUA 1961	Cleavage	1
**nta-miR6163**	NtabCAMTA2	4.5	24.42	miRNA 20 UUUGAAUCCGUCAUGAAGGU 1<br>::.:: : :::: :.:::.:<br>Target 1792 AAGCUGAAGCAGAAUUUCUA 1811	Cleavage	1

Supplementary Table S6. GO term enrichment analysis of *NtabCAMTA* genes for category: Cellular component.

GO term	Name	Probability	SVM Reliability
**GO:0016020**	membrane	0.698	H
**GO:0005886**	plasma membrane	0.565	H
**GO:0043229**	intracellular organelle	0.94	L
**GO:0043231**	intracellular membrane-bounded organelle	0.899	L
**GO:0005737**	cytoplasm	0.886	L
**GO:0005634**	nucleus	0.771	L
**GO:0031981**	nuclear lumen	0.708	L
**GO:0005829**	cytosol	0.689	L
**GO:0005654**	nucleoplasm	0.65	L
**GO:0043234**	protein complex	0.622	L

Supplementary Table S7. GO term enrichment analysis of *NtabCAMTA* genes for category: Molecular function.

GO term	Name	Probability	SVM Reliability
GO:0003824	catalytic activity	0.964	H
GO:0030554	adenyl nucleotide binding	0.91	H
GO:0008092	cytoskeletal protein binding	0.87	H
GO:0016773	phosphotransferase activity, alcohol group as acceptor	0.827	H
GO:0017076	purine nucleotide binding	0.822	H
GO:0032549	ribonucleoside binding	0.813	H
GO:0005524	ATP binding	0.806	H
GO:0031267	small GTPase binding	0.795	H
GO:0051020	GTPase binding	0.795	H
GO:0035639	purine ribonucleoside triphosphate binding	0.784	H
GO:0004672	protein kinase activity	0.733	H
GO:0001882	nucleoside binding	0.722	H
GO:0016740	transferase activity	0.693	H
GO:0017016	Ras GTPase binding	0.688	H
GO:0004674	protein serine/threonine kinase activity	0.673	H
GO:0019900	kinase binding	0.658	H
GO:0016301	kinase activity	0.653	H
GO:0005096	GTPase activator activity	0.601	H
GO:0019901	protein kinase binding	0.59	H
GO:0000166	nucleotide binding	0.59	H
GO:0001883	purine nucleoside binding	0.587	H
GO:0003779	actin binding	0.576	H
GO:0030234	enzyme regulator activity	0.566	H
GO:0008270	zinc ion binding	0.549	H
GO:0016817	hydrolase activity, acting on acid anhydrides	0.537	H
GO:0097159	organic cyclic compound binding	0.87	L
GO:0036094	small molecule binding	0.803	L
GO:0005102	receptor binding	0.762	L
GO:0032403	protein complex binding	0.754	L
GO:0019904	protein domain specific binding	0.597	L
GO:0016772	transferase activity, transferring phosphorus-containing groups	0.56	L
GO:0043169	cation binding	0.541	L

Supplementary Table S8. GO term enrichment analysis of *NtabCAMTA* genes for category: Biological process.

GO term	Name	Probability	SVM Reliability
GO:0019222	regulation of metabolic process	0.848	H
GO:0010468	regulation of gene expression	0.646	H
GO:0006810	transport	0.64	H
GO:0051649	establishment of localization in cell	0.638	H
GO:0008104	protein localization	0.636	H
GO:0006796	phosphate-containing compound metabolic process	0.621	H
GO:1903506	regulation of nucleic acid-templated transcription	0.531	H
GO:0043087	regulation of GTPase activity	0.524	H
GO:0045184	establishment of protein localization	0.516	H
GO:0035556	intracellular signal transduction	0.509	H
GO:0051641	cellular localization	0.503	H
GO:0007165	signal transduction	0.858	L
GO:0023052	signaling	0.842	L
GO:0008152	metabolic process	0.809	L
GO:0044237	cellular metabolic process	0.803	L
GO:0050896	response to stimulus	0.8	L
GO:0051716	cellular response to stimulus	0.798	L
GO:0007154	cell communication	0.789	L
GO:0009893	positive regulation of metabolic process	0.733	L
GO:0034641	cellular nitrogen compound metabolic process	0.73	L
GO:0006725	cellular aromatic compound metabolic process	0.725	L
GO:0048856	anatomical structure development	0.725	L
GO:0046483	heterocycle metabolic process	0.704	L
GO:0006996	organelle organization	0.7	L
GO:0007275	multicellular organismal development	0.696	L
GO:0009966	regulation of signal transduction	0.692	L
GO:0032502	developmental process	0.661	L
GO:0019538	protein metabolic process	0.629	L
GO:0044267	cellular protein metabolic process	0.598	L
GO:0030154	cell differentiation	0.583	L
GO:0031325	positive regulation of cellular metabolic process	0.576	L
GO:0010467	gene expression	0.57	L
GO:0050790	regulation of catalytic activity	0.528	L
GO:0016070	RNA metabolic process	0.523	L
GO:0006464	cellular protein modification process	0.504	L

Supplementary Table S9. List of primers used for qRT-PCR analysis.

Genes	Primer (5`-3`)	Strand	Position	Tm °C	Amplicon Size
NtabACTIN	AGCACATTCCAACAGGTGAG	forward		58.01	149
	GGAAGGACCAGACTCATCATAC	reverse		57.50	
NtabCAMTA1	CCAATGCGGAGTGAAAGAGG	forward	1905	56.01	151
	AAGTGCAGCTGCCAAATGAA	reverse	2036	56.00	
NtabCAMTA2	CCAATGCGGAGTGAAAGAGG		1755	56.01	151
	AAGTGCAGCTGCCAAATGAA		1886	56.00	
NtabCAMTA3	TGGCGTCTTGAGAGAAGGTT	forward	1329	55.98	174
	TCTAACTGCACTTGCGAAGC	reverse	1483	55.98	
NtabCAMTA4	TGGCCACATGGAGTCTCATT	forward	2575	55.99	113
	AGCTGTTTCCTCTGGAACGA	reverse	2668	55.98	
NtabCAMTA5	TGGCGTCTTGAGAGAAGGTT	forward	1293	55.98	174
	TCTAACTGCACTTGCGAAGC	reverse	1447	55.98	
NtabCAMTA6	ATTCTGCGTTGGAGGAGGAA	forward	2650	56.02	197
	TGGTCTCTTGCCTCTGGATG	reverse	2827	56.11	
NtabCAMTA7	CCCTCACATTGGTGGAGAGT	forward	2084	56.0	189
	AATGTGCTCGGAATGCAGAC	reverse	2253	56.01	
NtabCAMTA8	GTCTGCATTCCGAGCACATT	forward	2391	56.01	144
	GTATTGCGGAATGCCCACTT	reverse	2515	55.97	
NtabCAMTA9	ACAATCTGCATTCCGAGCAC	forward	2283	56.01	144
	TTGCGGAATGCCAACTTTGA	reverse	2407	56.02	
NtabCAMTA10	ACCCATCGAGGAAAGTGAGG	forward	2721	56.12	129
	TATTGTTGGCGTGCTTCTGG	reverse	2830	55.93	
NtabCAMTA11	CACCAGACCTGAGAGAACGA	forward	2605	56.09	105
	GCTGCTGCACGAATCCAATA	reverse	2690	56.07	
NtabCAMTA12	AGCCTAACAGGGATGTGACC	forward	479	56.09	129
	TTCCTCCCGCTTCTTGAGTT	reverse	588	55.95	
NtabCAMTA13	TGGTTGGACAGCTCTTCACT	forward	1953	55.87	120
	CCACCAGGGTTCTCTGAAGT	reverse	2053	55.94	
